# Single-Standard Quantification Strategy for Lignin Dimers by Supercritical Fluid Chromatography with Charged Aerosol Detection

**DOI:** 10.1021/acs.analchem.2c04383

**Published:** 2022-12-22

**Authors:** Daniel Papp, Thanya Rukkijakan, Daria Lebedeva, Tommy Nylander, Margareta Sandahl, Joseph S. M. Samec, Charlotta Turner

**Affiliations:** † Lund University, Department of Chemistry, Centre for Analysis and Synthesis, P.O. Box 124, SE-22100 Lund, Sweden; ‡ Stockholm University, Department of Organic Chemistry, Svante Arrhenius väg 16C, SE-106 91 Stockholm, Sweden; § Lund University, Department of Chemistry, Physical Chemistry, P.O. Box 124, SE-22100 Lund, Sweden

## Abstract

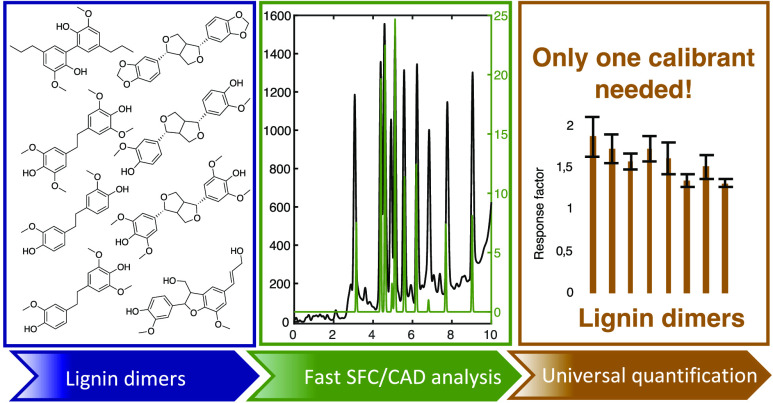

The increased interest in utilizing lignin as a feedstock to produce various aromatic compounds requires advanced chemical analysis methods to provide qualitative and quantitative characterization of lignin samples along different technology streamlines. However, due to the lack of commercially available chemical standards, routine quantification of industrially relevant lignin oligomers in complex lignin samples remains a challenge. This study presents a novel method for universal quantification of lignin dimers based on supercritical fluid chromatography with charged aerosol detection (CAD). A series of lignin-derived dimeric compounds that have been reported from reductive catalytic fractionation (RCF) were synthesized and used as standards. The applicability of using linear regression instead of quadratic calibration curves was evaluated over a concentration range of 15–125 mg/L, demonstrating that the former calibration method is as appropriate as the latter. The response factors of lignin dimeric compounds were compared to assess the uniformity of the CAD signal, revealing that the CAD response for the tested lignin dimers did not differ substantially. It was also found that the response factors were not dependent on the number of methoxy groups or linkage motifs, ultimately enabling the use of only one calibrant for these compounds. The importance of chromatographic peak resolution in CAD was stressed, and the use of a digital peak sharpening technique was adopted and applied to address this challenge. The developed method was verified and used for the quantification of lignin dimers in an oil obtained by a RCF of birch sawdust.

## Introduction

Lignin is the second most abundant biopolymer in biomass and is produced in high quantities in paper and pulping industries. It has been considered a low-value byproduct; however, the interest in the conversion of lignin to value-added phenolic compounds has increased lately.^1−4^ Valorization of lignin will become important when transitioning from a fossil-based society to a bio-based society. New fractionation approaches of lignocellulosic biomass have emerged, which also consider valorization of lignin in addition to cellulose during the last decade.^5,6^ These fractionation methods have been termed “lignin-first” approaches, in which reductive catalytic fractionation (RCF) is the most reported direction.^7−12^ RCF generally transforms around 40% of the lignin in hardwoods to monophenolic compounds that are easily separated and quantitatively determined by gas chromatography with flame ionization detection (GC-FID) as well as a mixture of mainly dimers and smaller amounts of other oligomers. Although identification of RCF dimers and oligomers has been reported several times,^13,14^ quantitative studies, which are crucial for the understanding and designing of more efficient valorization strategies,^10^ are scarce. A recent publication addressed the challenge via GC × GC-FID analysis after silylation of the sample to increase the volatility of dimers and other oligomers.^15^ However, such derivatization is usually undesired as it introduces an additional step in the analysis workflow and a possible bias due to incomplete reactions.^16,17^ Moreover, FID is not a universal detector, although historically, it has been a common approach to assume uniform response factors even for analytes with diverse structural motifs.^18^ Thus, accurate quantification demands the respective chemical standards or a well-performing prediction model for the establishment of the calibration functions.

Since regression models often suffer from systematic errors and require a large amount of data to train on, universal detectors such as a charged aerosol detector or an evaporative light scattering detector, which are available for liquid chromatography (LC),^19^ are better options to tackle the low availability of commercial dimer standards; however, signal uniformity has to be more thoroughly evaluated. Hutchinson et al. demonstrated that the CAD response heavily depends on analyte volatility and suggested that the detector is universal only for analytes with boiling points above 400 °C.^20^ Another study conducted by Robinson et al. concluded that the CAD response is heavily dependent on the surface area of the analyte.^21^

While CAD has been used for a wide range of applications with LC separation,^22,23^ its coupling to supercritical fluid chromatography (SFC) is still yet to be more thoroughly explored. SFC/CAD offers rapid and highly selective separation^24−26^ with hypothetically universal detection as demonstrated by Takeda et al. for lipid analysis.^27^ However, to our knowledge, no such studies for quantification of lignin-derived compounds in general or dimers from a RCF in particular have been published. While most lignin monomers are too volatile to be detected by the CAD, our hypothesis is that lignin dimers have sufficiently low volatility to yield a uniform response.

**Figure 1:**
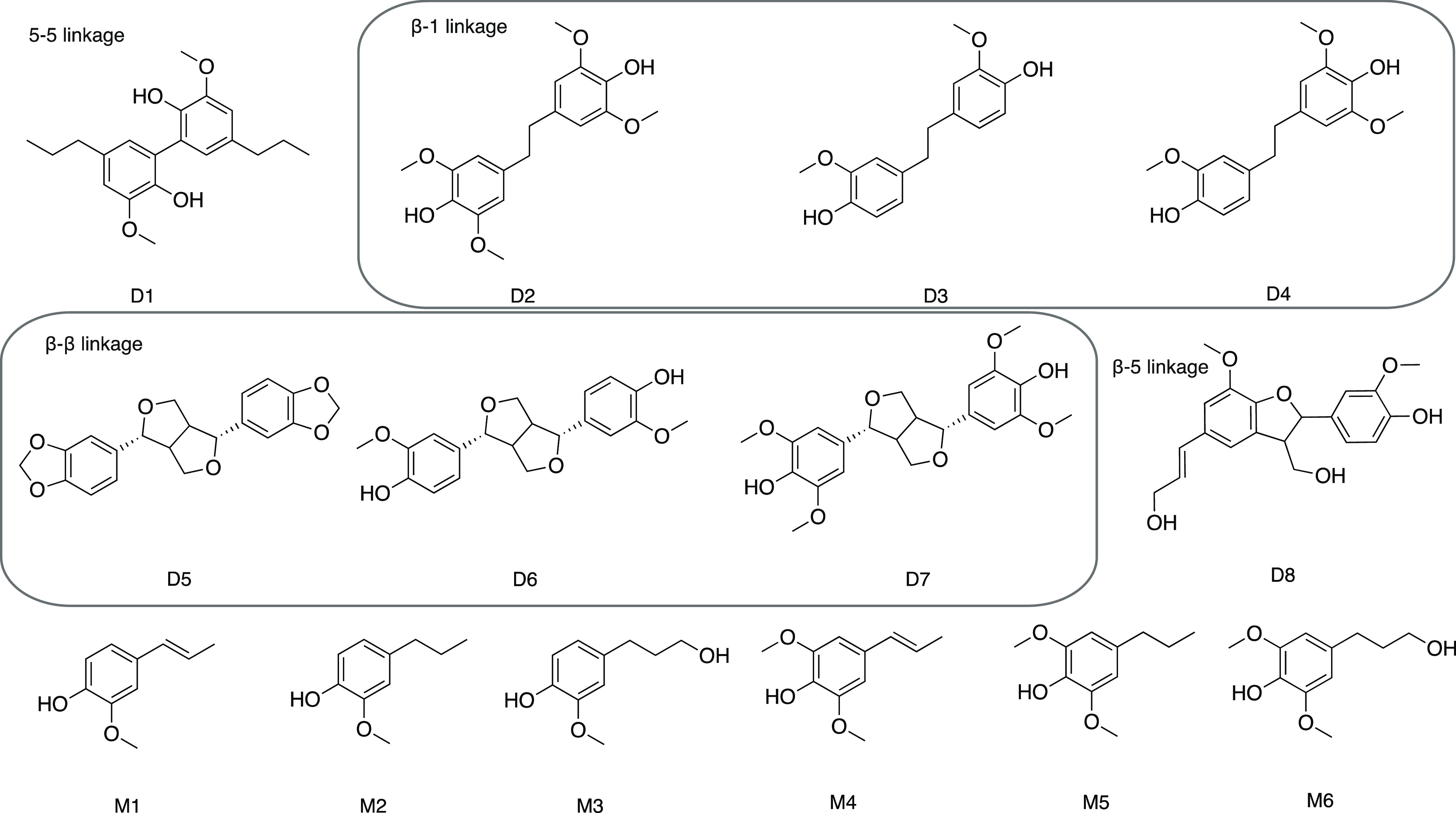
Structures of lignin reference materials synthesized and used in this study. D1: 3,3′-dimethoxy-5,5′-dipropylbiphenyl-2,2′-diol (dehydrodidihydroeugenol); D2: 4-[2-(4-hydroxy-3,5-dimethoxyphenyl)ethyl]-2,6-dimethoxyphenol; D3: 4,4′-(ethane-1,2-diyl)bis(2-methoxyphenol); D4: 4-(4-hydroxy-3-methoxyphenethyl)-2,6-dimethoxyphenol; D5: 5-[(3*S*,3*aR*,6*S*,6*aR*)-3-(1,3-benzodioxol-5-yl)-1,3,3*a*,4,6,6*a*-hexahydrofuro[3,4-*c*]furan-6-yl]-1,3-benzodioxole (sesamin); D6: 4-[(3*S*,3*aR*,6*S*,6*aR*)-6-(4-hydroxy-3-methoxyphenyl)-1,3,3*a*,4,6,6*a*-hexahydrofuro[3,4-*c*]furan-3-yl]-2-methoxyphenol (pinoresinol); D7: 4-[6-(4-hydroxy-3,5-dimethoxyphenyl)-1,3,3*a*,4,6,6*a*-hexahydrofuro[3,4-*c*]furan-3-yl]-2,6-dimethoxyphenol (syringaresinol); D8: (*E*)-4-(3-(hydroxymethyl)-5-(3-hydroxyprop-1-en-1-yl)-7-methoxy-2,3-dihydrobenzofuran-2-yl)-2-methoxyphenol; M1: 2-methoxy-4-[(*E*)-prop-1-enyl]phenol (isoeugenol); M2: 2-methoxy-4-propylphenol; M3: 4-(3-hydroxypropyl)-2-methoxyphenol (dihydroconiferyl alcohol); M4: (*E*)-2,6-dimethoxy-4-(prop-1-en-1-yl)phenol; M5: 2,6-dimethoxy-4-propylphenol; and M6: 4-(3-hydroxypropyl)-2,6-dimethoxyphenol.

In this study, SFC/CAD is used for the first time as a rapid and highly selective analysis method with universal detection to quantify lignin dimers in a complex birch sawdust lignin oil sample. Hardwood lignin comprise both guaiacol (G-units) and syringol (S-units) structural motifs, that is, containing one or two methoxy groups, respectively. RCF of hardwood mainly generates β-1 and β–β binding motifs. Hence, in this study, eight dimers have been synthesized, of which three of each were with β-1 and β–β binding motifs and with varying number of methoxy groups (Figure 1). These compounds were then investigated for response uniformity in the CAD. In order to counterweigh post-column broadening before the CAD, digital techniques for apparent resolution enhancement^28^ were employed. The classification of lignophenolics chromatographic peaks in SFC/CAD was carried out by SFC coupled to quadrupole-time-of-flight MS (QToF-MS) based on a previous work in our research group.^26^

## Experimental Section

### Chemicals

Carbon dioxide (99.9993%; Linde, Guildford, UK), methanol (Optima LC/MS Grade; Fisher Scientific, Loughborough, UK), acetone (HiPerSolv CHROMANORM; VWR, Radnor, PA, USA), and tetrahydrofuran (THF) (HiPerSolv CHROMANORM; VWR, Radnor, PA, USA) were purchased from the respective manufacturers. Ammonia solution (2 M, in methanol) was purchased from Fisher Scientific (Loughborough, UK). Ultrapure water was purified in-house by a Merck Millipore water purification system (Millipore, Billerica, MA, USA).

### Production and Purification of Lignin Reference Materials

Dehydrodidihydroeugenol (D1), 4-[2-(4-hydroxy-3,5-dimethoxyphenyl)ethyl]-2,6-dimethoxyphenol (D2), 4,4′-(ethane-1,2-diyl)bis(2-methoxy-phenol) (D3), 4-(4-hydroxy-3-methoxyphenethyl)-2,6-dimethoxy-phenol (D4), sesamin (D5), pinoresinol (D6), syringaresinol (D7), (*E*)-4-(3-(hydroxymethyl)-5-(3-hydroxyprop-1-en-1-yl)-7-methoxy-2,3-dihydrobenzofuran-2-yl)-2-methoxyphenol (D8), isoeugenol (M1), 2-methoxy-4-propylphenol (M2), dihydroconiferyl alcohol (M3), (*E*)-2,6-dimethoxy-4-(prop-1-en-1-yl)phenol (M4), 2,6-dimethoxy-4-propylphenol (M5), and 4-(3-hydroxypropyl)-2,6-dimethoxyphenol (M6) (see Figure 1) were synthesized using literature procedures which are disclosed in detail including full characterization [^1^H, ^13^C NMR, and high-resolution mass spectrometry (HRMS)] in the Supporting Information S1. Lignin oil containing dimers was produced by RCF of birch sawdust using Pd/C as a catalyst.^29,30^

### Production and Characterization of Lignin Oil

Birch sawdust (mix of *Betula pendula* and *Betula pubescens*) was provided by Vanhälls Såg AB. Detailed analysis of the material was reported by Kumaniaev et al.^31^

^1^H NMR spectra were recorded with a Bruker 400 (400 MHz) spectrometer as solutions. Chemical shifts are expressed in parts per million (ppm, δ) and are referenced to a deuterated solvent as an internal standard. All coupling constants are absolute values and are expressed in hertz. ^13^C NMR spectra were recorded with a Bruker 400 (101 MHz) spectrometer as solutions. HRMS spectra were acquired with a micrOTOF (Bruker) spectrometer.

Unless otherwise stated, all chemicals were purchased from Merck. Sesame oil (ICA Asia), used for the isolation of sesamin, was purchased from ICA supermarket in Sweden.

A mixture of birch sawdust (1 g) and Pd/C (100 mg, 5 wt % of Pd) was loaded into a stainless steel reactor (Swagelock) and filled with a solution of water (6 mL) and ethanol (6 mL). After closing the reactor tightly, the reaction was stirred at 200 °C for 2 h. Then, the reactor was allowed cool to room temperature. The reaction mixture was filtered by filter paper to remove Pd/C and the pulp. The filtrate was concentrated by a rotary evaporator to afford a crude product as a brown oil. The crude product was diluted with water and extracted with dichloromethane. The combined organic phase was dried with Na_2_SO_4_, filtrated, and concentrated by a rotary evaporator to provide brown lignin oil. A series of reactions using birch sawdust (18 g) afforded a solid residue (11.13 g) and lignin oil (2.93 g).

### Instrumentation

**Figure 2:**
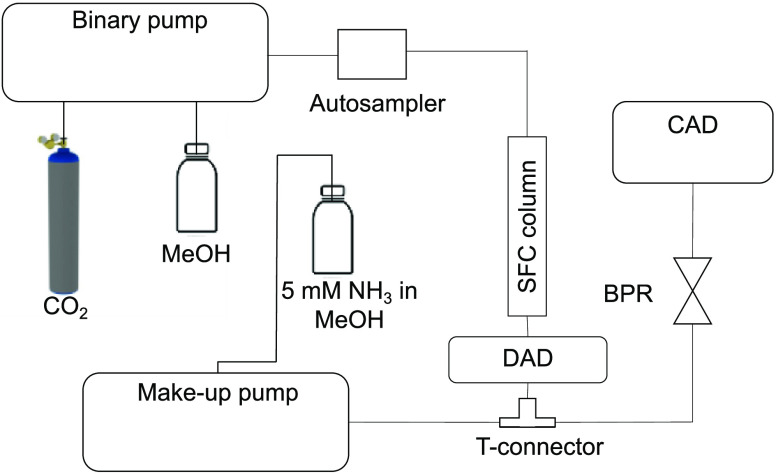
Scheme of the SFC-DAD/CAD system. MeOH: methanol; DAD: diode array detector; BPR: backpressure regulator; and CAD: charged aerosol detector.

Gel permeation chromatography was run on an Agilent 1100 LC system (Agilent Technologies, Santa Clara, CA, USA) including a G1313A autosampler, a G1311A quaternary pump, and a G1314A ultraviolet detector. SFC runs were carried out on an Agilent 1260 Infinity SFC system (Agilent Technologies, Santa Clara, CA, USA), consisting of a G4225A degasser, a G4302A binary pump, a G4303A autosampler, a G1316C thermostated column compartment, a G1315C diode array detector (DAD), a G4301A SFC controlling module, and an Agilent 1100 G1312A quaternary pump as a makeup pump, all controlled by Agilent OpenLab CDS Chem Station software. For flow splitting between the backpressure regulator (BPR) and the mass detector, a post-column adjustable flow splitter (Analytical Scientific Instruments, Richmond, CA, USA) was employed after the DAD as presented in Figure 2. CAD was carried out by an ESA Corona charged aerosol detector (ESA Biosciences), and its signal was collected through a Colibrick A/D converter by Clarity Lite software (DataApex, Praha, Czech Republic). For final peak classification by HRMS, a Waters Ultra Performance Convergence Chromatography (UPC^2^) (Waters, Milford, MA) system coupled to a Xevo G2 QToF-MS (Waters, Milford, MA) was used.

### Standards and Sample Pretreatment

All standards were weighed and dissolved in an acetone/water mixture (70:30 v/v %) with the concentration of 1–3 mg/mL. A standard mixture for method development was prepared by mixing aliquots of these stock solutions to yield 50 mg/L concentration for each dimeric compound and 400 mg/L for each monomer. Calibration solutions were prepared from the stock solutions with concentrations ranging from 15 to 125 mg/L for dimers. The concentration of monomers in the calibrators was set between 24 and 1000 mg/L, depending on the monomer (Supporting Information Table S1). The concentrations of analytes in the validation mixtures are found in Supporting InformationTable S2.

**Figure 3:**
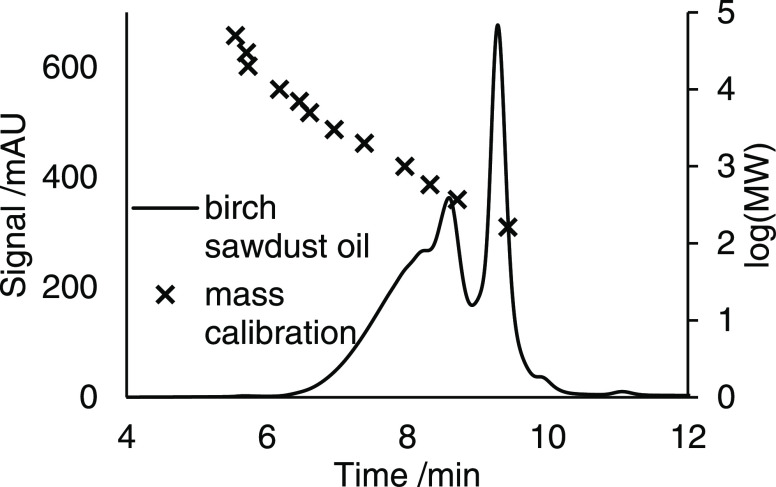
Gel permeation chromatogram of the birch sawdust oil. Column: Agilent PLGel 500 Å (300 × 7.5 mm) at 50 °C; eluent: THF (1 mL/min). Mass calibration was performed with linear polystyrene standards.

An aliquot of lignin oil sample was weighed and dissolved in THF, filtered through a polytetrafluoroethylene syringe filter (0.2 μm; VWR, Radnor, PA, USA), and diluted in THF (v/v %) to yield a final concentration of approximately 10 mg oil/mL. The oil was fractionated by means of gel permeation chromatography on an Agilent PLGel 500 Å column (300 × 7.5 mm at 50 °C; Agilent Technologies, Santa Clara, CA, USA) with THF as an eluent. Column calibration was performed using Agilent PS-L linear polystyrene standards (Agilent Technologies, Santa Clara, CA, USA). The injection volume was 10 μL. Two low-molecular-weight fractions between 8.6 and 9.2 min (“dimer fraction”) as well as between 9.2 and 9.9 min (“monomer fraction”) (see Figure 3) were collected 10 times. The collected dimer and monomer fractions were pooled, dried under nitrogen flow, and reconstituted in 100 μL of acetone/water mixture (70:30 v/v %).

### Supercritical Fluid Chromatographic Method Development

Four columns with various stationary phase ligands were evaluated in terms of chromatographic resolution. The tested columns were Torus 1-AA (1-aminoanthracene), Torus 2-PIC (2-picolylamine), Torus DIOL (high-density diol), and Torus DEA (diethylamine) (see Supporting Information Figure S1). All columns were purchased from Waters (Milford, MA, USA) with a length, inner diameter, and particle size of 100 mm, 3 mm, and 1.7 μm, respectively. Gradient programs (see Supporting Information Figure S2) were further developed to improve the resolution with the best performing column before fine-tuning by the experimental design.

A Box–Behnken experimental design^32^ was applied to fine-tune chromatography in order to maximize the chromatographic resolution between close eluting peaks. The flow rate of the mobile phase was varied between 1.2 and 1.6 mL/min, the column temperature was varied between 30 and 60 °C, and the backpressure of the system was varied between 100 and 130 bar. Furthermore, different solvents such as methanol, isopropanol, heptane, and THF were evaluated as alternative sample diluents, independently from the experimental design. MODDE (Umetrics, Umeå, Sweden) software was used to design, evaluate, and use a Box–Behnken experimental design for method optimization. Post-processing of chromatographic data was carried out using MATLAB Version R2020a (MathWorks Inc.).

### Final Chromatographic Method

The optimized chromatographic method for the separation of lignophenolics employs liquid carbon dioxide as mobile phase A and methanol as mobile phase B. Gradient elution started at 2% MeOH and kept for 1 min, increased to 12% in 2.5 min, and then further increased to 20 and 35% in 3.5 and 2.5 min, respectively. Finally, a 0.5 min-long equilibration step was employed before the next injection, yielding a total runtime of 10 min. Ammonia (5 mM) in methanol was infused post-column as a makeup solvent with a constant flow rate of 0.85 mL/min to facilitate the coupling to possible mass spectrometric detection. The best separation was achieved on a Waters Torus 1-AA (1.7 μm, 3 mm × 100 mm) column, thermostated at 30 °C while the BPR was set to 130 bars. Ultraviolet absorbance was measured at 210 nm. To ensure good sensitivity with satisfying baseline flatness, 5 pA was set for the CAD range, and the electronic filter was set to high setting.

The same separation method was directly transferred to an UPC^2^ system for ultrahigh performance SFC-MS runs. QToF-MS was operated in the positive electrospray ionization mode with a source temperature of 120 °C, a desolvation gas temperature of 400 °C, a desolvation gas flow of 450 L/h, a capillary voltage of 2.5 kV, a cone voltage of 25 V, a cone gas flow of 50 L/h, and an extractor cone voltage of 4 V. Mass spectra were recorded in the MSe mode in the range from *m*/*z* 80 to 800.

### Data Post-processing

Digital resolution enhancement was carried out by means of even-derivative peak sharpening and application of the normalized power law, as detailed here:

1. Raw data is imported into the MATLAB workspace
2. Baseline correction is applied by subtracting a variable baseline from the signal
3. Even-derivative sharpening is applied to a freely chosen segment of the chromatogram. *K*_1_ = 951 and *K*_2_ = 54 were the weights used for the calibration standards
4. The sharpened signal is normalized to the peak with the lowest height
5. The normalized signal is powered to an integer (*n* = 3 was used for standard mixtures)
6. Peak areas in the original signal are back-calculated from the powered signal according to the method by Hellinghausen et al.^33^

The evaluation of the introduced bias by the peak sharpening procedure was conducted by comparison of back-calculated concentrations of dimers from a quadratic regression on both the raw and the sharpened signal.

## Results and Discussion

### Choice of Representative Dimers

In this study, dimers that have been reported in the literature as identified in oils obtained from RCF of hardwood have been synthesized and used (compounds D1–D8).^34^ Hardwood lignins comprise both syringol (S-unit) and guaiacol (G-unit) motifs, and thus, both building blocks have been used as model compounds. RCF will generate both intact interlinkages such as 5–5 (D1), β–β (compounds D5, D6, and D7), and β-5 model (D8), as well as reduced products of the β-1 motif (D2, D3, and D4). In addition to the dimers, isolated and fully characterized monophenolic compounds M1–M6 were also synthesized and included in the study. Although we hypothesize that lignin monomers do not yield a uniform response and thus cannot be subjected to universal quantification by the CAD, they are naturally part of our sample and are considered for the method development process as controlled interferents.

### Chromatographic Method Development

Obtaining baseline separation of peaks before the charged aerosol detector is of utmost importance since this detector is assumed to be nonselective for compounds with low volatility.^35^ In our research group, several studies have been conducted on lignin analysis by SFC^24,25^ with thorough optimization of mobile and stationary phases; however, it was believed that column screening was necessary to find the stationary phase with optimal selectivity for this particular type of sample. On the other hand, the type of modifier, makeup solvent, and additives were not considered in our developmental work, but the choice was based on literature information.^26^

### Column Screening

**Figure 4:**
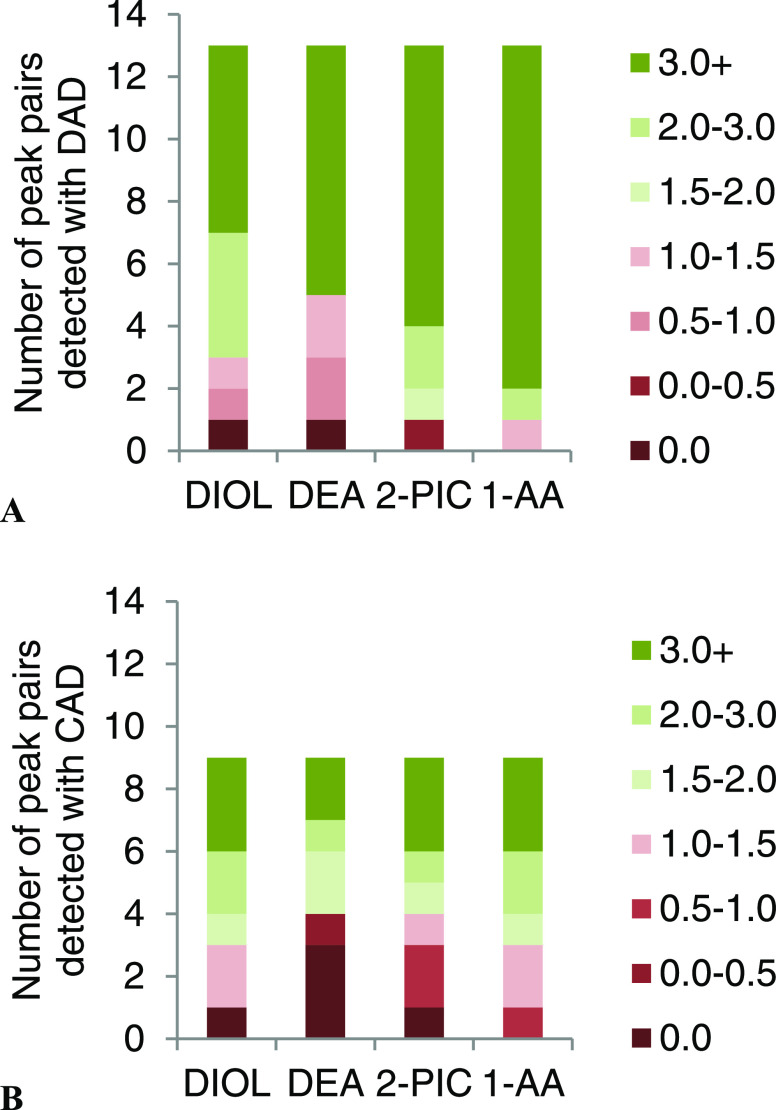
Comparison of peak resolution obtained using different column chemistries, as determined by the DAD (A) and CAD (B) signals. *R*_s_ = 0 was assigned when no shoulder was observed between two peaks. Resolutions were calculated from chromatograms with the best performing gradients for each column (see Supporting Information Figure S3 for the gradients).

Chromatographic peak resolution as evaluated by diode array detection (DAD) and CAD was calculated in order to compare the selectivity of columns with different chemistries. As illustrated in Figure 4, the 1-AA column was found to provide sufficient resolution to at least partially separate all tested compounds as detected by the CAD and separated all but one peak pair as detected by the DAD.

The underlying reason for the better selectivity is suggested to be the presence of additional π–π interactions of the tested analytes on the aminoanthracene group. This is further supported by 2-PIC being the second best performing stationary phase, similarly to what has been found previously by our research group.^25^ On the other hand, the DEA column performed the worst in terms of peak resolution, leaving three peak pairs with very poor resolution (*R*_s_ < 1) in the DAD chromatogram and two peak pairs completely coeluting in the CAD chromatogram. It can also be observed from Figure 4 that fewer analytes were detected by the charged aerosol detector since monomers without an additional alcohol function are too volatile for CAD. It should be noted that chromatographic resolution drastically decreased in the CAD compared to the DAD due to severe post-column zone broadening. This was later counterweighed by application of digital resolution enhancement techniques.

### Fine-Tuning of the Chromatographic Separation

**Figure 5:**
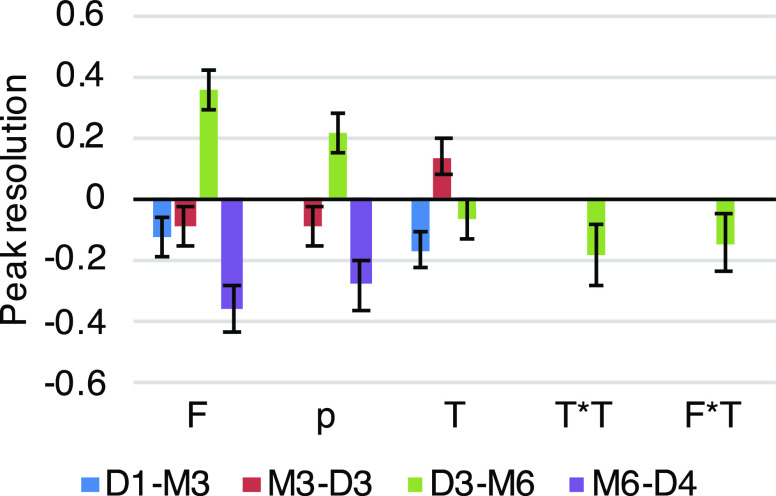
Scaled effects of variables (*F*: mobile phase flow rate; *T*: mobile phase temperature; and *p*: backpressure) on the resolution of selected peak pairs in the CAD chromatogram. Insignificant effects were removed.

As illustrated in Figure 4, three peak pairs remained incompletely separated in the CAD (*R*_s_ < 1.5) chromatogram when using the 1-AA column. In order to avoid quantification errors originating from this, a Box–Behnken experimental design with three center point replicates was run to fine-tune the method in order to improve resolutions between the four most challenging peak pairs (D1–M3, M3–D3, D3–M6, and M6–D4) in the CAD chromatogram. The flow rate and the temperature of the mobile phase, as well as the backpressure of the system, were chosen as independent variables since they were deemed to have the largest effect on resolution (Figure 5).

The effects of the variables as visualized in Figure 5 indicated that the flow rate of the mobile phase has negative effects on resolution in most cases, suggesting that lowering the flow rate yielded better resolution between peaks. Since both the temperature of the mobile phase and the system backpressure affect the density of the carbon dioxide, and thus the solvent strength of the mobile phase,^36^ a significant interaction between these two parameters was expected. However, this was not supported by the fitted model. A potential reason for this is that the investigated peak pairs elute later in the gradient, where the cosolvent ratio is higher; thus, the mobile phase is less compressible.

**Figure 6:**
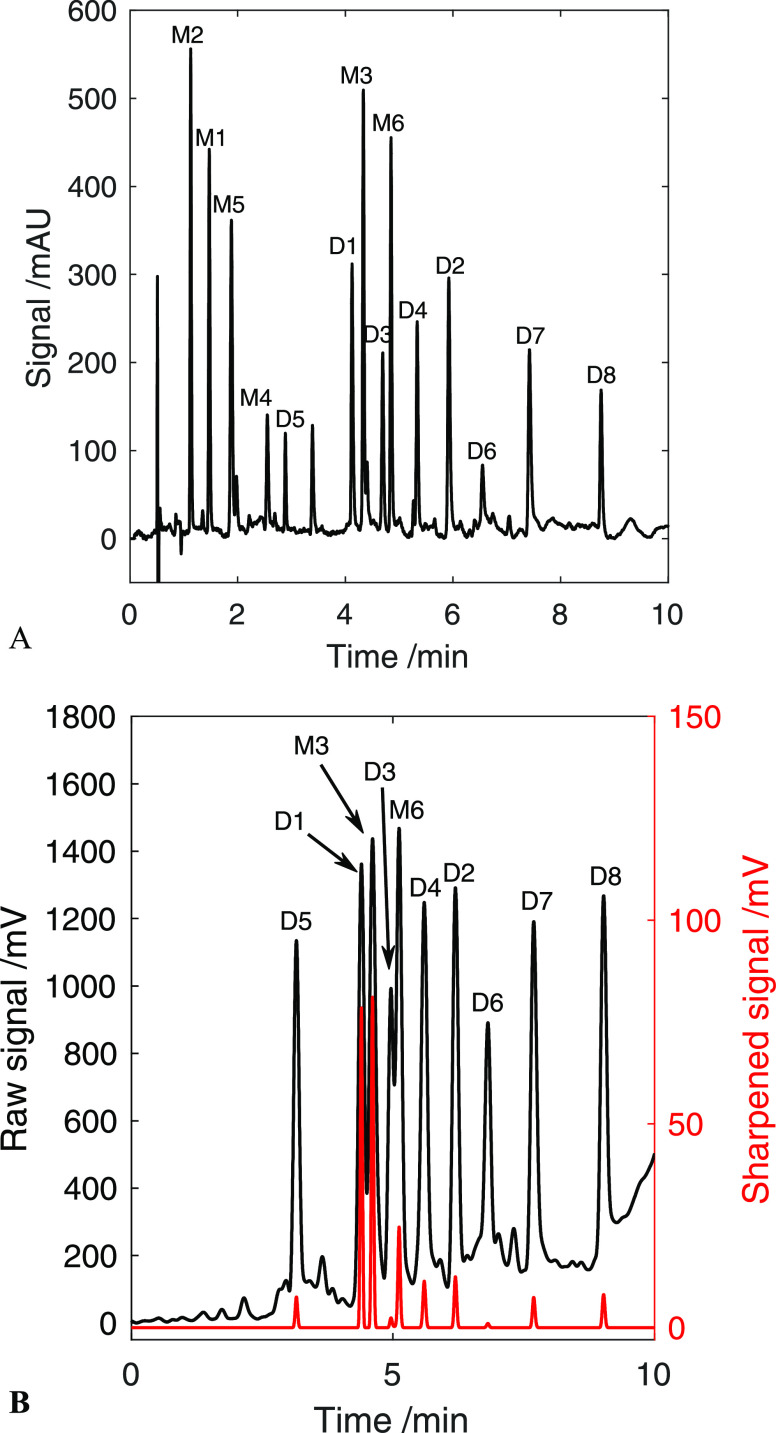
DAD at 210 nm, blank-corrected (A) and CAD (B) chromatogram of lignin phenolic standard mixture. In (B), the red chromatogram is the sharpened version of the original black one. Monomer concentrations: M2 (800 mg/L); M1 (200 mg/L); M5 (200 mg/L); M4 (800 mg/L); M3 (400 mg/L); and M6 (160 mg/L). All dimers are present at 100 mg/L concentration. Method: Torus 1-AA column (100 × 3 mm, 1.7 μm), BPR: 130 bar, column temperature: 30 °C. Mobile phase: liquid CO_2_ (solvent A) in MeOH (solvent B), 1.25 mL/min. Gradient: 0–1 min: 2% B, 1–3.5 min: 2–12% B, 3.5–7 min: 12–20% B, 7–9.5 min: 20–35% B, and 9.5–10 min: 2% B. Makeup solvent: 5 mM NH_3_ in MeOH, 0.85 mL/min.

A model was fit to the experimental data by multilinear regression and used for optimization based on the minimal normalized distance to target values. The optimal parameters were found to be 1.25 mL/min mobile phase flow rate, 30 °C column temperature, and 130 bars of backpressure (see chromatograms in Figure 6). Although it is clear that these parameters are at the very edge of the design space where the predictive power of the Box–Behnken model is relatively weak, additional runs revealed that an even lower temperature only increased the peak resolution marginally, and a lower flow rate was not favored due to increased analysis time. Additional experiments with the flow rate of the makeup solvent indicated that the peak area of most of our analytes did not improve considerably when the makeup flow rate was varied between 0.6 and 1 mL/min. Furthermore, the chromatographic resolution did not change between 0.8 and 1 mL/min (data not shown). Unpublished data from our research group concluded no significant difference in the signal-to-noise ratio of lignin-related phenolics when comparing LC/CAD and SFC/CAD as long as the makeup flow rate of the SFC setup was sufficiently high not to cause precipitation between the BPR and the CAD inlet. For these reasons, 0.85 mL/min was chosen as the makeup flow rate to ensure that no analyte loss occurs in the connecting tubing between the BPR and the CAD. Last, several alternative sample diluents were investigated to ensure the smallest possible zone broadening at injection; however, none of the tested solvents outperformed the originally selected acetone/water (70:30 v/v %) mixture in terms of peak shape and broadening. As the investigated analytes readily dissolve in acetone/water (70:30 v/v %), it is suggested that no diluent change is necessary during sample preparation.

### Assessment of the Bias of Peak Sharpening Techniques

As shown in Figure 6, the chromatographic resolution in the CAD chromatogram is considerably lower compared to that in the DAD chromatogram. This is due to the pronounced post-column zone broadening. Mathematical resolution enhancement is an established technique to increase the apparent resolution of chromatographic peaks, which has been successfully applied for ultrafast separations.^28,33,37^ During the development of the peak sharpening procedure, several aspects had to be considered to yield a useful and robust routine. First, the baseline of the raw signal must be as close as possible to zero and flat. Developing the even-derivative sharpening included empirically finding suitable weights that provide effective resolution enhancement without causing dips in the baseline. Even-derivate peak sharpening was only applied to the segment where D1, M3, and D3 were partially coeluting (peaks between 4.3 and 4.8 min in Figure 6B), although a power transform function was applied to the whole chromatogram. For the lignin oil fractions, no derivative sharpening was performed before applying the power law.

Any possible bias introduced by the peak sharpening method was evaluated by comparing the back-calculated concentrations of a standard mixture with and without peak sharpening. As Table 1 demonstrates, peak sharpening did not introduce a substantial bias in the measured peak area of lignin dimers.

**Table 1:** Back-Calculated Concentrations of Analytes in a Standard Mixture without and with Peak Sharpening (Concentration = 52.5 mg/L)

analyte	nonsharpened/mg/L	sharpened/mg/L	ratio (sharpened/nonsharpened)
D1	52.5	51.9	0.989
D2	54.2	53.8	0.992
D3	53.7	53.0	0.986
D4	56.5	55.1	0.976
D5	53.1	53.5	1.007
D6	54.4	54.2	0.996
D7	52.6	53.7	1.021
D8	54.3	54.2	0.997

### Assessment of Signal Uniformity in the Charged Aerosol Detector

While the charged aerosol detector is a theoretically universal detector, Figure 6B displays that the response of different dimers varies even though their predicted boiling points are above the arbitrary threshold of 400 °C. The difference in peak size is seemingly even higher in the sharpened signal; however, it is evident that the application of the power law causes peak areas to increase in a nonlinear manner. For this reason, back-calculated concentrations as described by Hellinghausen et al.^33^ were used in the signal uniformity investigations. Linear regression using the least-squares method was conducted in order to compare the response factors of the tested analytes. Although the nonlinear nature of the CAD has been described in the literature,^19^ the response was found to be linearly dependent on the analyte concentration in the range of 15–125 mg/L. The linear relationship was further supported by high correlation coefficients for both linear and polynomial regressions as well as the low value of the main coefficients of the quadratic regression (see Supporting Information Table S3). This range was mainly constrained by the upper output limit of the CAD as concentrations above the upper limit of the working range resulted in clipped peaks as can be seen in Supporting Information Figure S9B. For this reason, extra consideration has to be practiced when choosing dilution factors for real samples.

**Figure 7:**
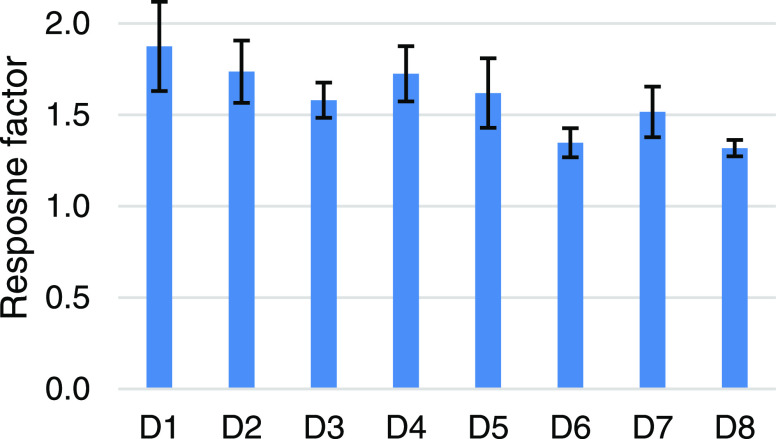
Response factors for linear calibration curves of the tested lignin dimers. Error bars represent 95% confidence intervals (*n* = 3).

The comparison of response factors is presented in Figure 7 together with the 95% confidence intervals of the slopes. According to Figure 7, the response factors of the investigated dimers range from 1.32 to 1.87 without any apparent outliers. It has to be noted that the standards were of varying purity (95 ± 5%), which might also explain a part of the inconsistency of response factors. The lack of outliers was statistically supported by a Grubbs test, which detected no outliers at α = 0.05 significance level. Although it was hypothesized that the methanol content of the mobile phase has an impact on the response factors, neither running the experiment with a countergradient nor normalizing the response factors to methanol content at the point of elution yielded more uniformity.

Aiming to address the problem at hand, that is, the scarcity of available reference standards, the option of using only one compound-specific calibration curve was evaluated. Although the similarity of response factors was confirmed, not all of the tested dimers would serve as a suitable calibrant, as shown in Table 2. The most appropriate calibrant was found by comparing the mean of the absolute values of errors of the back-calculated concentrations of a 75 mg/L standard mixture using various compound-specific calibration curves, concluding that using D2 for calibration yields the least biased quantification of the investigated dimers.

**Table 2:** Mean Errors of the Back-Calculated Concentrations of Lignin Dimeric Compounds Using the Compound-Specific Linear Calibration Curves^a^

	calibrating compound							
analyte	D1 (%)	D2 (%)	D3 (%)	D4 (%)	D5 (%)	D6 (%)	D7 (%)	D8 (%)
D1	3.3	15.3	35.3	17.2	18.9	51.8	31.5	54.6
D2	–7.7	3.4	22.3	5.3	6.2	36.5	17.9	39.0
D3	–28.2	–18.7	–2.0	–16.9	–17.5	8.0	–7.4	9.9
D4	–9.8	1.2	19.8	3.1	3.8	33.7	15.3	36.0
D5	–9.1	1.9	20.7	3.8	4.6	34.6	16.2	37.0
D6	–34.1	–25.1	–9.0	–23.3	–24.3	–0.1	–14.7	1.5
D7	–19.9	–9.8	7.8	–8.0	–7.9	19.6	2.8	21.6
D8	–34.3	–25.3	–9.2	–23.6	–24.5	–0.4	–14.9	1.2
MAE	18.3	12.6	15.8	12.7	13.5	23.1	15.1	25.1

a MAE: mean absolute error.

### Validation of the Chromatographic Method

The optimized method was validated in terms of working range, intra- and interday precision of peak areas, as well as detection limits (LODs) as presented in Table 3 and Supporting Information Table S4. Working ranges were investigated by linear regression with the least-squares method, and all analytes demonstrated an acceptable fit with *R*^2^ values above 99%. Intraday precision was calculated from the pooled relative standard deviation of the intraday standard deviations of three validation days, while the calculation of interday precision was carried out by computing the coefficient of variation between the 27 possible permutations of the three replicates on each of the three validation days and reporting the mean as interday precision for our analytes (calculations with example in Supporting Information S2). It was found that the method is sufficiently repeatable for both monomers and dimers on both low- and high-concentration levels, with all intraday RSDs being below 13% and all interday RSDs being below 17%, which are sufficiently good according to validation guidelines.^38^ LODs were obtained by diluting the standard mixtures until the signal-to-noise ratio was approximately 3 in the respective untreated signals. It should be noted that the application of the peak sharpening routine can further lower the LODs as the power law stretches out peaks from the baseline. At the same time, the application of the power law may introduce false peaks when applied to a real sample by enhancing the baseline noise if the amplitude threshold is chosen inappropriately.

**Table 3:** Validation Results of the Optimized SFC/CAD Method for Lignin Dimeric Compounds^a^

	intraday precision of peak area/% (*k* = 6)		interday precision of peak area/% (*k* = 2)				
analyte	22.5 mg/L	52.5 mg/L	22.5 mg/L	52.5 mg/L	working range/mg/L	*R*^2^	LOD/mg/L
D1	5.3	4.9	5.9	10.2	15–125	0.995	7.5
D2	6.7	4.4	16.5	16.9	15–125	0.997	7.5
D3	13.0	6.0	9.6	8.6	15–125	0.999	7.5
D4	9.5	4.8	11.9	8.9	15–125	0.998	7.5
D5	6.8	4.8	12.8	10.2	15–125	0.996	7.5
D6	7.9	4.3	13.0	11.3	15–125	0.999	7.5
D7	12.3	6.9	16.8	14.1	15–125	0.998	7.5
D8	10.2	3.6	8.1	7.1	15–125	0.998	7.5

a *k* denotes the number of degrees of freedom. Precision values are expressed as relative standard deviation.

### Classification and Quantification of Lignin Dimers in a Lignin Oil Sample

**Figure 8:**
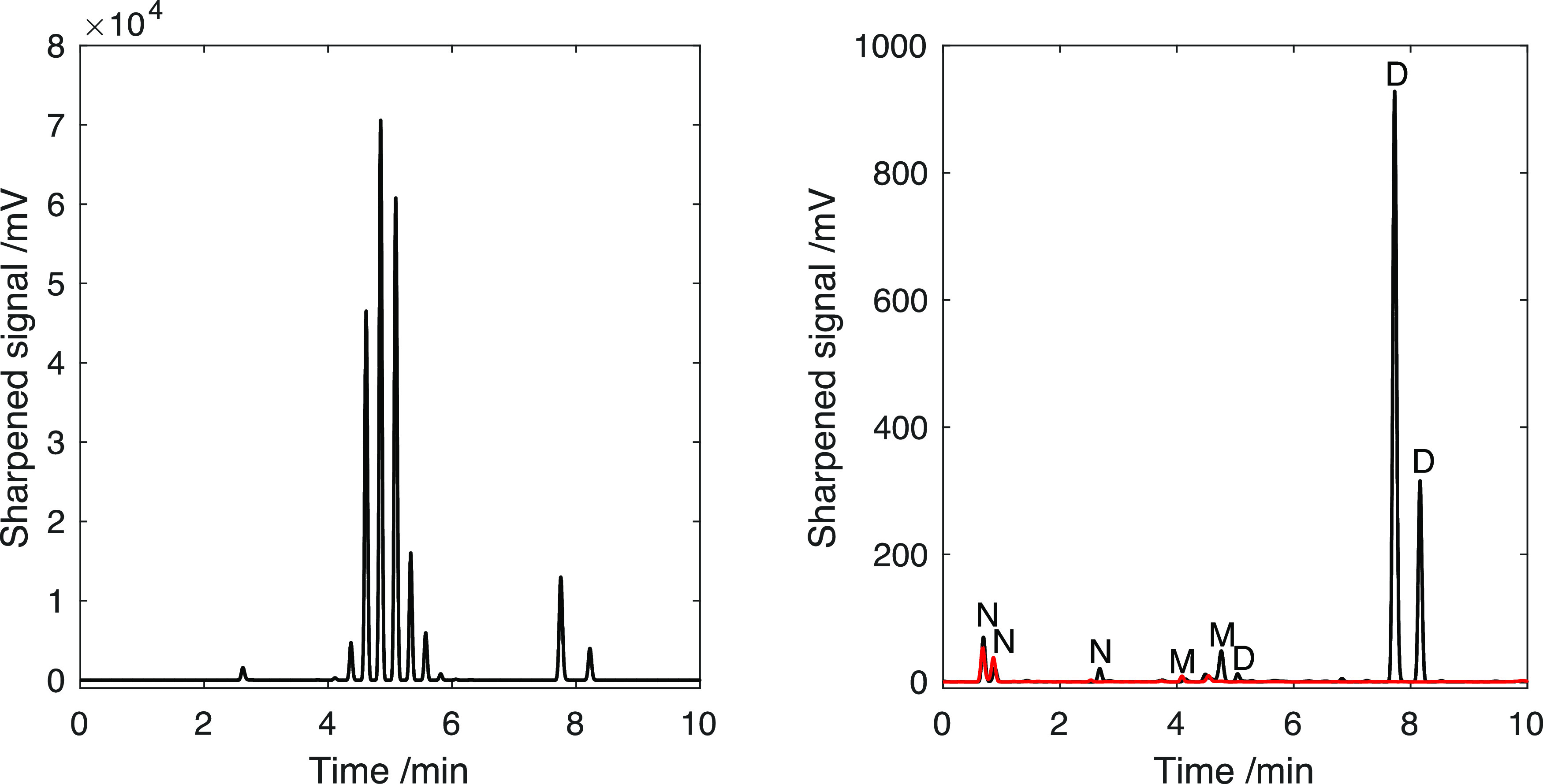
SFC/CAD chromatogram of the complete lignin oil (left), dimer fraction (right, black chromatogram), and monomer fraction (right, red chromatogram) after peak sharpening. M = monomer, D = dimer, and N = nonclassified compound.

Aiming to test the proposed one-standard calibration method on a real sample, two fractions of the birch sawdust lignin oil with average molecular weights corresponding to dimers and monomers, respectively, were separated using the developed SFC/CAD method. As presented in Figure 8, SFC/CAD chromatograms of the cleaned-up fractions contain fewer interfering peaks than that of the complete lignin oil, making digital peak sharpening easier and quantification more accurate. Furthermore, a considerable improvement in peak purity was noted as supported by the low abundance of interferent ions in Figure S8A–C. Since our approach does not require knowledge of the exact structure of the analyte but only the fact if a certain analyte is a dimer or not, a KMD-PCA-QDA classification was deemed fit for purpose instead of elucidation of the structures. This classification of peaks was carried out to select dimer peaks in the CAD signal using the strategy developed by Prothmann et al.^26^ Although quantification was not attempted, an explorative run of SFC/CAD as well as SFC/QToF-MS analysis was carried out for the dimer fraction of a spruce oil with results presented in Supporting Information Figure S9.

In order to demonstrate the applicability of using only one calibration standard to quantify dimeric compounds, the calibration curve of D2 was used for quantification of all analytes classified as dimers, as presented in Table 4. In the dimer fraction, 3 peaks were classified as dimers and quantified; besides, 11 additional peaks, which were detected in QToF-MS, were classified as dimers with concentrations below the detection limits of the CAD (Supporting Information Figure S7C). Comparison of concentrations found in the complete lignin oil and the dimer fraction did not indicate any analyte loss in the fractionation process. It is worth noting that the monomer fraction did not contain any compounds classified as lignin dimers, indicating a successful isolation of those in the corresponding fraction. Furthermore, although quantitative work was not carried out for the dimer fraction of spruce oil, by using the KMD-PCA-QDA classification model, four dimer peaks were observed in the SFC/CAD chromatogram (Supporting Information Figure S9B) with eight additional dimers which were only detected in QToF-MS due to too low concentration (Supporting Information Table S8).

**Table 4:** Detected and Quantified Dimers in the Birch Sawdust Oil Dimer Fraction (Mean ± Standard Deviation, *n* = 3)

retention time/min	oligomer class	concentration/mg/g oil
5.08	dimer	2.7 ± 0.3
7.78	dimer (D7)	11.0 ± 0.8
8.22	dimer	8.1 ± 1.1

## Conclusions

An SFC-DAD/CAD method has been developed and validated for quantification of lignin-related dimeric compounds. The DAD chromatogram shows baseline separation of all reference compounds in less than 10 min. For the CAD signal, digital resolution enhancement techniques were required to counter-weigh zone broadening after the DAD. Our hypothesis that most lignin monomers are too volatile to be detected by CAD was supported; however, it was found that monomers with an additional alcohol group can be readily detected by CAD. From the signal uniformity investigations, it was concluded that the response factors of the tested dimers did not differ significantly from one another, allowing the proposal of one single compound yielding the smallest quantification error as a calibrant. A major challenge is the relatively low sensitivity of the charged aerosol detector, which suggests that the developed quantification strategy is best suited for lignin dimers and oligomers at ppm levels of concentrations and above. It was also inferred that the developed method is applicable for lignin samples regardless of the linkage motif or the number of methoxy groups on the backbone structure. Furthermore, the potential of the universal calibration approach has been demonstrated by applying it to a complex RCF oil sample. As can be seen, this approach requires high peak-to-peak resolution, which is challenging in the case of such a complex sample, prompting the use of additional sample preparation techniques, for instance, gel permeation chromatography. With further developments of the method aimed toward online coupling of the two separation modes, our method offers rapid, derivatization-free quantification of both monomers and dimers from the RCF process and has the potential to become a sensible alternative for the more widespread GC-FID methods.

## Supporting Information Available

Synthesis schemes, NMR spectra of all compounds synthesized in this study, concentrations of standard mixtures used during method development and validation, mobile-phase gradient profiles during method development of the SFC separation, chemical structures of stationary phases tested in SFC, a coefficient plot in the design of experiment for SFC optimization, regression coefficients of lignin dimers in DAD, CAD, and FID; calibration curves of lignin dimers in the CAD; chromatograms of the dimer fraction of lignin oil by SFC-DAD and SFC/QToF-MS; variables and score plots of the KMD-PCA-QDA classification model; HRMS spectra of classified and quantified dimer peaks in the dimer fraction of lignin oil as well as chromatograms of the dimer fraction of spruce oil together with the respective KMD-PCA-QDA score plot (PDF)
