# Supplementary material for: Single-Standard Quantification Strategy for Lignin Dimers by Supercritical Fluid Chromatography with Charged Aerosol Detection
Source: Anal Chem. 2022 Dec 22;95(2):1436–45. doi: 10.1021/acs.analchem.2c04383 (PMC9850414; doi:10.1021/acs.analchem.2c04383)
Supplement: Supplementary file 1 — ac2c04383_si_001.pdf [file ac2c04383_si_001.pdf]

Supporting Information: A single-standard quantification strategy for lignin dimers by supercritical fluid chromatography with charged aerosol detection

Daniel Papp[†], Thanya Rukkijakan[‡], Daria Lebedeva[‡], Tommy Nylander[§], Margareta Sandahl[†], Joseph S. M. Samec[*, ‡], Charlotta Turner[*,†]

†Lund University, Department of Chemistry, Centre for Analysis and Synthesis, P.O. Box 124, SE-22100 Lund, Sweden

‡Stockholm University, Department of Organic Chemistry, Svante Arrhenius väg 16C, SE-106 91, Stockholm, Sweden

§Lund University, Department of Chemistry, Physical Chemistry, P.O. Box 124, SE-22100 Lund, Sweden

Table of Contents

S1: Preparation of lignin model compounds S-2

Table S1: Calibration ranges of analytes. Concentrations are presented in mg/L units S-21

Table S2: Concentration levels of standard mixtures for precision and bias measurements presented as mg/L S-22

Figure S1: Chemical structure of stationary phases used during the chromatographic method development S-23

Figure S2: Gradient profiles tested with the 1-AA column during method development S-24

Figure S3: Best performing gradients with the tested columns during the SFC method development S-25

Figure S4: Scaled effects of variables of the experimental design S-26

Table S3: Regression coefficients of the compound-specific calibration curves of lignin dimers in the DAD, CAD and FID S-27

S2: Detailed calculation workflow with example for the calculation of interday precision S-28

Table S4: Validation results of the optimized SFC method for lignin monomers as detected by the DAD at 210 nm S-29

Figure S5: Compound specific calibration curves of lignin dimers in the charged aerosol detector S-30

Figure S6: SFC-DAD (A) and SFC/QToF-MS (B) chromatograms of the dimer fraction of the birch oil S-31

Table S5: Variables used for KMD-PCA-QDA classification of the detected peaks in the complete birch oil S-33

Table S6: Variables used for KMD-PCA-QDA classification of the detected peaks in the monomer fraction of the birch oil S-39

Table S7: Variables used for KMD-PCA-QDA classification of the detected peaks in the dimer fraction of the birch oil S-41

Figure S7: Score plots of the KMD-PCA-QDA classification model of dimers. Overlaid green stars in figures A, B and C denote the detected peaks in the unfractionated birch oil, the as well as the monomer and the dimer fractions of the birch oil, respectively S-43

Figure S8: ESI-QToF mass spectra of the three dimers quantified by the CAD. Mass spectra in A, B and C belong to dimers detected at 5.08 min, 7.78 min and 8.22 min by the CAD, respectively S-46

Figure S9: SFC-DAD (A), SFC/CAD (B) chromatograms and score plot of the KMD-PCA-QDA dimer classification (C) of the dimer fraction of the spruce oil S-49

Table S8: Variables used for KMD-PCA-QDA classification of the detected peaks in the dimer fraction of the spruce oil S-52

References S-54

S1: Preparation of lignin model compounds

Synthesis of (E)-2,6-dimethoxy-4-(prop-1-en-1-yl)phenol (M4)

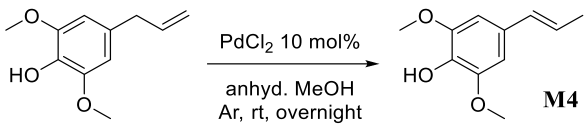

Compound M4 was prepared following the adapted procedure[1]. Commercially available 4-allyl-2,6-dimethoxyphenol (1.9 g, 10 mmol) was dissolved with anhyd. methanol (MeOH) under argon atmosphere followed by addition of $PdCl_2$ (0.18 g, 1 mmol) at room temperature. After the reaction was stirred and left overnight, the mixture was filtered through Celite® and concentrated by rotary evaporator. The oily crude product was purified by flash column chromatography (SiO_2, 10-20% Ethyl acetate (EtOAc)/ Petroleum ether (PE) to provide M4 as a yellow oil (1.5 g, 79%). The spectral data were in accordance with the previous report[1].

^{1}H NMR (400 MHz, $CDCl_3$) δ 6.57 (s, 2H), 6.31 (dd, *J* = 15.6, 1.7 Hz, 1H), 6.09 (dq, *J* = 15.6, 6.6 Hz, 1H), 5.45 (s, 1H), 3.89 (s, 6H), 1.86 (dd, *J* = 6.6, 1.6 Hz, 3H).

^{13}C NMR (101 MHz, $CDCl_3$) δ 147.2, 134.1, 131.1, 129.7, 124.0, 102.7, 56.4, 18.5.

HRMS (ESI$^+$) m/z:[M + Na]$^+$calcd. for $C_{11}H_{14}NaO_3$ 217.0835; found, 217.0833;

^{1}H NMR:

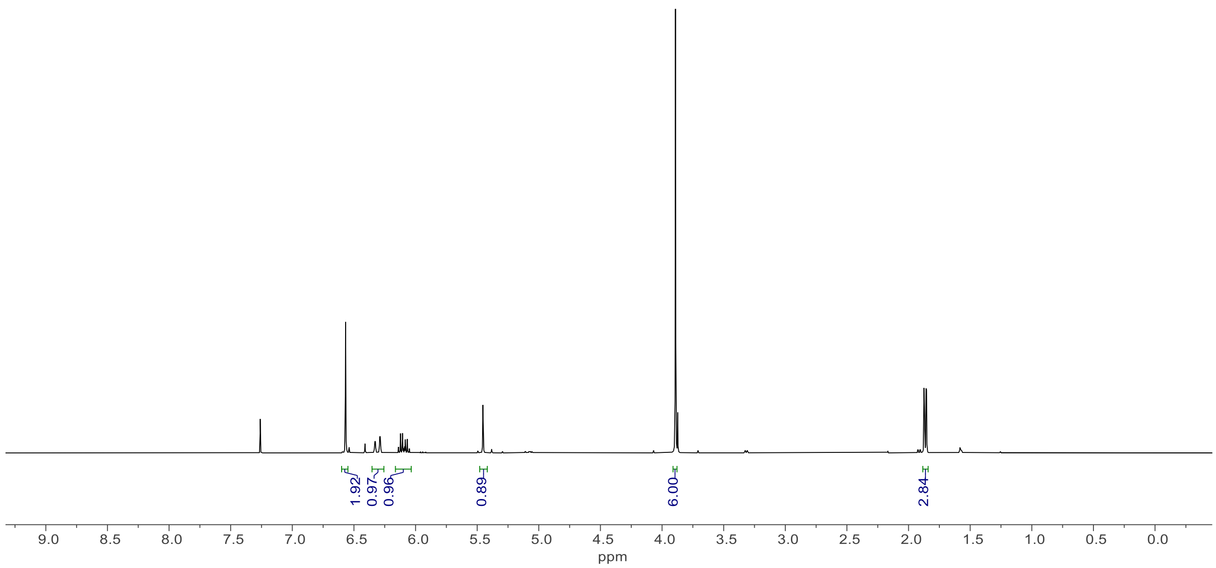

^{13}C NMR:

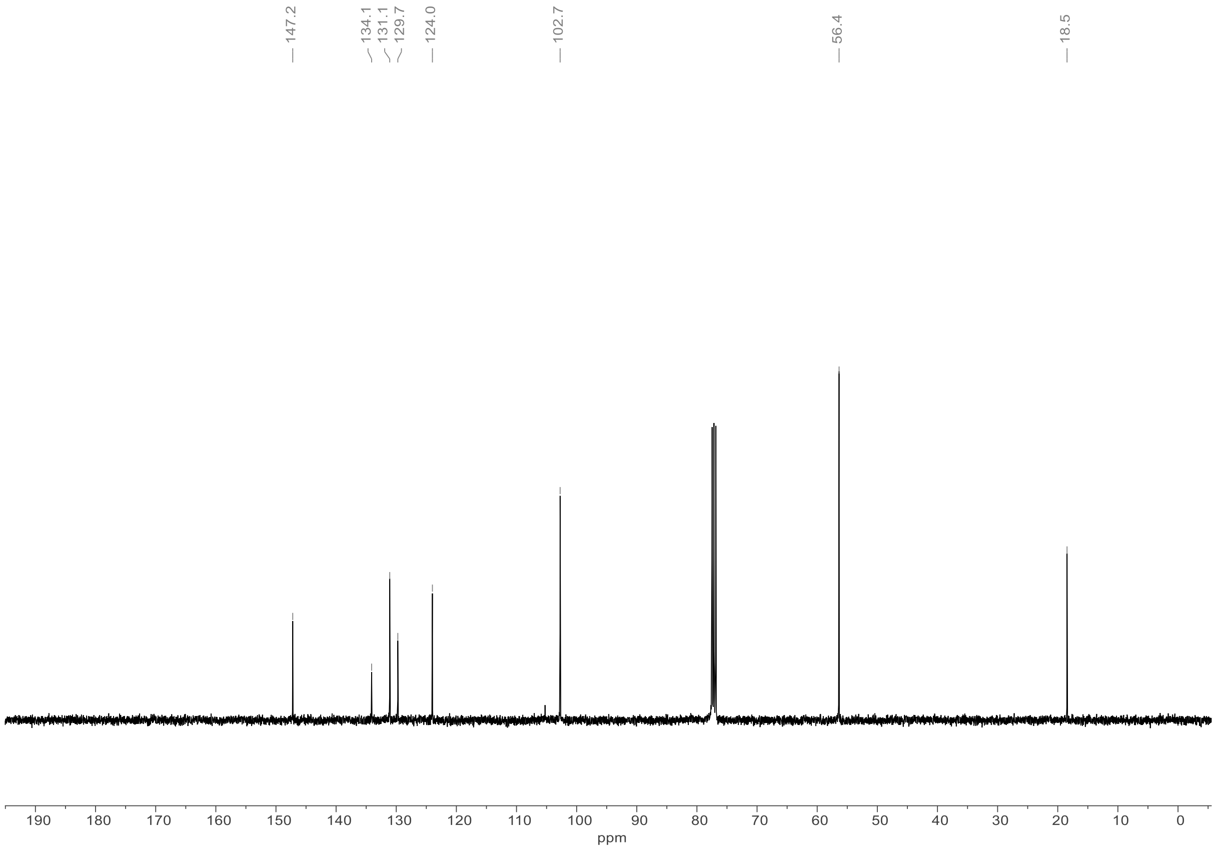

Synthesis of 2,6-dimethoxy-4-propylphenol (M5)

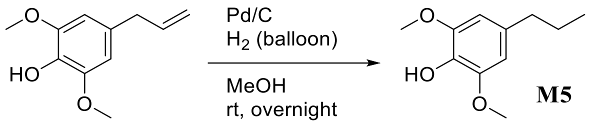

M5

4-allyl-2,6-dimethoxyphenol (1 g, 5 mmol) dissolved in MeOH (20 mL) with Pd/C (0.1 g, 5wt% of Pd) was stirred under H_2 atmosphere by balloon overnight. The mixture was filtered by syringe filtered through Celite® and concentrated by rotary evaporator. The crude product was purified by short flash column chromatography (SiO_2, 20% EtOAc/PE) to give M5 as a colorless oil (0.74 g, 76%). The spectral data were in accordance with the previous report[2].

^{1}H NMR (400 MHz, $CDCl_3$) δ 6.40 (s, 2H), 5.35 (s, 1H), 3.87 (s, 6H), 2.57 – 2.45 (m, 2H), 1.63 (h, J = 7.4 Hz, 2H), 0.94 (t, J = 7.3 Hz, 3H).

^{13}C NMR (101 MHz, $CDCl_3$) δ 147.0, 134.0, 132.8, 105.1, 56.4, 38.4, 25.0, 13.9.

HRMS (ESI$^+$) m/z:[M + Na]$^+$calcd. for $C_{11}H_{16}NaO_3$ 219.0992; found, 219,0999;

^{1}H NMR:

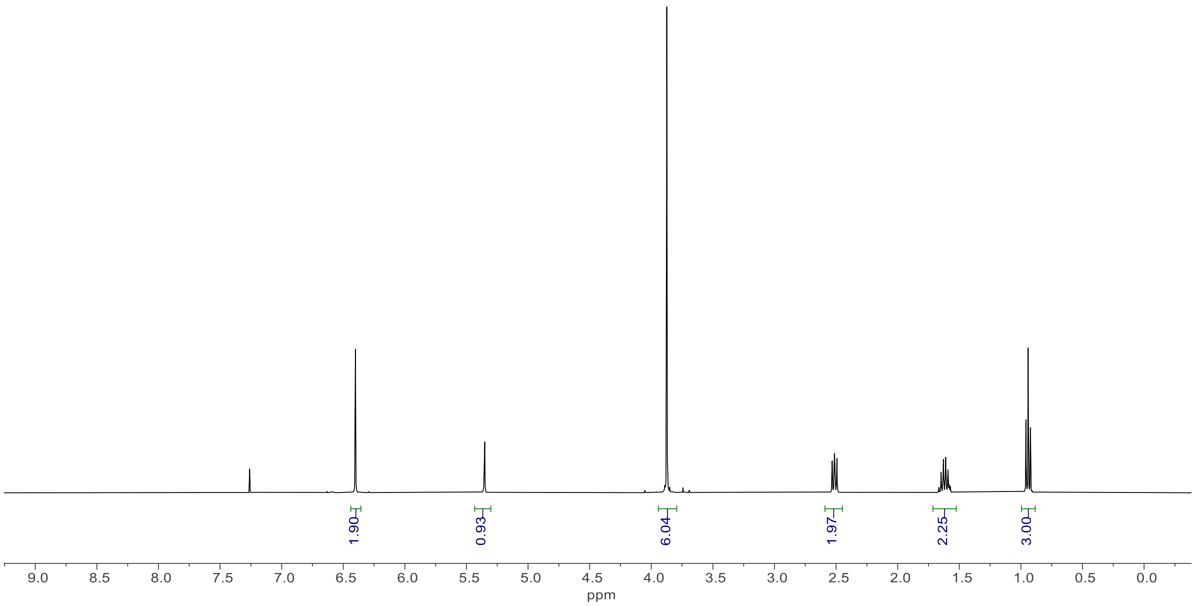

^{13}C NMR:

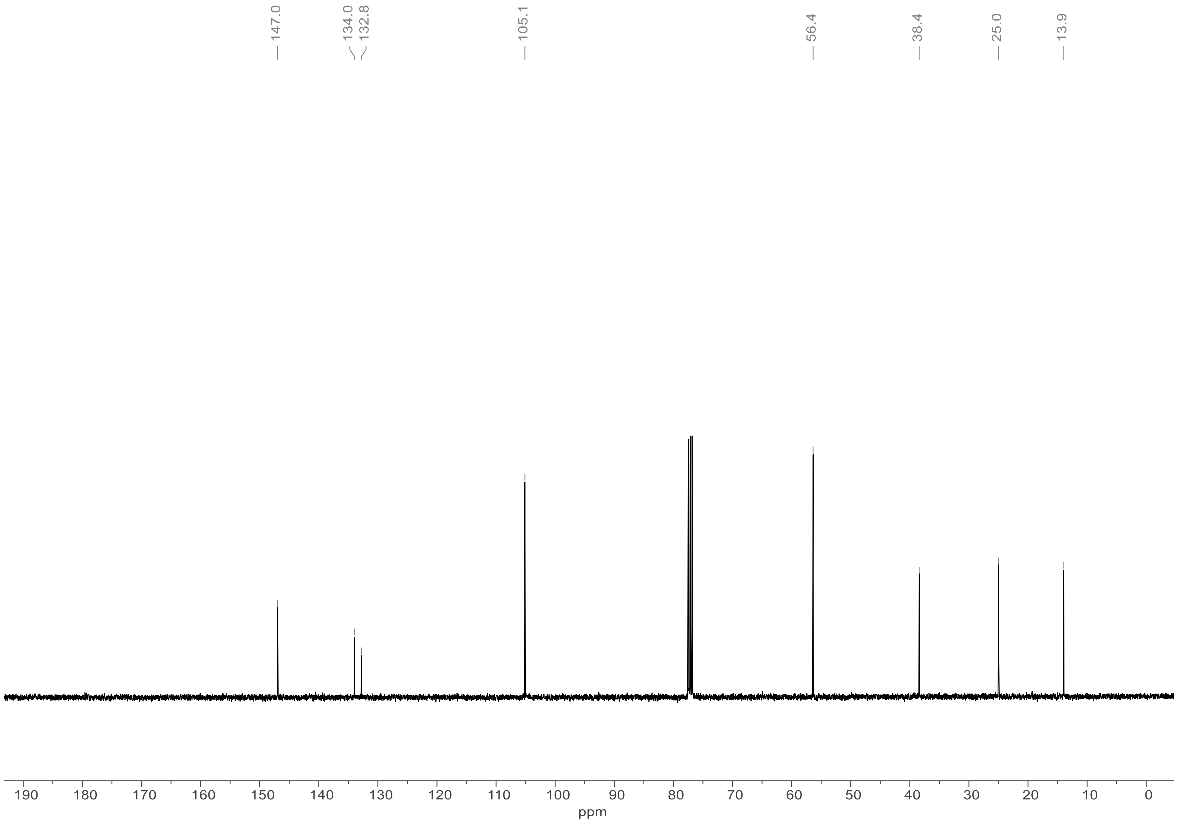

Synthesis 4-(3-hydroxypropyl)-2,6-dimethoxyphenol (M6)

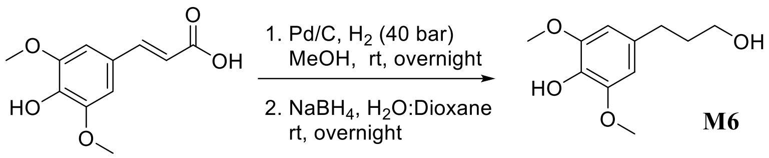

M6

Compound M6 was synthesized via 2 sequential steps following the procedure from Klein *et al*.[3]. Commercially available 3,5-dimethoxy-4-hydroxycinnamic acid (0.8 g, 3.6 mmol) was dissolved in MeOH (10 mL) and transferred to a stainless steel reactor with containing Pd/C (0.1 g, 5wt% of Pd). The reactor was pressured with H_2 (40 bar) and stirred at 200 °C overnight. After cooling the reactor to ambient temperature, the pressure was released carefully. The reaction mixture was filtered with Celite and concentrated by rotary evaporator. The crude intermediate, methyl 3-(4-hydroxy-3,5-dimethoxyphenyl)propanoate (0.2 g, 1 mmol) was used next step without purification.

^{1}H NMR (400 MHz, $CDCl_3$) δ 6.42 (s, 2H), 3.86 (s, 6H), 3.67 (s, 3H), 2.87 (t, *J* = 7.8 Hz, 2H), 2.60 (t, *J* = 7.8 Hz, 2H).

The intermediate was dissolved by H_2O/Dioxane (1:1, 10 mL) followed by addition of $NaBH_4$ (0.38 g, 10 mmol) in small portion. The reaction mixture was stirred overnight at room temperature, then quenched by addition of 0.1 M HCl (10 mL) and extracted by EtOAc (5 x 10 mL). The organic layers were combined, washed with water (10) and concentrated by rotary evaporator. The product M6 was obtained after purification by flash column chromatography (SiO_2, 10-20% EtOAc/PE) as a colorless oil (0.18 g, 24% in 2 steps). The spectral data were in accordance with the report[3].

^{1}H NMR (400 MHz, $CDCl_3$) δ 6.42 (s, 2H), 3.86 (s, 6H), 3.68 (t, *J* = 6.4 Hz, 2H), 2.71 – 2.56 (m, 2H), 1.98 – 1.80 (m, 2H).

^{13}C NMR (101 MHz, $CDCl_3$) δ 147.1, 133.0, 132.9, 105.1, 62.4, 56.4, 34.6, 32.4.

HRMS (ESI$^+$) m/z:[M + Na]$^+$calcd. for $C_{11}H_{16}NaO_3$ 235.0941; found, 235.0940;

^{1}H NMR:

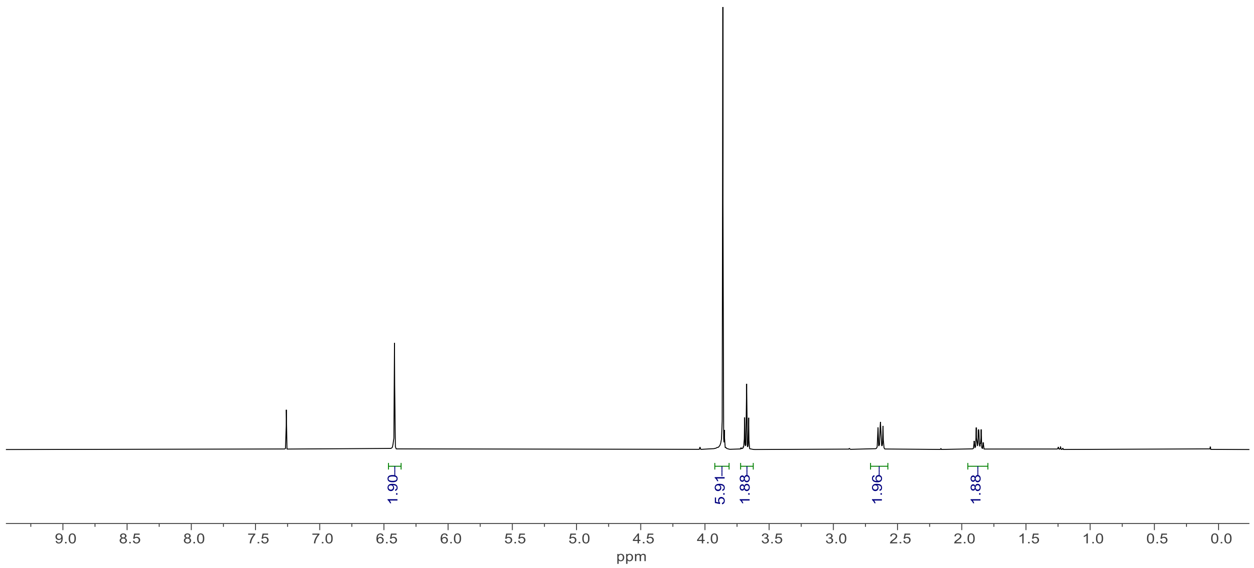

^{13}C NMR:

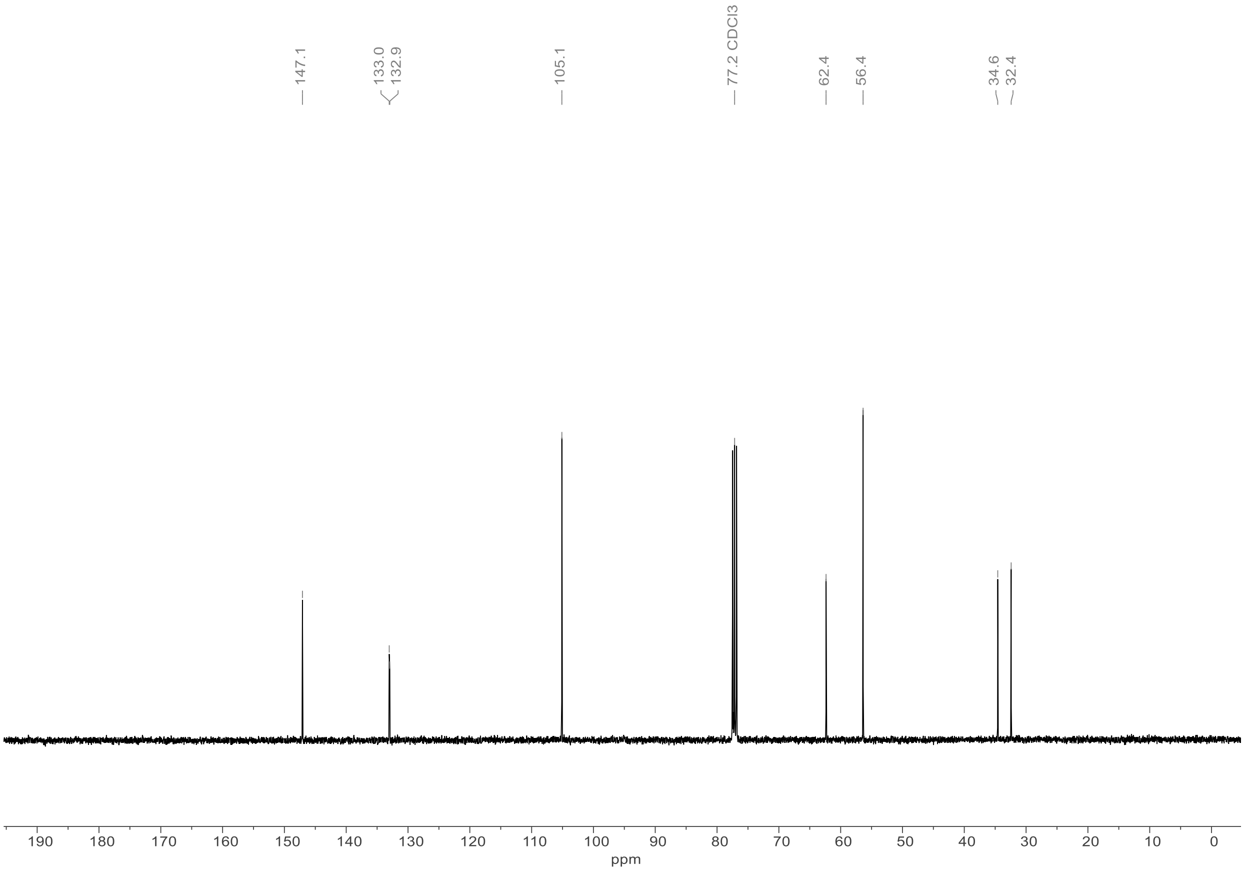

Synthesis of 3,3'-dimethoxy-5,5'-dipropyl-[1,1'-biphenyl]-2,2'-diol (D1)

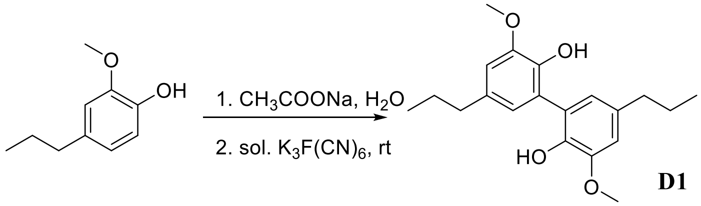

D1

Compound D1 was synthesized according to the published procedure by Subbotina[4] in 42%. The compound was analysed by ^{1}H NMR, ^{13}C NMR and HRMS.

^{1}H NMR (400 MHz, $CDCl_3$) δ 6.86 – 6.65 (m, 4H), 6.03 (s, 2H), 3.92 (s, 6H), 2.63 – 2.50 (m, 4H), 1.65 (h, *J* = 7.4 Hz, 4H), 0.96 (t, *J* = 7.3 Hz, 6H).

^{13}C NMR (101 MHz, $CDCl_3$) δ 147.3, 140.6, 134.8, 124.6, 123.1, 110.8, 56.2, 38.0, 24.9, 14.0.

HRMS (ESI^{+}) m/z:[M + Na]$^{+}$calcd. for $C_{20}H_{26}NaO_4$ 353.1723; found, 353.1723;

^{1}H NMR:

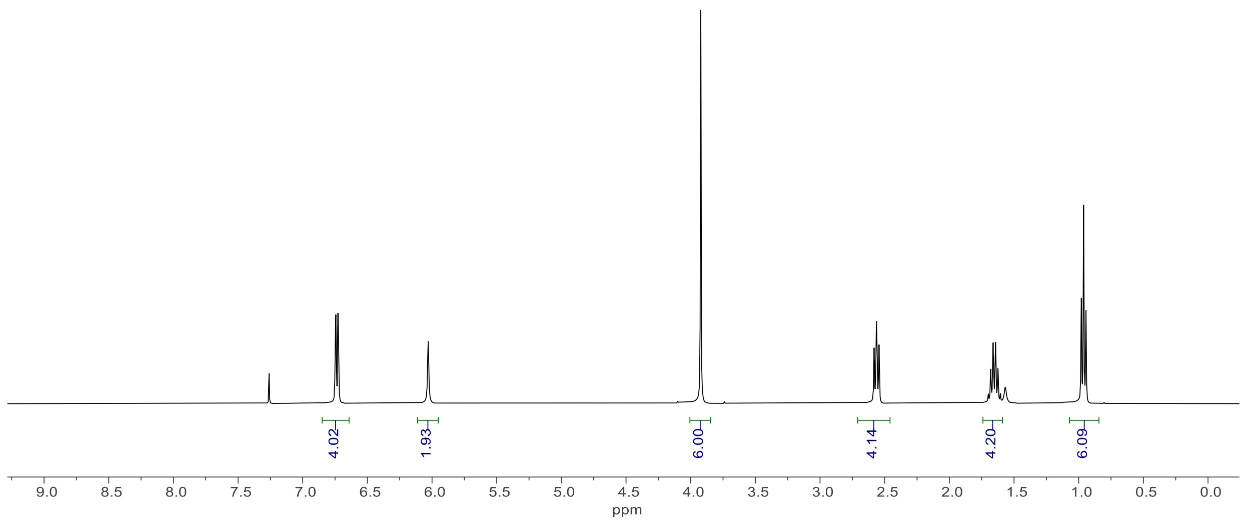

^{13}C NMR:

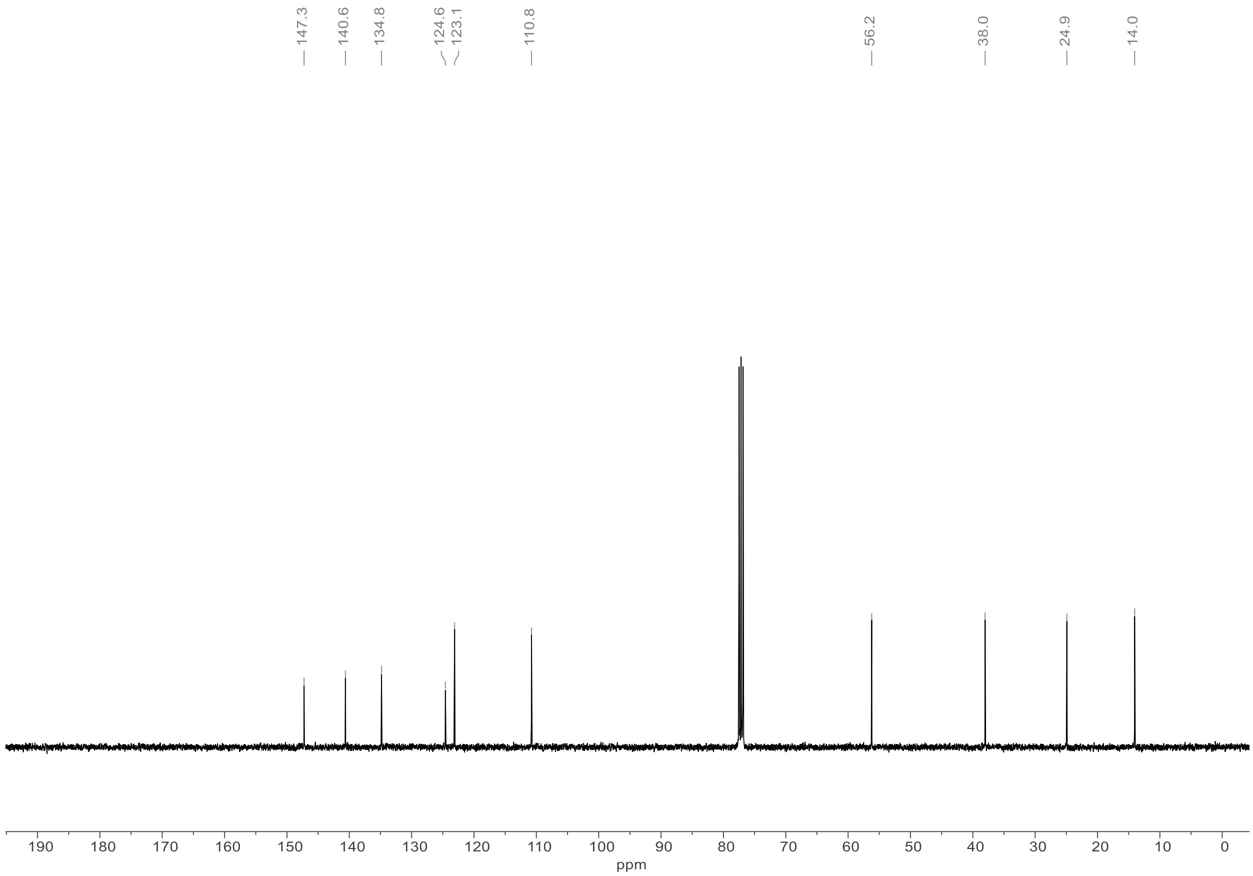

Olefin metathesis for preparation of dimeric lignin model compounds D2 to D4

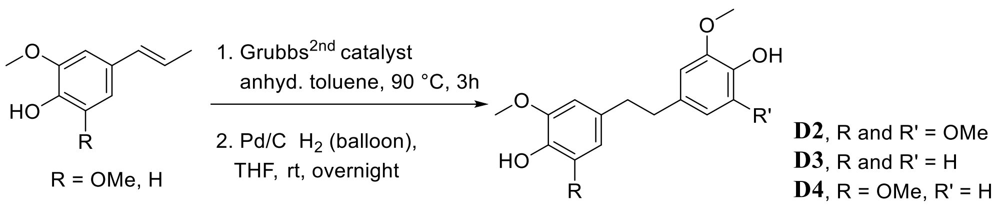

Compound D2 was prepared via two steps sequentially, olefin metathesis and reduction following the modified procedure from Ferré *et al.*[5]. The olefin metathesis was performed with the compound M4 (1 g, 5 mmol) was dissolved in anhyd. toluene (10 mL) under argon atmosphere. Grubbs 2nd generation catalyst (5 mg, 0.1 mol%) was added to the reaction mixture. The reaction was stirred at 90 °C for 3 hours, then toluene was removed under vacuum to obtain a crude intermediate used in the next step immediately.

The crude intermediated from previous step dissolved in THF (20 mL) with 5% Pd/C (150 mg) was stirred vigorously under H_2 atmosphere (balloon) overnight. The mixture was filtered through Celite® and concentrated by rotary evaporator. The crude product was purified by column chromatography (SiO_2, 20-50% EtOAc/PE) to give D2 as a white solid (0.5 g, 61%). The spectral data were in accordance with the previous report[6].

4,4'-(ethane-1,2-diyl)bis(2,6-dimethoxyphenol) (D2)

^{1}H NMR (400 MHz, $CDCl_3$) δ 6.36 (s, 4H), 5.37 (s, 2H), 3.85 (s, 12H), 2.81 (s, 4H).

^{13}C NMR (101 MHz, $CDCl_3$): δ 147.0, 133.0, 132.9, 105.3, 56.4, 38.6.

HRMS (ESI$^+$) *m/z*: $[M + Na]^+$ calcd. for $C_{18}H_{22}NaO_6$ 357.1309; found, 357.1309.

^{1}H NMR:

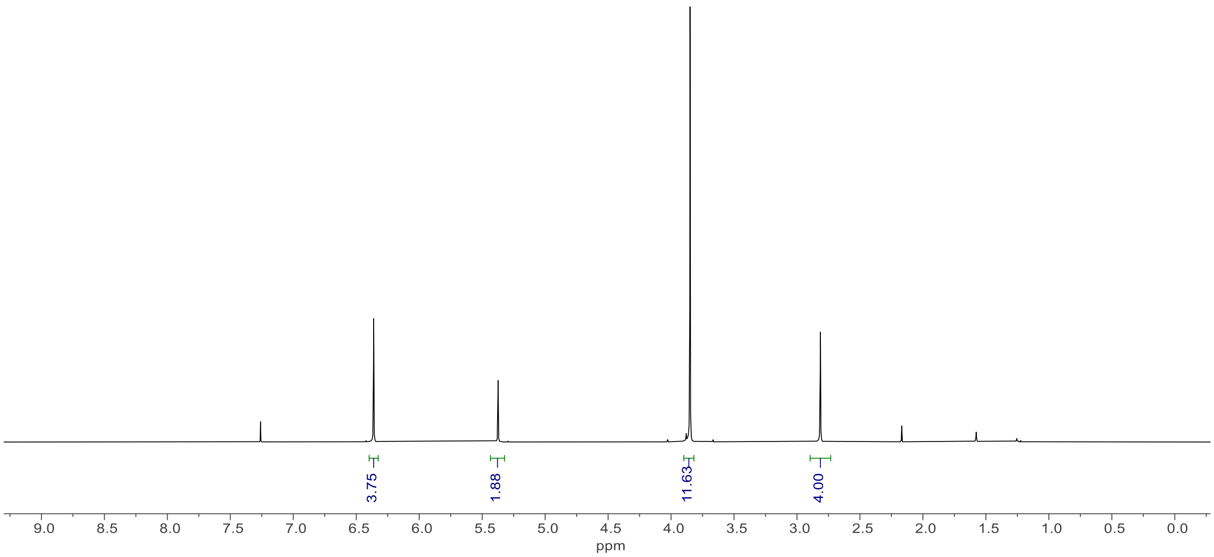

^{13}C NMR:

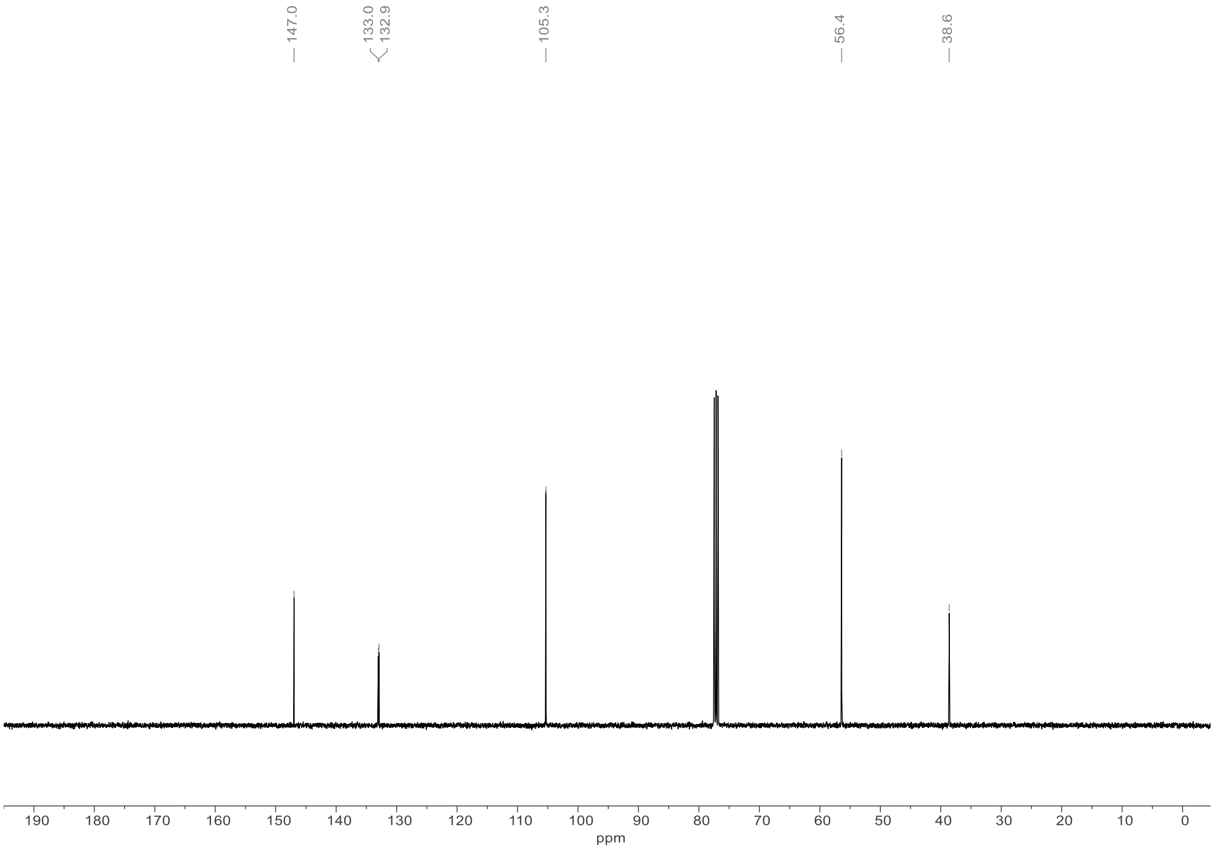

The olefin cross metathesis for synthesis of compound D3 and D4 was performed with the same method as D2. Mixture of compound M1 (4 g, 25 mmol) and M4 (0.5 g, 2.6 mmol) was dissolved in anhyd. toluene (20 mL) and stirred at 90 °C. Grubbs 2nd generation catalyst (5 mg, 0.1 mol%) was added to the reaction mixture. After 3 hours, toluene was removed under vacuum to provide the crude intermediate. The reduction of the intermediate was performed as similar method as reported with D2 using Pd/C (300 mg, 5wt% of Pd). The spectral data of D3 and D4 were in accordance with the previous report[6].

4,4'-(Ethane-1,2-diyl)bis(2-methoxyphenol) (D3): white solid (2.2g, 73% from M4)

^{1}H NMR (400 MHz, $CDCl_3$) δ 6.84 (d, *J* = 8.0 Hz, 2H), 6.68 (dd, *J* = 8.1, 2.0 Hz, 2H), 6.61 (d, *J* = 2.0 Hz, 2H), 5.49 (s, 2H), 3.84 (s, 6H), 2.82 (s, 4H).

^{13}C NMR (101 MHz, $CDCl_3$) δ 146.4, 143.9, 133.9, 121.2, 114.3, 111.4, 56.0, 38.1.

HRMS (ESI$^+$) *m/z*: $[M + Na]^+$ calcd. for $C_{18}H_{22}NaO_6$ 297.1097; found, 297.1097.

^{1}H NMR:

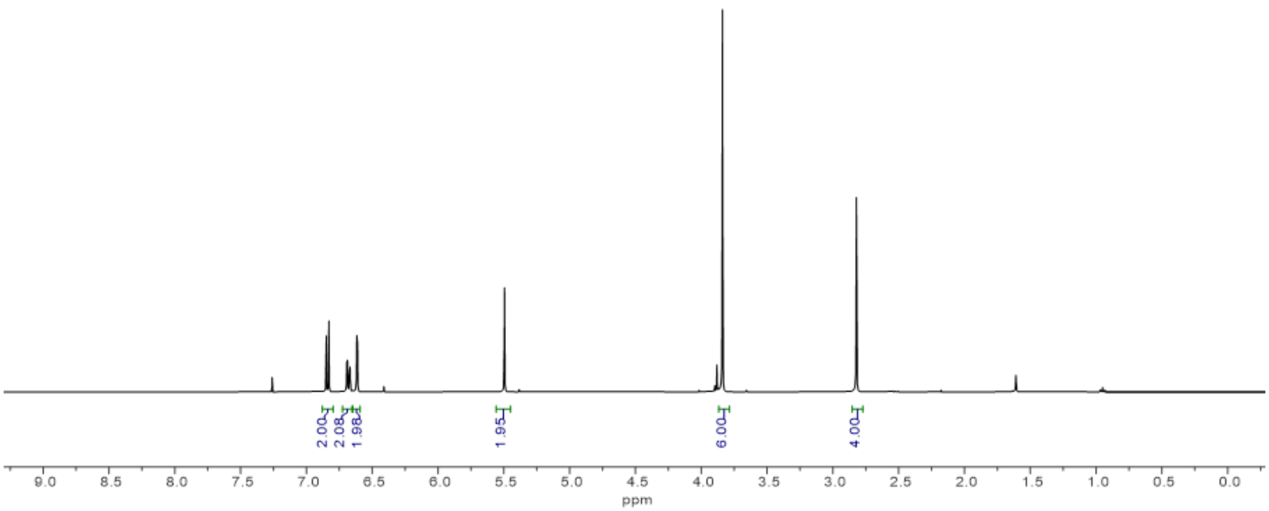

^{13}C NMR:

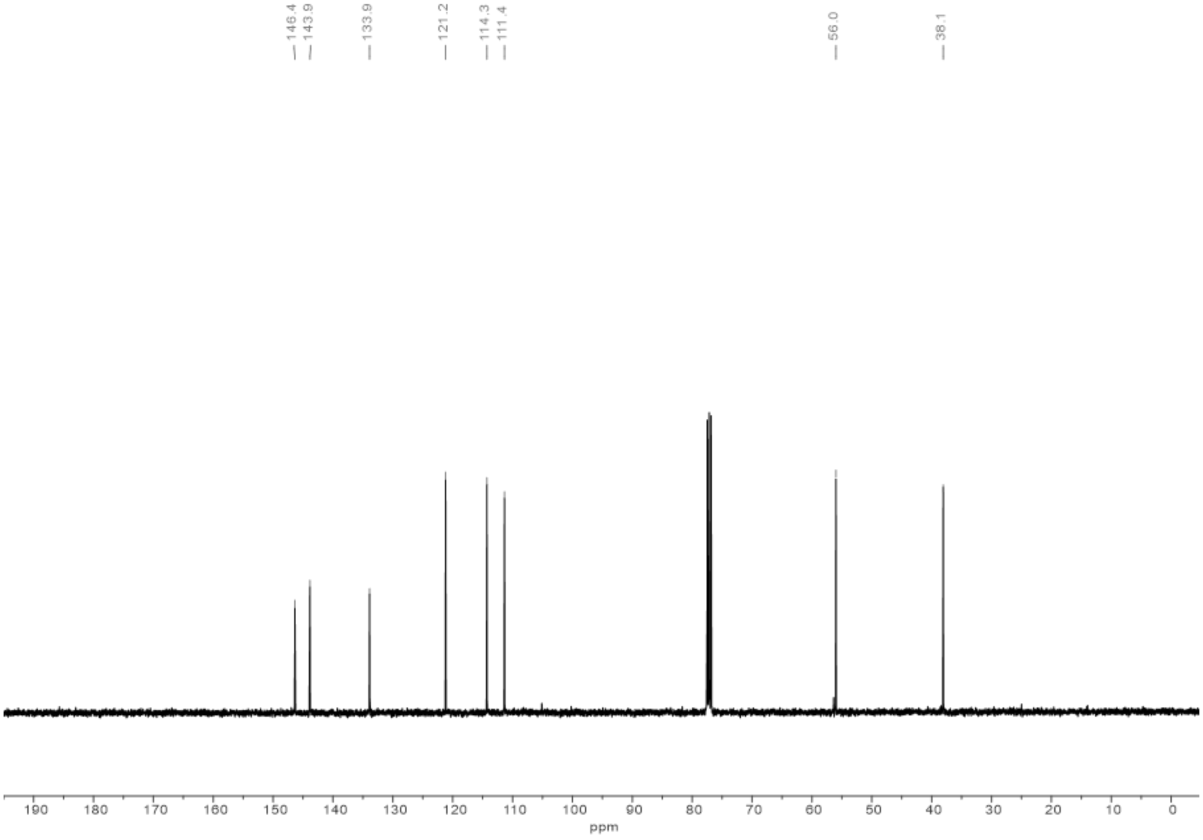

4-(4-hydroxy-3-methoxyphenethyl)-2,6-dimethoxyphenol (D4): as a white solid (0.26 g, 34% from M1)

^{1}H NMR (400 MHz, $CDCl_3$) δ 6.84 (d, *J* = 8.0 Hz, 1H), 6.68 (dd, *J* = 8.0, 2.0 Hz, 1H), 6.61 (d, *J* = 1.9 Hz, 1H), 6.36 (s, 2H), 5.51 – 5.45 (m, 1H), 5.41 – 5.35 (m, 1H), 3.85 (s, 6H), 3.84 (s, 3H), 2.81 (s, 4H).

^{13}C NMR (101 MHz, $CDCl_3$) δ 147.0, 146.4, 143.9, 133.8, 133.0, 133.0, 121.2, 114.3, 111.4, 105.3, 56.4, 56.0, 38.6, 38.1.

HRMS (ESI^{+}) *m/z*: [M + Na]$^{+}$ calcd. for $C_{18}H_{22}NaO_6$ 327.1203; found, 327.1208.

^{1}H NMR:

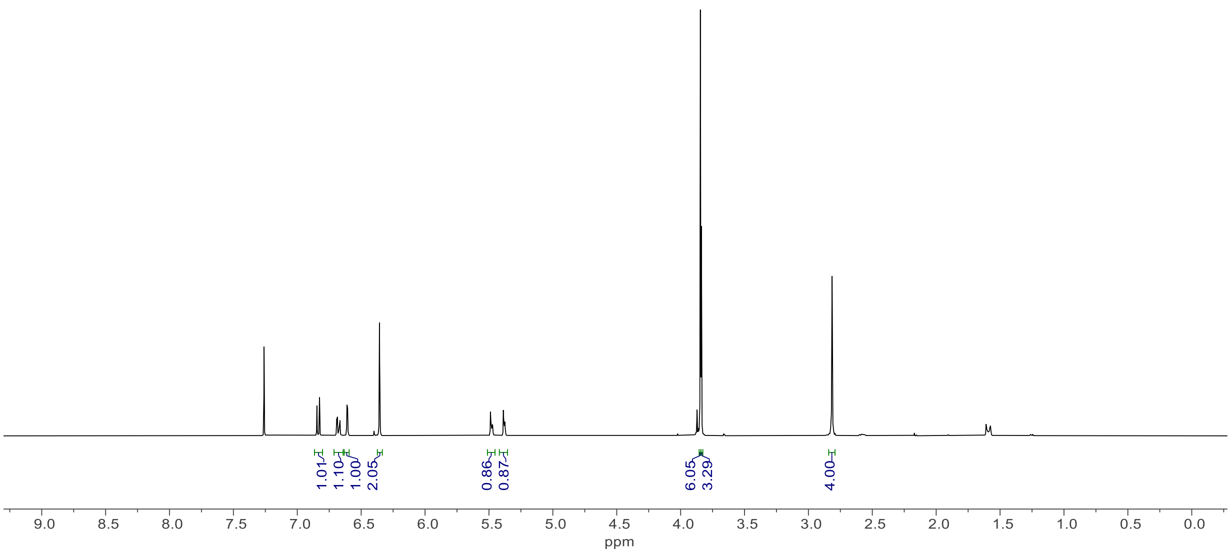

^{13}C NMR:

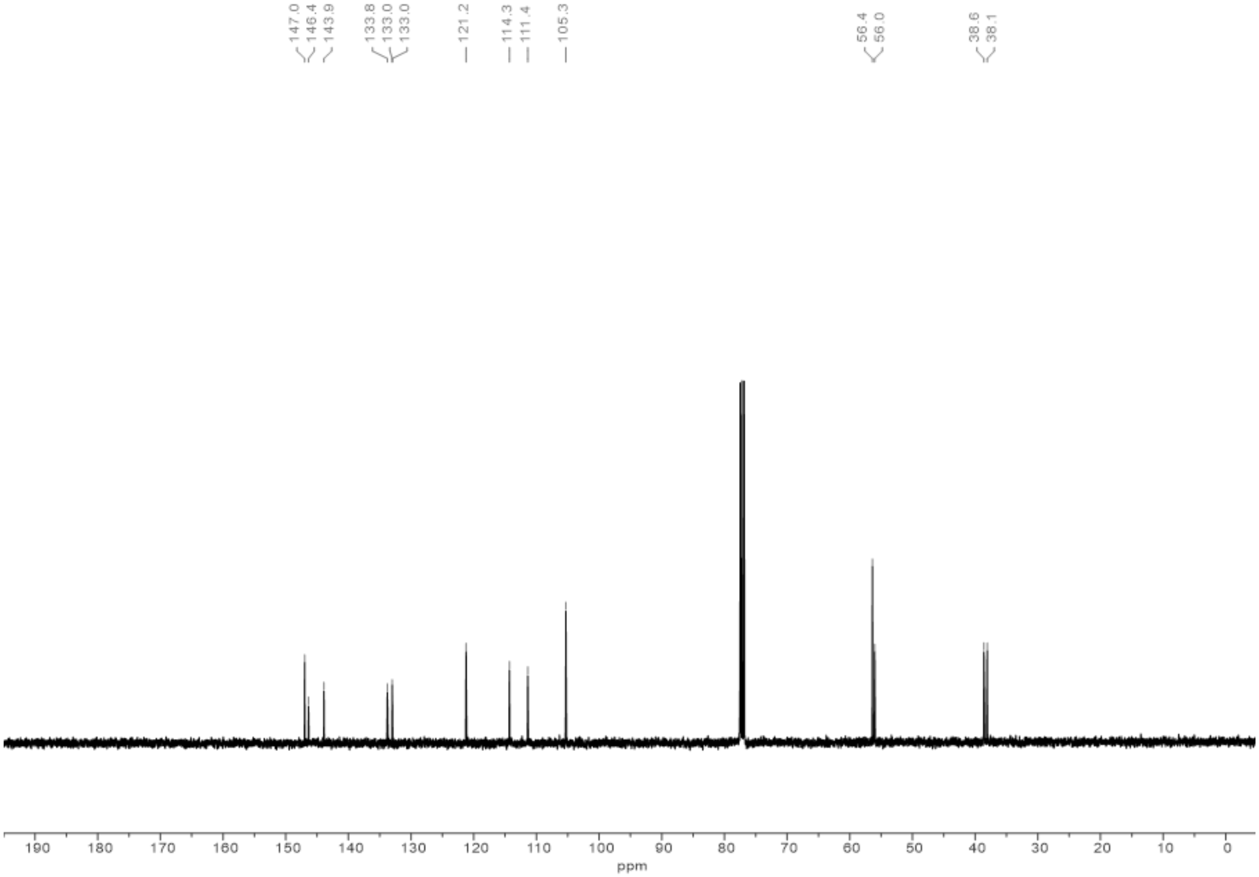

Isolation of Sesamin (D5)

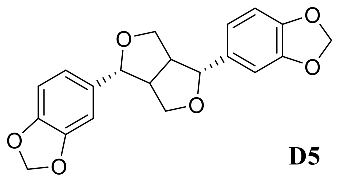

D5

Sesamin was isolated from commercially available Sesame oil in supermarket (100 mL) by column chromatography (SiO_2, 0-30% EtOAc/PE) to provide D5 as a white solid (0.56 g).

^{1}H NMR (400 MHz, $CDCl_3$) δ 6.85 (dd, J = 1.4, 0.7 Hz, 2H), 6.79 (dd, J = 1.6, 0.5 Hz, 2H), 6.79 (d, J = 0.6 Hz, 2H), 5.95 (s, 4H), 4.71 (d, J = 4.4 Hz, 2H), 4.32 – 4.16 (m, 2H), 3.87 (dd, J = 9.3, 3.7 Hz, 2H), 3.11 – 2.98 (m, 2H).

^{13}C NMR (101 MHz, $CDCl_3$) δ 148.1, 147.3, 135.2, 119.5, 108.3, 106.6, 101.2, 85.9, 71.9, 54.5.

HRMS (ESI$^+$) m/z: $[M + Na]^+$ calcd. for $C_{18}H_{22}NaO_6$ 377.0996; found, 377.0997

^{1}H NMR:

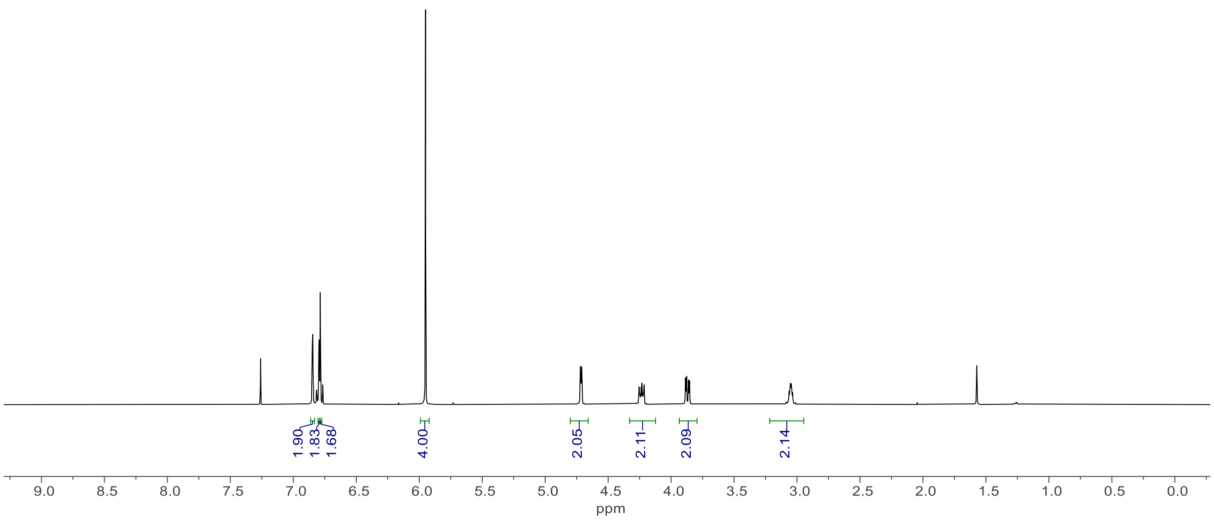

^{13}C NMR:

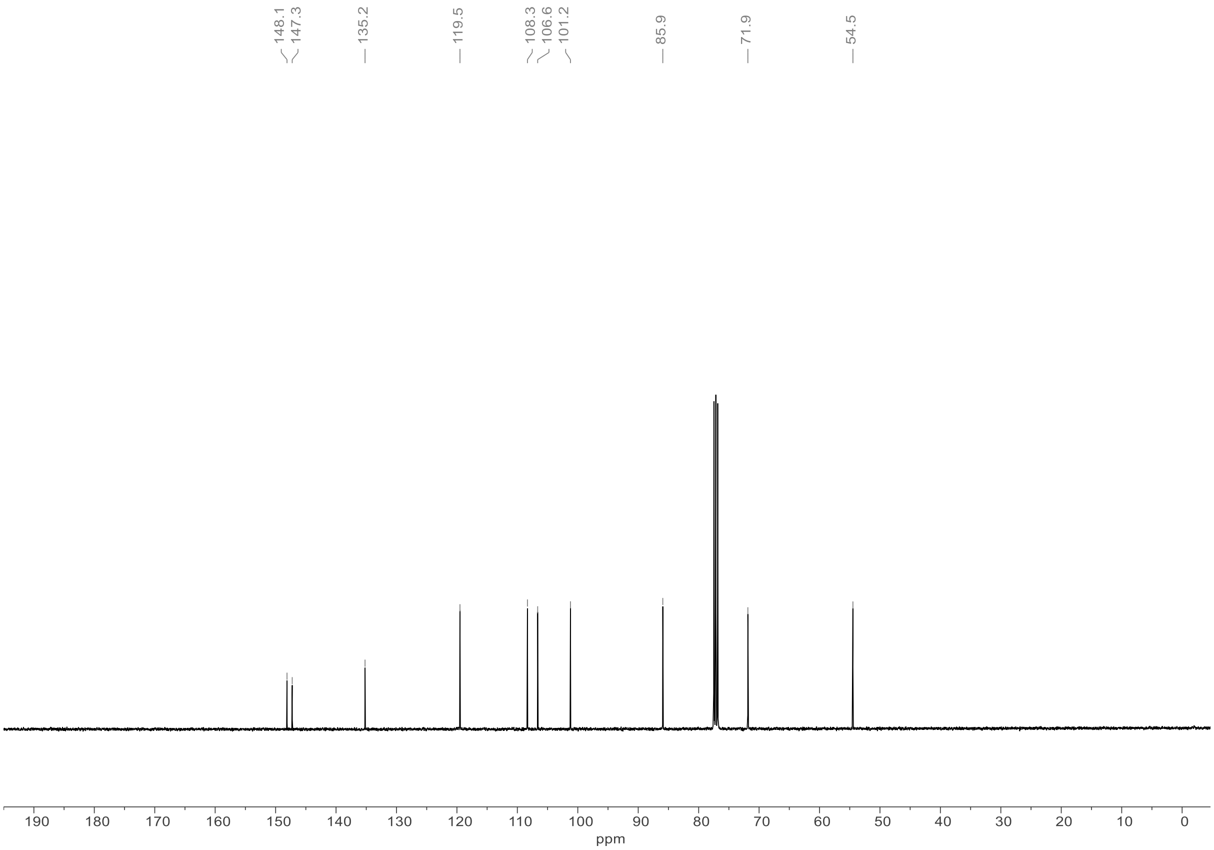

Synthesis of Syringaresinol (D7)

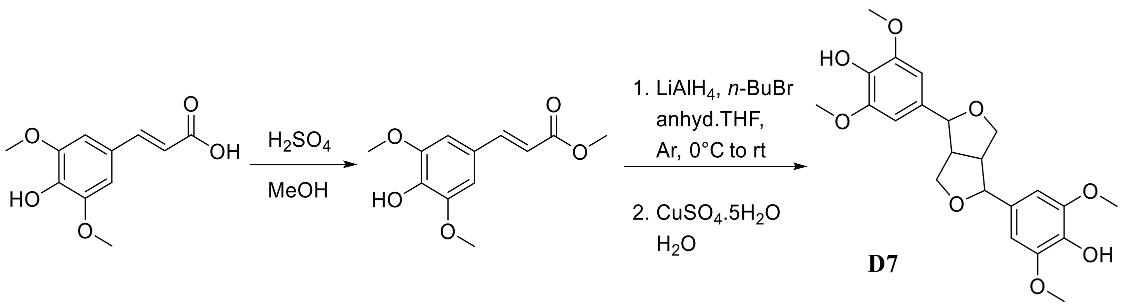

D7

Syringaresinol (D7) was prepared in 3 steps following the procedure from Lancefield *et al*.

Step 1: Sinapic acid (6.2 g, 27.6 mmol) was dissolved in MeOH (95 mL) and refluxed overnight with a small amount of H_2SO_4 (160 µL). After cooling down to room temperature, the solvent was removed by rotary evaporator. The crude product was obtained as white solid then washed with water and dried under reduced pressure to give methyl sinapate (6.0 g, 91% yield).

^{1}H NMR (400 MHz, $CDCl_3$): δ 7.58 (d, *J* = 15.9 Hz, 1H), 6.75 (s, 2H), 6.29 (d, *J* = 15.9 Hz, 1H), 5.83 (s, 1H), 3.90 (s, 6H), 3.78 (s, 3H).

Step 2: A flame dried schlenk flask under argon atmosphere was charged with $LiAlH_4$ (2.0 g, 53 mmol, 2.2 equiv.) and cooled down to -78 °C followed by addition of anhyd. THF (60 mL) and *n*-BuBr (2.2 equiv). After the reaction was allowed to stir at 0 °C for 3 h, the reaction was cooled down to -78 °C. Then, methyl sinapate (5.7 g 24 mmol 1 equiv.) was dissolved in anhyd. THF (60 mL) and transferred to the reaction mixture at -78 °C. The reaction mixture was stirred vigorously and allowed to reach room temperature for 3 hours. The reaction was quenched by addition of EtOAc (40 mL) slowly for removing excess of $LiAlH_4$, followed by slow addition of water (50 mL). After sat. NH_4Cl was added to the mixture, the resulting mixture was extracted with EtOAc (5 x 100 mL). The combined organic layers were washed with brine (100 mL), dried by Na_2SO_4, and concentrated by rotary evaporator. The crude intermediate was obtained as a sinapyl alcohol (yellow oil, 3.8 g, 18 mmol), which was used in the next step immediately.

Step 3: The solution of $CuSO_4.5H_2O$ (4.6 g, 18 mmol, 1 equiv.) in water (800 mL) was transferred to the reaction flask containing crude intermediate. The reaction mixture was stirred vigorously and left for 3 days in a normal atmosphere at room temperature. The reaction mixture was extracted with EtOAc (5 x 200 mL) then the combined organic layers were washed with brine (200 mL), dried by Na_2SO_4, and concentrated by rotary evaporator. The obtained crude product as a red gum was dissolved in EtOH (10 mL) and left to crystallize overnight. The crystals were harvested by filtration and washed with cold EtOH to provide syringaresinol (D7) as a white solid (1.2 g, 24%). Analytical data were in accordance with the previously reported.

^{1}H NMR (400 MHz, $CDCl_3$): δ 6.58 (s, 4H), 5.50 (s, 2H), 4.73 (d, *J* = 4.2 Hz, 2H), 4.32 – 4.25 (m, 2H), 3.93 – 3.87 (m, 2H), 3.90 (s, 12H), 3.14 – 3.05 (m, 2H).

^{13}C NMR (100 MHz, $CDCl_3$): δ 147.3, 134.4, 132.2, 102.8, 86.2, 71.9, 56.5, 54.5.

HRMS (ESI$^+$) *m/z*: $[M + Na]^+$ calcd. for $C_{22}H_{26}NaO_8$ 441.1520; found, 441.1525.

^{1}H NMR:

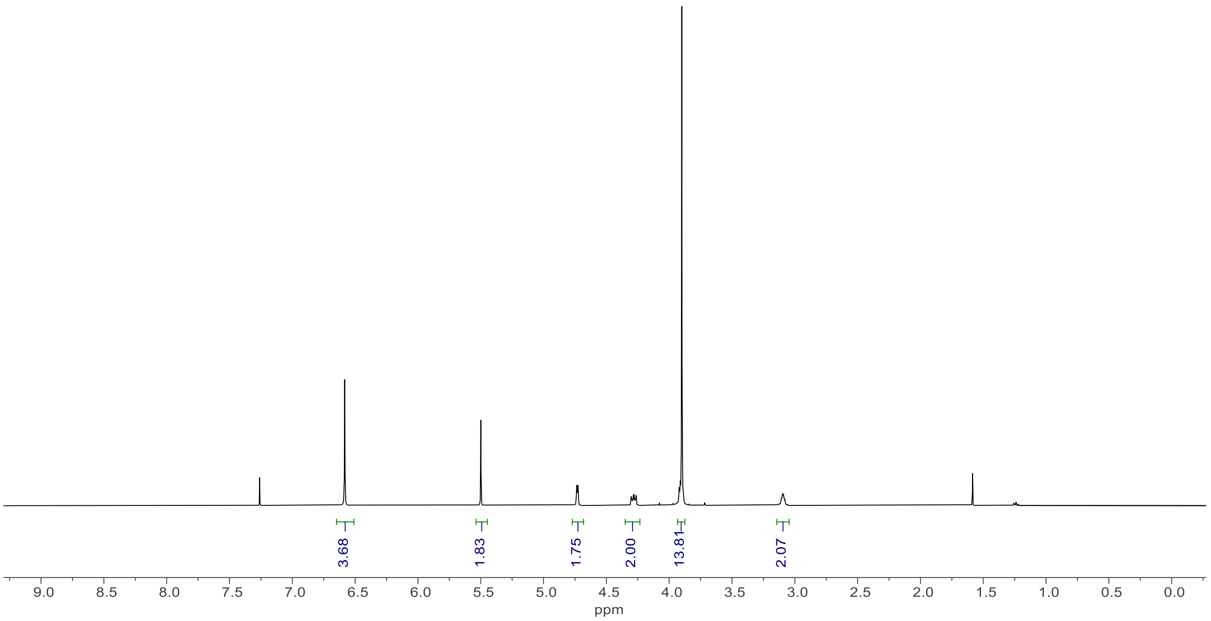

^{13}C NMR:

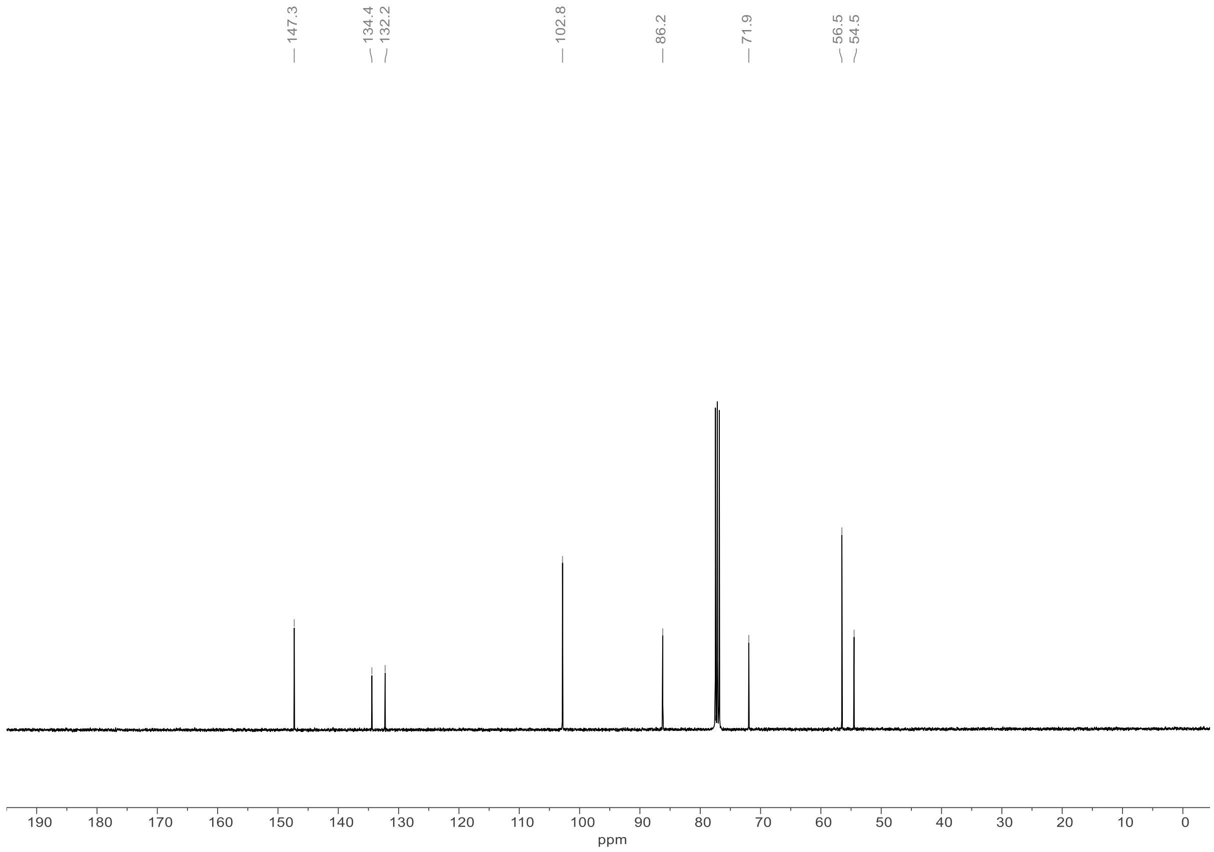

Synthesis of Pinoresinol (D6) and dimeric lignin model compound (D8)

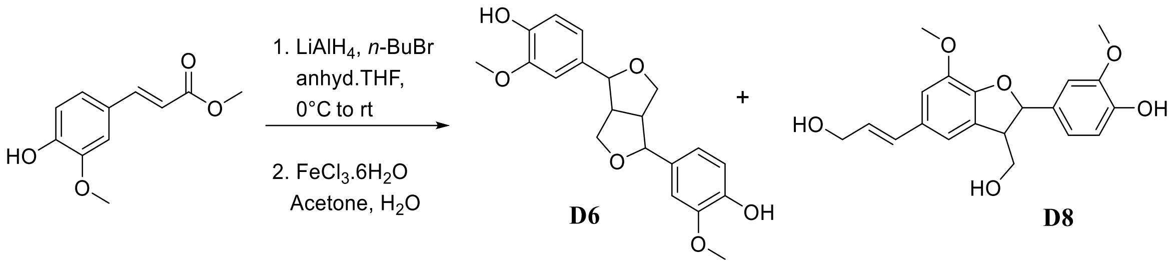

Syringaresinol (D6) and D8 were prepared in 2 steps sequentially following the procedure from Lancefield *et al.*[7]

Step 1: The commercially available methyl ferulate (3.3 g, 16 mmol) was reduced to coniferyl alcohol following the same method as for the preparation of compond D7 (see synthetic procedure for synthesis of Syringaresinol (D7), Step 2). Coniferyl alcohol was obtained as a yellow oil (2.8 g) which was used in the next step without purification.

Step 2: To dissolve coniferyl alcohol from the previous step (2.8 g) acetone (90 mL) was added the reaction flask containing water (900 mL) followed by addition a solution of $FeCl_3{*}6H_2O$ (5.4 g, 20 mmol) in water (40 mL). After the mixture was stirred vigorously for 1 hour, the mixture was extracted with EtOAc (5 x 200 mL). The combined organic layers were washed, sodium ascorbate (0.1 M, 125 mL), water (200 mL), brine (200 mL), and dried by Na_2SO_4. After the solvent was removed by rotary evaporator, the crude product was purified by column chromatography (SiO_2, 30-50% Acetone/PE) to give D6 and D8. The spectral data were in accordance with the reports[7,8].

Pinoresinol (D6): as a pale-yellow solid (0.31 g, 6%)

^{1}H NMR (400 MHz, $CDCl_3$): δ 6.92 – 6.85 (m, 4H), 6.81 (dd, *J* = 8.1, 1.9 Hz, 2H), 5.72 (s, 2H), 4.73 (d, *J* = 4.4 Hz, 2H), 4.30 – 4.18 (m, 2H), 3.89 (s, 6H), 3.90 – 3.80 (m, 2H), 3.15 – 3.04 (m, 2H)

^{13}C NMR (100 MHz, $CDCl_3$): δ 146.8, 145.4, 133.0, 119.1, 114.4, 108.7, 86.0, 71.8, 56.1, 54.3, 31.0.

HRMS (ESI$^+$) *m/z*: $[M + Na]^+$ calcd. for $C_{20}H_{22}NaO_6$ 381.1309; found, 381.1293

^{1}H NMR:

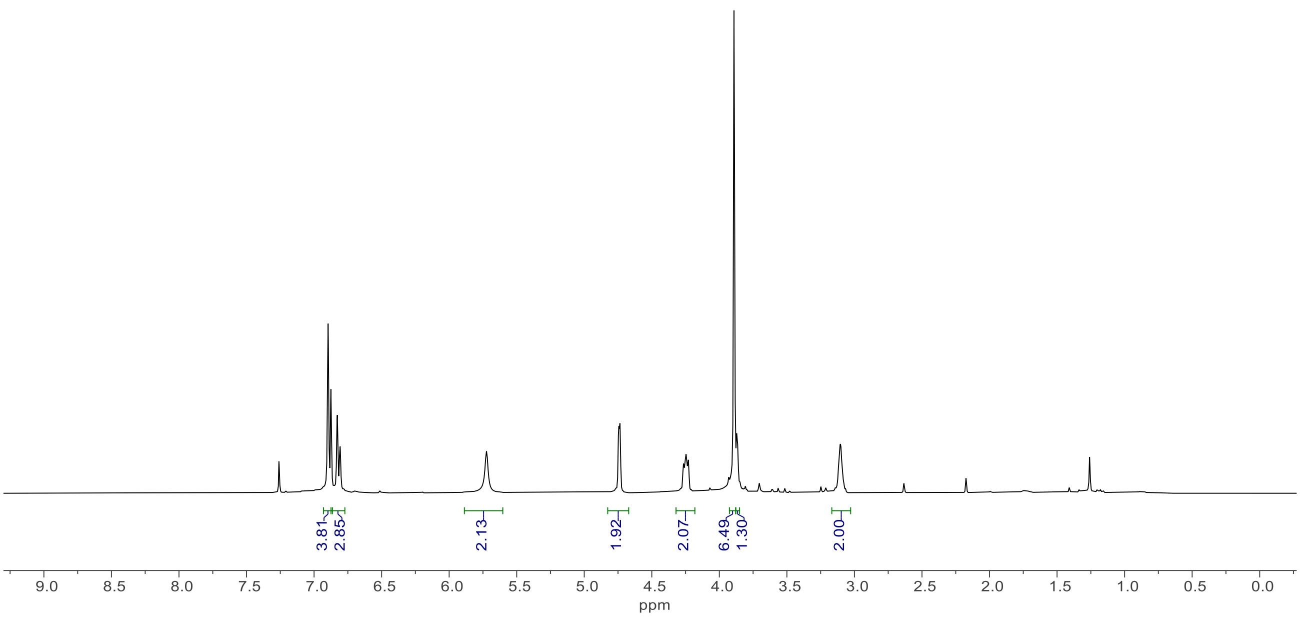

^{13}C NMR:

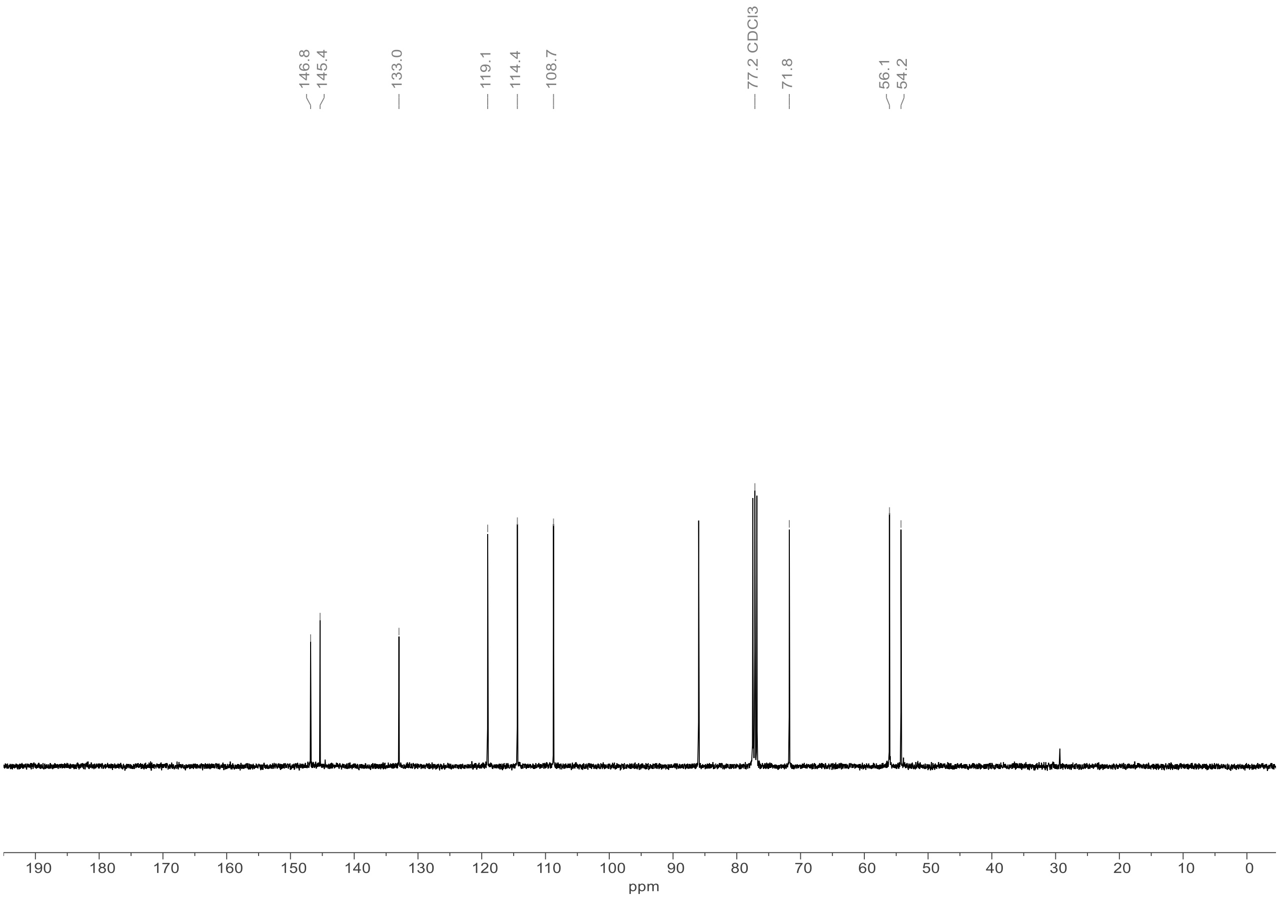

(E)-4-(3-(hydroxymethyl)-5-(3-hydroxyprop-1-en-1-yl)-7-methoxy-2,3-dihydrobenzofuran-2-yl)-2-methoxyphenol (D8): as a white solid (0.42 g, 8%)

^{1}H NMR (400 MHz, $CDCl_3$) δ 6.95 – 6.85 (m, 5H), 6.56 (d, *J* = 15.8 Hz, 1H), 6.25 (dt, *J* = 15.8, 5.9 Hz, 1H), 5.62 (s, 1H), 5.58 (d, *J* = 7.2 Hz, 1H), 4.31 (d, *J* = 5.9 Hz, 2H), 4.03 – 3.93 (m, 1H), 3.91 (s, 3H), 3.87 (s, 3H), 3.63 (q, *J* = 6.0 Hz, 1H).

^{13}C NMR (101 MHz, $CDCl_3$) δ 148.5, 146.8, 145.9, 144.6, 133.0, 131.5, 131.0, 128.2, 126.6, 119.6, 114.9, 114.5, 110.6, 108.9, 88.4, 64.1, 64.0, 56.1, 53.7.

HRMS (ESI$^+$) *m/z*: $[M + Na]^+$ calcd. for $C_{20}H_{22}NaO_6$ 381.1309; found, 381.1301

^{1}H NMR:

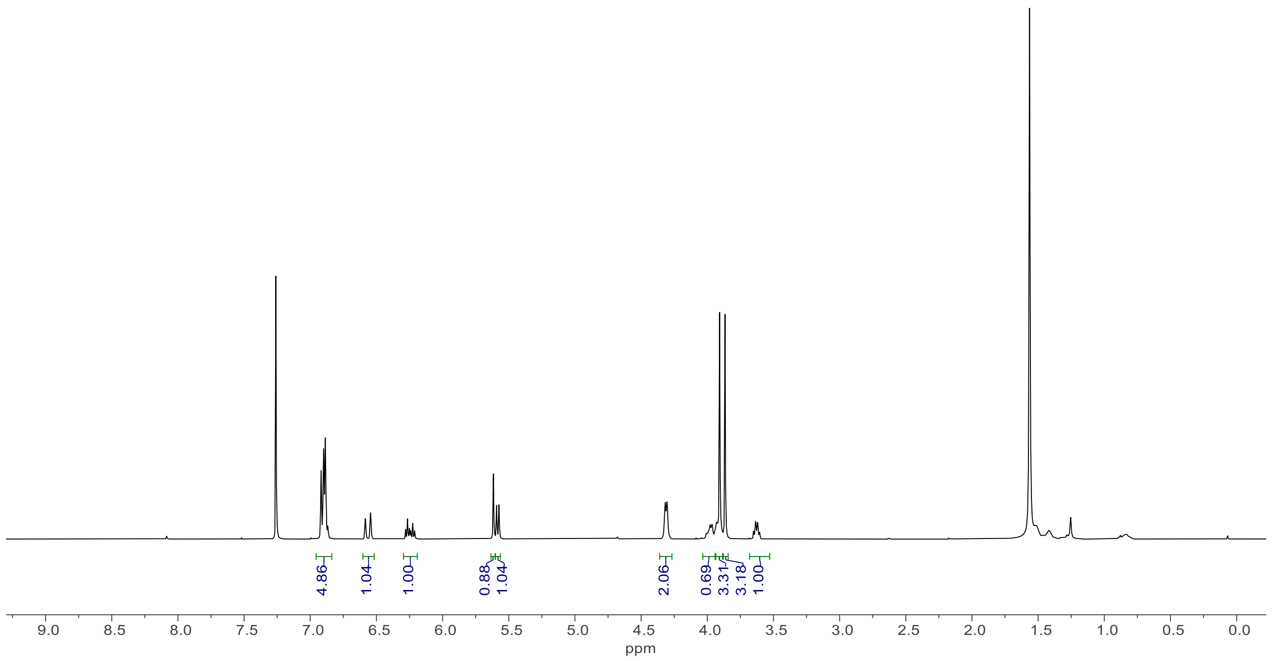

^{13}C NMR:

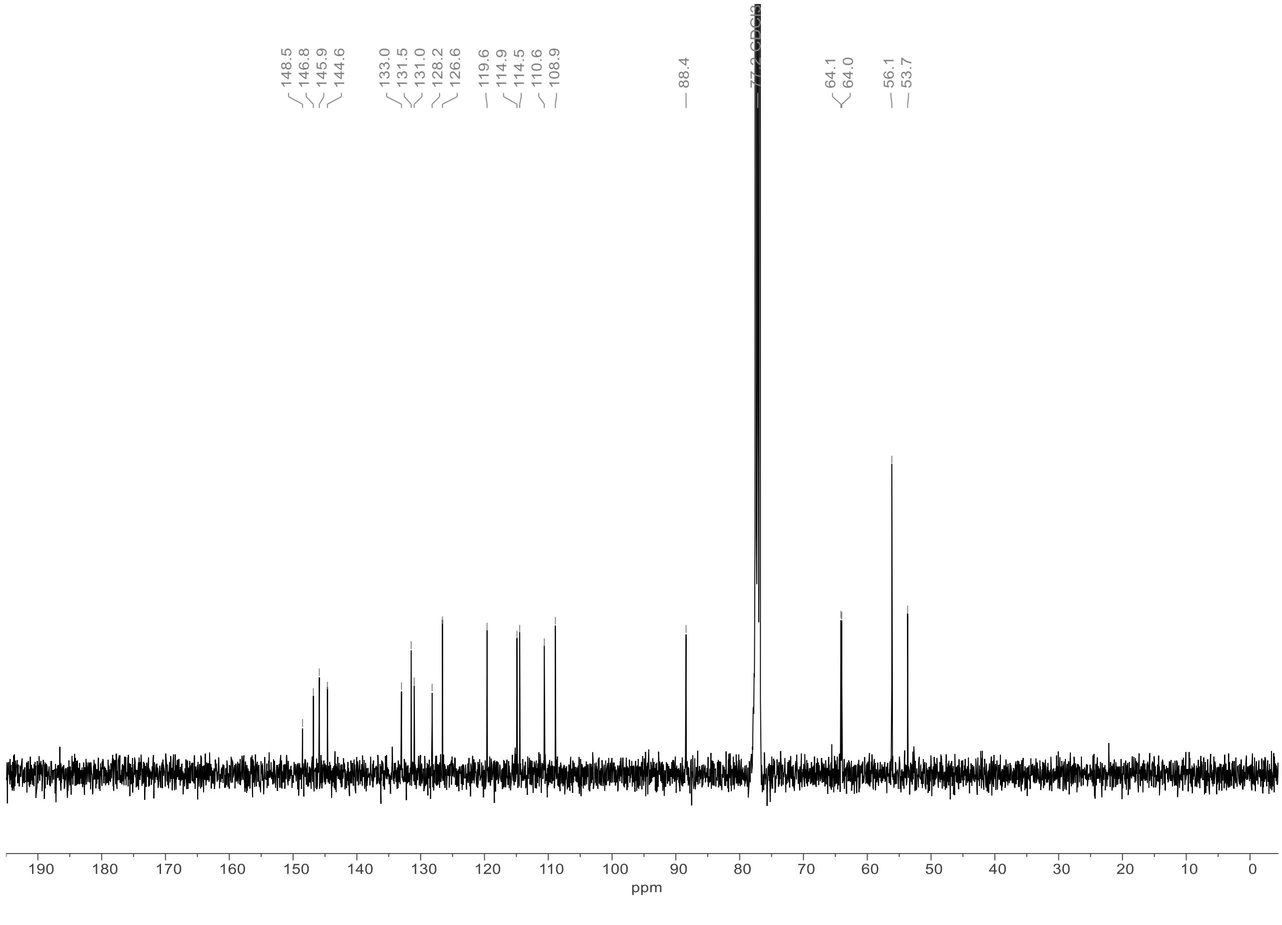

Table S1: Calibration ranges of analytes. Concentrations are presented in mg/L units

	C1	C2	C3	C4	C5
**All dimers**	125	75	45	30	15
**M1**	200	120	72	48	24
**M2**	1000	600	360	240	120
**M3**	500	300	180	120	60
**M4**	1000	600	360	240	120
**M5**	200	120	72	48	24
**M6**	200	120	72	48	24

Table S2: Concentration levels of standard mixtures for precision and bias measurements presented as mg/L

Analyte	Low level precision mixture	High level precision mixture
**All dimers**	22.5	52.5
**M1**	45	105
**M2**	225	525
**M3**	120	280
**M4**	150	350
**M5**	45	105
**M6**	30	70

Figure S1: Chemical structure of stationary phases used during the chromatographic method development

2-PIC: 2-picolylamine; DEA: diethylamine; DIOL: high density diol; 1-AA: 1-aminoanthracene

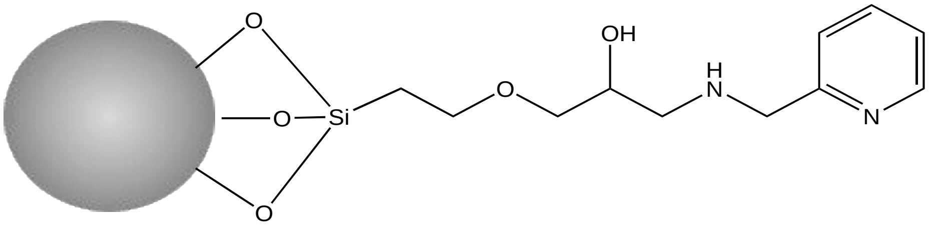

2-PIC

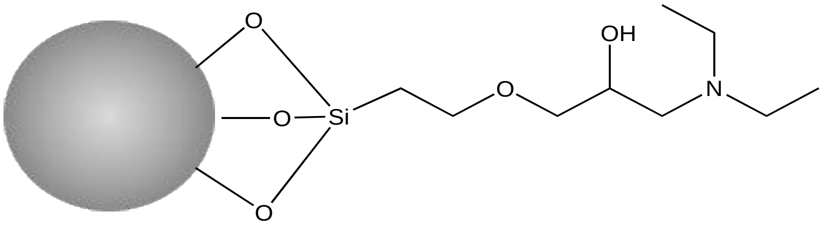

DEA

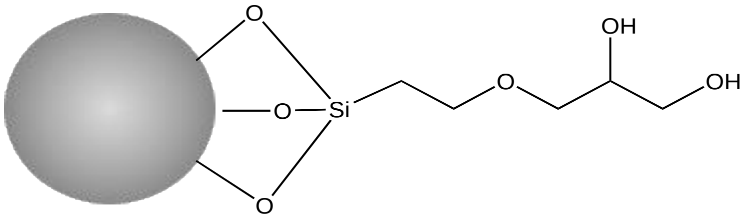

DIOL

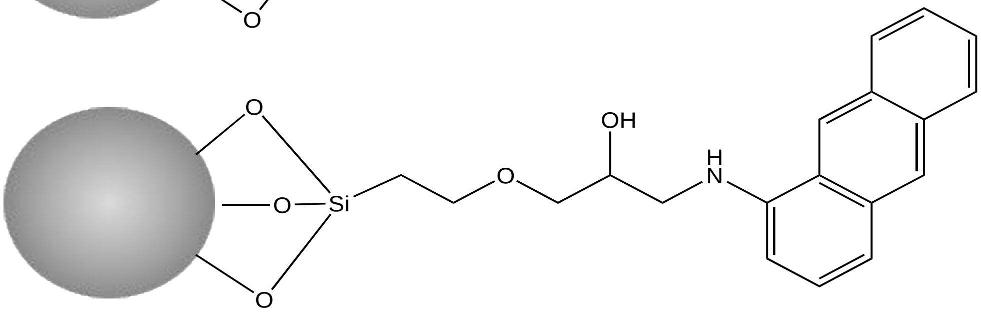

1-AA

Figure S2: Gradient profiles tested with the 1-AA column during method development

Method: BPR: 110 bar, column temperature: 50°C. Mobile phase: liquid CO_2 (solvent A) with MeOH cosolvent (solvent B), 1.6 ml/min. Make-up solvent: 5 mM NH_3 in MeOH, 0.8 ml/min. Green color marks the best performing gradient.

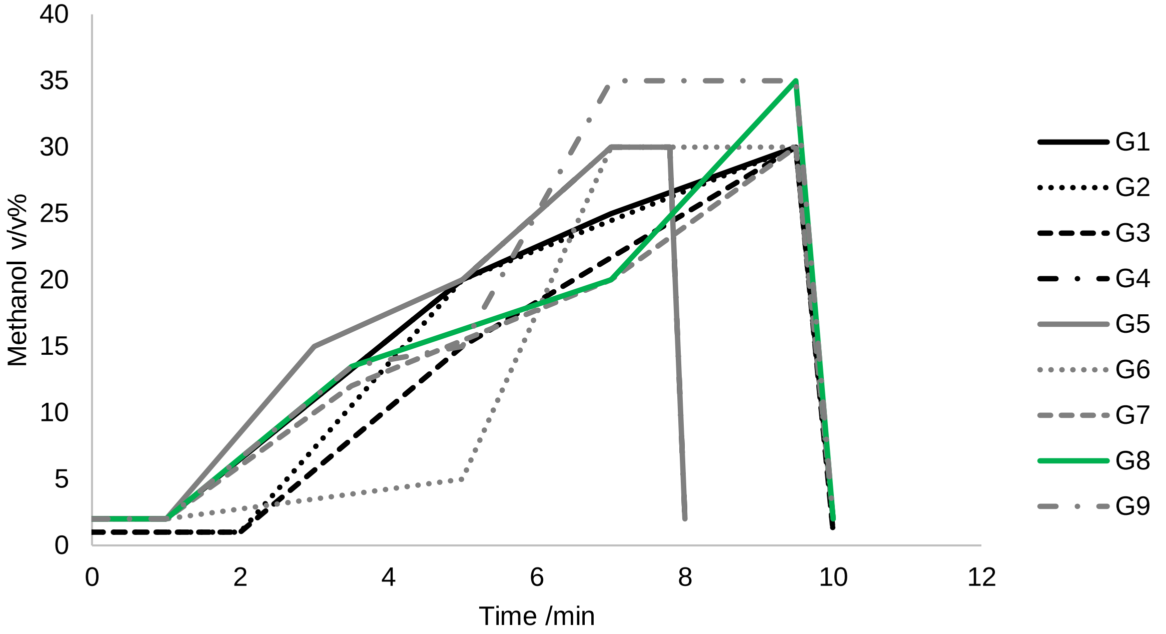

Figure S3: Best performing gradients with the tested columns during the SFC method development

Method: BPR: 110 bar, column temperature: 50°C. Mobile phase: liquid CO_2 (solvent A) with MeOH cosolvent (solvent B), 1.6 ml/min. Make-up solvent: 5 mM NH_3 in MeOH, 0.8 ml/min.

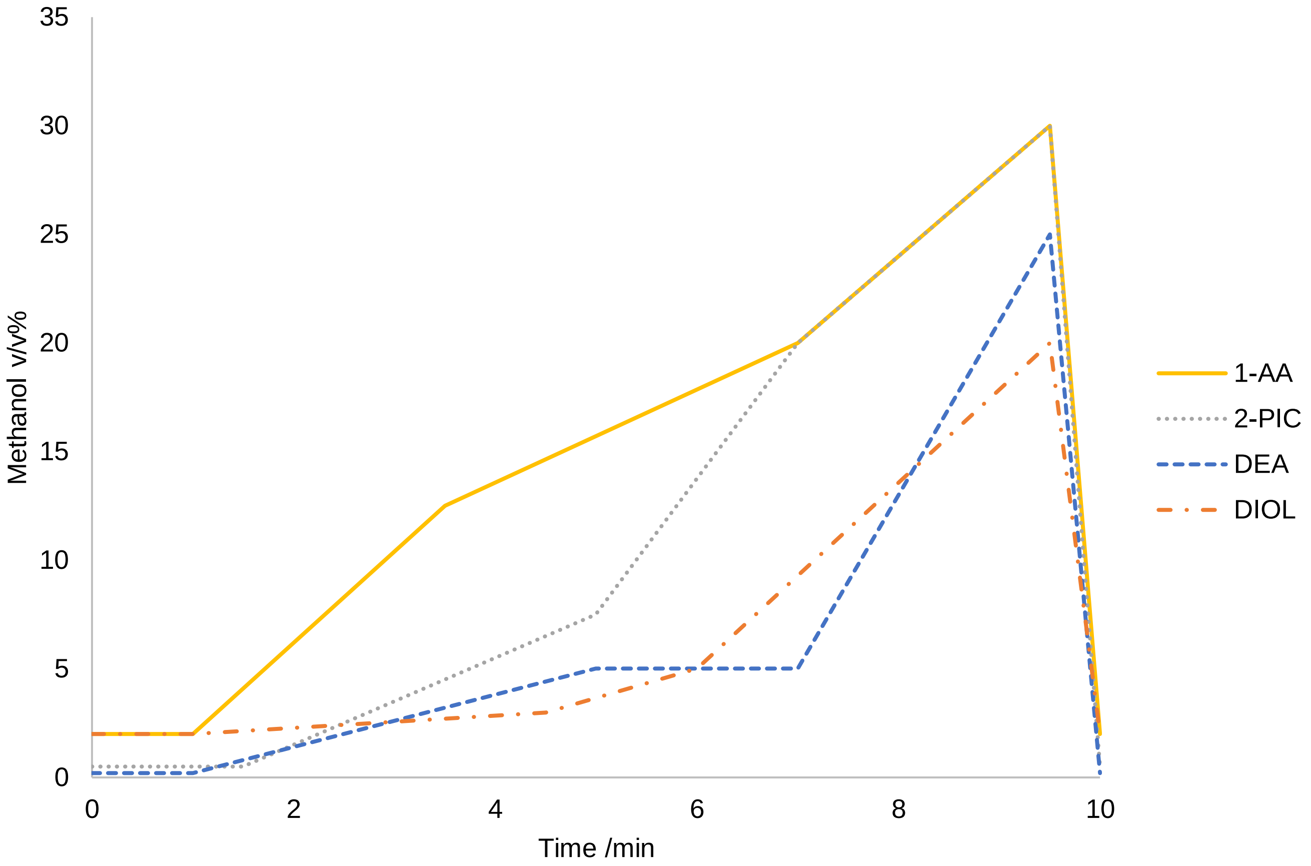

Figure S4: Scaled effects of variables of the experimental design

Variables in the experimental design are: F: mobile phase flow rate; T: mobile phase temperature; p: backpressure). Chromatographic resolution between the selected peak pairs was chosen as the experimental response.

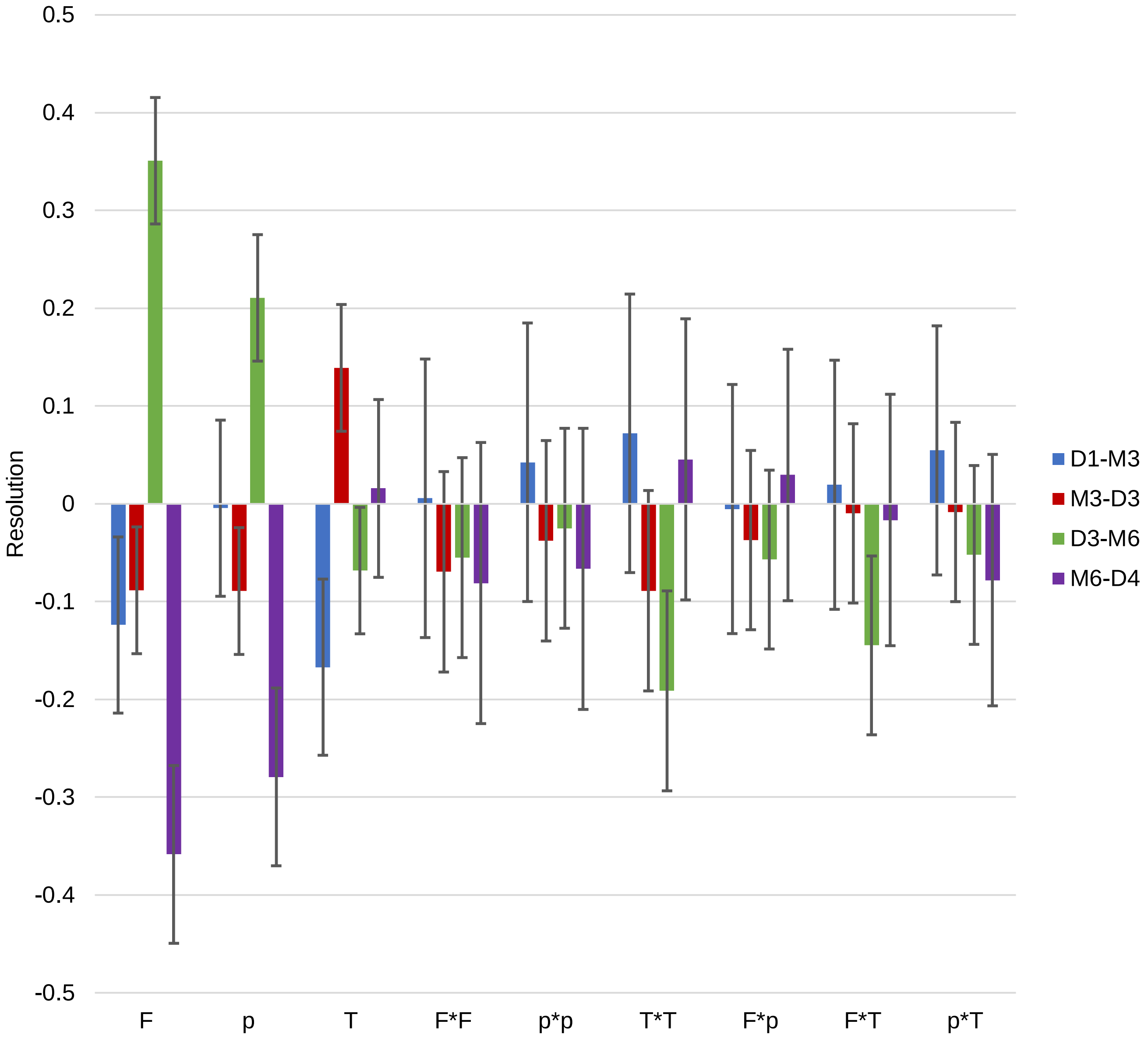

Table S3: Regression coefficients of the compound-specific calibration curves of lignin dimers in the DAD, CAD and FID

Calibration curves for the DAD were generated by linear regression using the signal at 210 nm. CAD calibration curves were established by both quadratic and linear regression, using the general quadratic regression equation in a form of $y = a \times x^2 + b \times x + c$, where x is analyte concentration and y is the area of the chromatographic peak. *Relative response factors for the flame ionization detector were predicted according to the procedure described by Thi et al.[9]

Analyte	DAD @210 nm		CAD quadratic curve			CAD linear curve		FID*
	Slope	Intercept	A	B	C	Slope	Intercept	Relative response factor
M1	4.14	13.97	-	-	-	-	-	1.81
M2	1.10	24.83	-	-	-	-	-	1.78
M3	1.95	17.17	0.0004	0.32	-0.13	0.54	-12.70	1.48
M4	0.35	6.13	-	-	-	-	-	2.05
M5	3.89	9.49	-	-	-	-	-	2.02
M6	3.59	12.30	0.0012	0.86	-8.34	1.09	-10.81	1.72
D1	4.00	-3.66	-0.0039	2.4334	-3.4821	1.87	9.81	2.30
D2	6.33	1.50	-0.0023	2.0587	-2.7409	1.74	4.93	2.36
D3	3.68	8.31	0.0012	1.4157	-1.4243	1.58	-5.33	1.88
D4	4.71	10.61	-0.0020	2.0053	-3.3075	1.72	3.37	2.12
D5	1.23	8.92	-0.0030	2.0493	0.3519	1.62	10.58	3.55
D6	1.77	7.54	-0.0001	1.3563	1.4155	1.35	1.64	2.63
D7	5.27	15.43	-0.0018	1.7696	-0.5166	1.52	5.52	3.11
D8	5.07	-35.65	0.0008	1.26	-0.54	1.34	1.86	2.20

S2: Detailed calculation workflow with example for the calculation of interday precision

The calculation uses the raw peak area data obtained from the CAD signal (dimers) or the DAD signal recorded at 210 nm (monomers). In this example, D2 is used as a model compound and the peak areas of a standard solution with 52.5 mg/L concentration are used to calculate the interday precision.

Peak area /mV*min	Replicate 1	Replicate 2	Replicate 3
Day 1	90.71	91.12	87.54
Day 2	79.57	92.53	85.65
Day 3	75.96	72.15	75.24

Generate all possible permutations of the raw data and compute the respective relative standard deviation

			RSD /%				RSD /%
90.71	79.57	75.96	9.36	91.12	92.53	72.15	13.35
90.71	79.57	72.15	11.56	91.12	92.53	75.24	11.12
90.71	79.57	75.24	9.75	91.12	85.65	75.96	9.11
90.71	92.53	75.96	10.51	91.12	85.65	72.15	11.77
90.71	92.53	72.15	13.25	91.12	85.65	75.24	9.60
90.71	92.53	75.24	11.02	87.54	79.57	75.96	7.31
90.71	85.65	75.96	8.91	87.54	79.57	72.15	9.65
90.71	85.65	72.15	11.58	87.54	79.57	75.24	7.72
90.71	85.65	75.24	9.40	87.54	92.53	75.96	9.96
91.12	79.57	75.96	9.63	87.54	92.53	72.15	12.63
91.12	79.57	72.15	11.81	87.54	92.53	75.24	10.45
91.12	79.57	75.24	10.01	87.54	85.65	75.96	7.48
91.12	92.53	75.96	10.61	87.54	85.65	72.15	10.26
				87.54	85.65	75.24	8.00

Calculate the mean of the computed relative standard deviations and report it as interday precision:

$$\text{Interday precision}_{\text{D2}} = \frac{\sum_i \text{RSD}_i}{n} = 10.22\%$$

Table S4: Validation results of the optimized SFC method for lignin monomers as detected by the DAD at 210 nm

Concentrations of low- and high-level precision mixtures are detailed in Table S2. *k* denotes the number of degrees of freedom. Precision values are reported as relative standard deviation.

Analyte	Intraday precision of peak area (*k*=6) /%		Interday precision of peak area (*k*=2) /%		Working range mg/L	R^2	LOD mg/L
	low level	high level	low level	high level			
M1	6.3	4.2	8.2	12.5	24-160	0.995	4.0
M2	6.5	4.0	7.6	11.6	120-800	0.994	6.7
M3	7.2	4.4	8.5	12.9	60-400	0.994	10
M4	11.1	4.2	11.4	15.4	120-800	0.994	20
M5	7.4	3.8	8.2	12.5	24-160	0.997	4.0
M6	7.4	4.6	8.4	12.9	24-160	0.994	4.0

Figure S5: Compound specific calibration curves of lignin dimers in the charged aerosol detector

Error bars represent standard deviation (n=3).

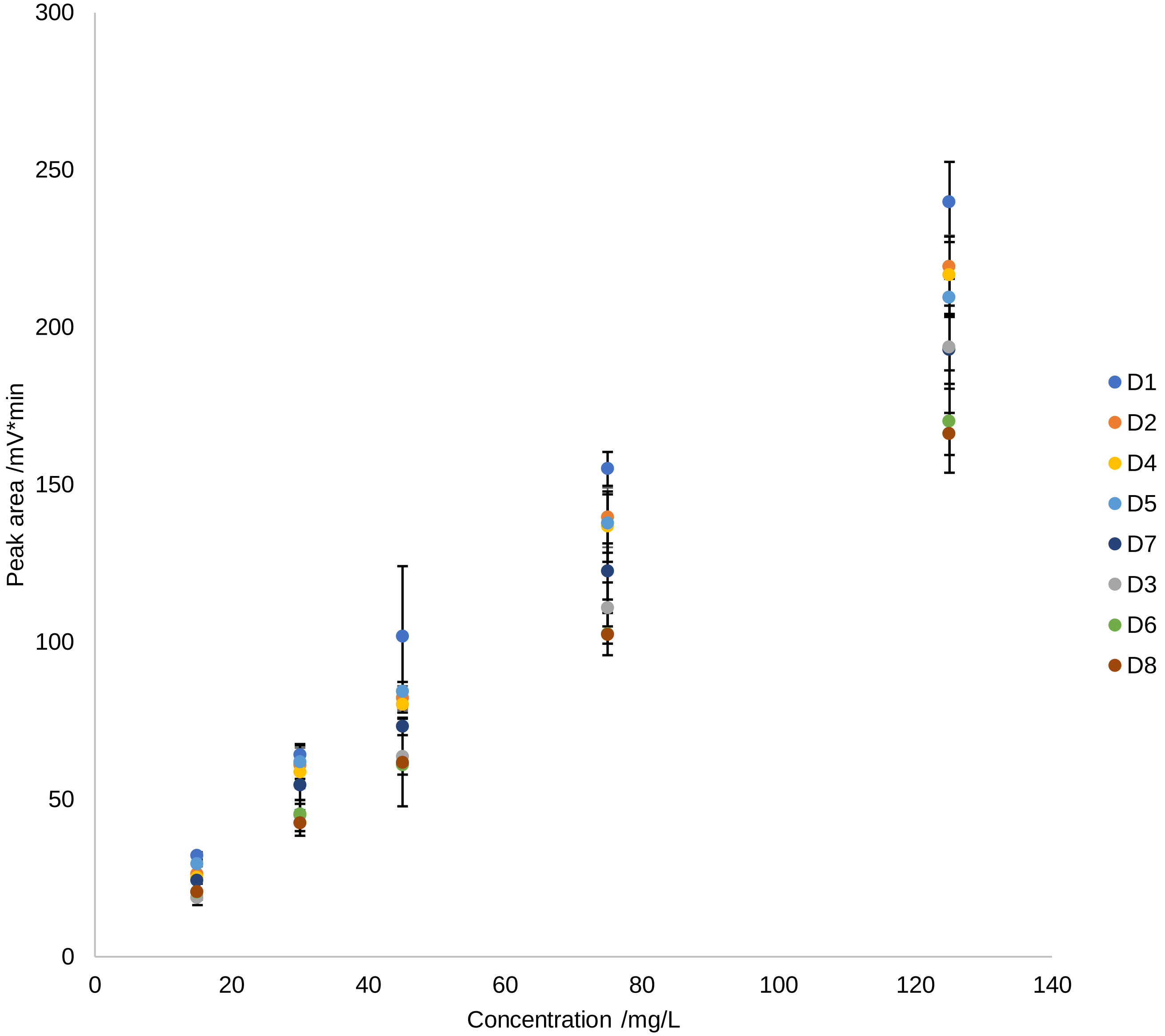

Figure S6: SFC-DAD (A) and SFC/QToF-MS (B) chromatograms of the dimer fraction of the birch oil

The SFC-CAD chromatogram was recorded at 210 nm and is presented without blank correction. SFC-QToF/MS chromatograms include the base peak ion chromatogram as well as the extracted ion chromatograms for the three dimers which were quantified by the CAD.

A

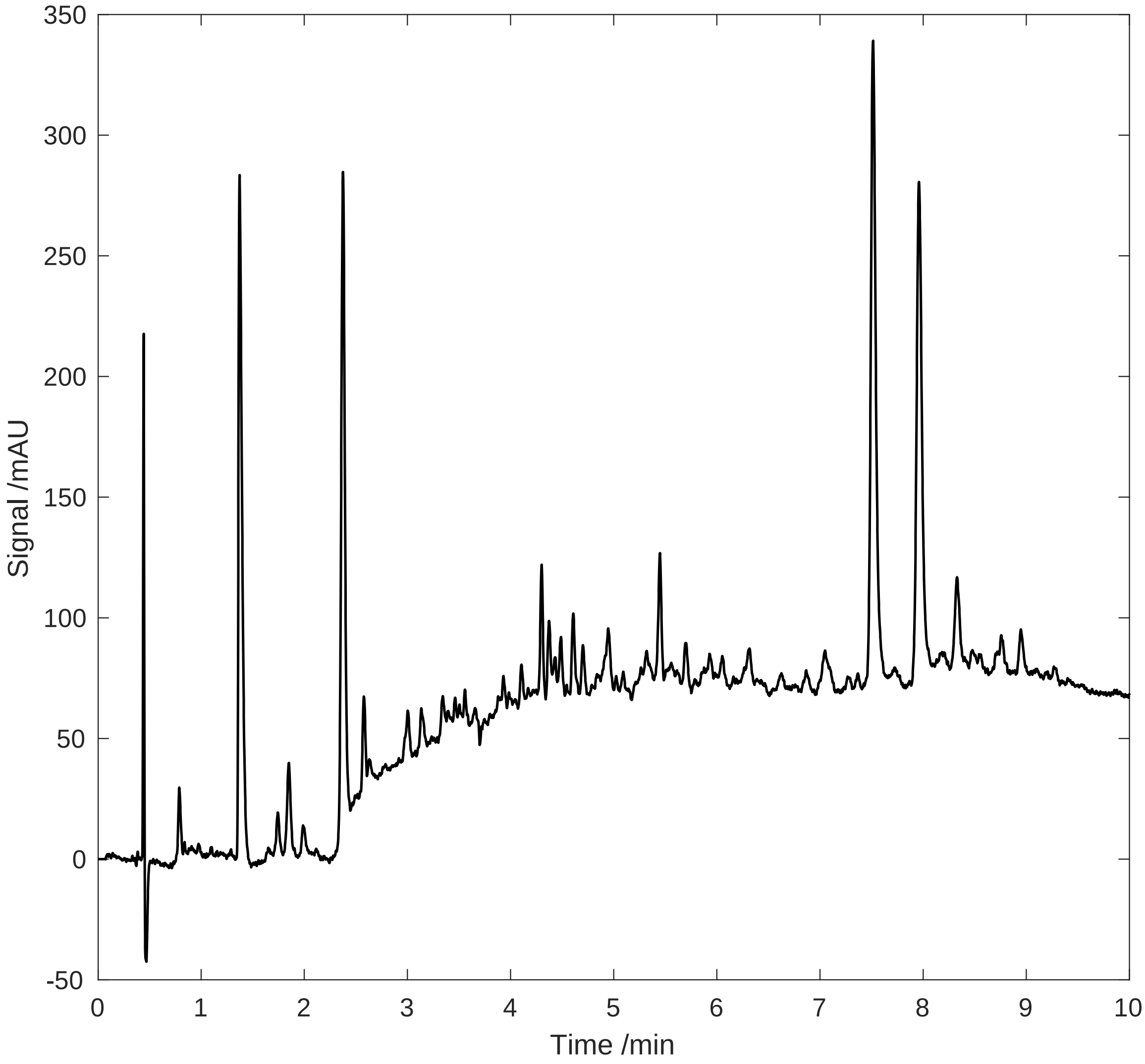

B

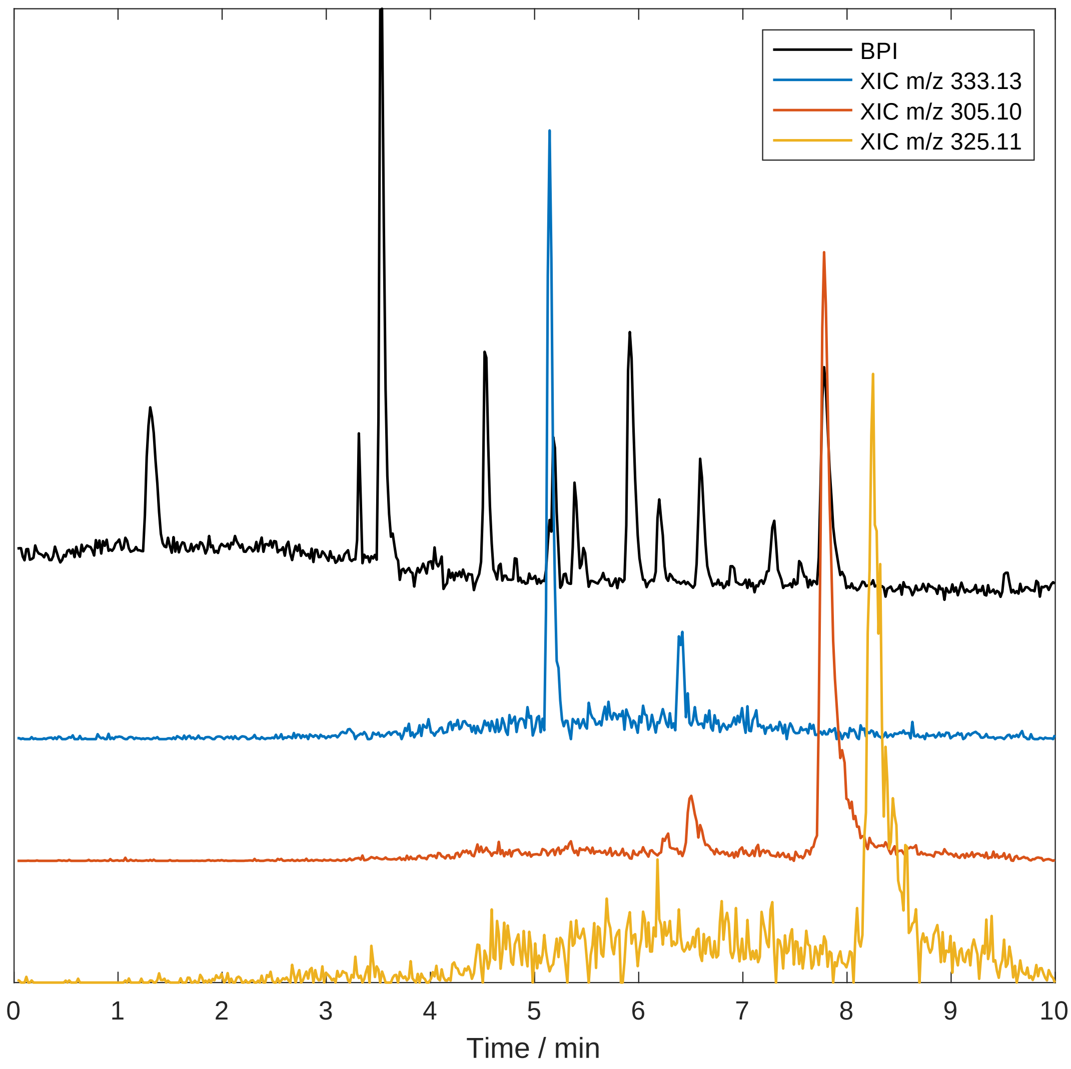

BPI
XIC m/z 333.13
XIC m/z 305.10
XIC m/z 325.11
0
1
2
3
4
5
6
7
8
9
10
Time / min

Table S5: Variables used for KMD-PCA-QDA classification of the detected peaks in the complete birch oil

#C: number of carbon atoms; #H: number of hydrogen atoms; #O: number of oxygen atoms. Kendrick mass defects are calculated for phenol (C_6H_5O), methoxy/primary alcohol (CH_3O), carboxylic acid (CHO_2), aldehyde (CHO) and secondary alcohol (CH_2O) groups

					Kendrick mass defect				
**Retention time in SFC/QToF-MS /min**	**Exact mass**	**#C**	**#H**	**#O**	**Phenol**	**Methoxy/primary alcohol**	**Carboxylic acid**	**Aldehyde**	**Secondary alcohol**
1.74	185.0802	9	14	4	-0.020075	0.022165	-0.097860	-0.070582	-0.022651
2.09	255.2318	16	32	2	-0.146064	-0.087901	-0.253173	-0.215611	-0.149612
2.19	193.0490	10	10	4	0.013946	0.057994	-0.067170	-0.038724	0.011259
2.50	151.0383	16	18	6	0.002744	0.072227	-0.125210	-0.080338	-0.001494
2.67	339.3259	7	6	2	0.008232	0.035933	-0.042779	-0.024890	0.006543
2.70	183.0646	9	10	4	0.009388	0.050712	-0.066713	-0.040025	0.006867
2.72	353.3425	9	8	3	0.013082	0.050319	-0.055490	-0.031442	0.010811
2.79	115.0389	11	12	4	0.003373	0.050603	-0.083602	-0.053101	0.000492
2.79	367.3569	19	20	3	-0.031391	0.035829	-0.155177	-0.111767	-0.035491
2.81	223.0593	11	12	5	0.014680	0.065540	-0.078980	-0.046135	0.011578
2.86	159.0640	7	12	4	-0.013458	0.022877	-0.080368	-0.056903	-0.015674
2.94	169.0857	9	14	3	-0.031458	0.007151	-0.102558	-0.077624	-0.033813
2.98	155.0697	8	12	3	-0.020580	0.014848	-0.085821	-0.062942	-0.022741
3.02	141.0544	7	10	3	-0.010388	0.021858	-0.069770	-0.048946	-0.012355
3.11	163.0389	22	44	3	-0.198775	-0.117893	-0.347721	-0.295488	-0.203709
3.14	177.0549	10	10	4	0.013580	0.057628	-0.067536	-0.039090	0.010893
3.16	155.0338	8	8	2	-0.002150	0.028732	-0.059020	-0.039077	-0.004034
3.21	269.2117	16	30	3	-0.120815	-0.059479	-0.233767	-0.194156	-0.124556
3.26	121.0284	9	8	3	0.013220	0.050456	-0.055352	-0.031305	0.010948
3.31	141.0547	7	10	3	-0.010770	0.021477	-0.070152	-0.049327	-0.012736
3.31	193.0494	8	6	3	0.023655	0.057711	-0.039058	-0.017065	0.021578
3.31	295.2265	18	32	3	-0.126143	-0.058902	-0.249970	-0.206546	-0.130245
3.33	135.0442	7	6	2	0.008324	0.036024	-0.042688	-0.024799	0.006634
3.43	181.0492	10	10	3	0.002906	0.043323	-0.071525	-0.045423	0.000441
3.50	221.0802	9	8	3	0.013631	0.050868	-0.054940	-0.030893	0.011360
3.52	295.2267	18	32	3	-0.126281	-0.059039	-0.250107	-0.206683	-0.130382
3.55	193.0491	10	10	3	0.002288	0.042706	-0.072143	-0.046041	-0.000177
3.55	207.0648	15	16	5	0.001804	0.064475	-0.113606	-0.073133	-0.002018
3.57	195.0629	7	8	3	0.003859	0.035648	-0.054681	-0.034152	0.001920
3.60	143.0335	6	8	4	0.011179	0.043875	-0.049030	-0.027915	0.009185
3.62	177.0541	9	10	3	-0.001607	0.036087	-0.071021	-0.046679	-0.003906
3.64	289.1806	18	26	3	-0.082442	-0.016573	-0.203741	-0.161203	-0.086460
3.65	205.0855	11	12	4	0.002717	0.049947	-0.084258	-0.053757	-0.000164
3.67	191.0704	18	22	6	-0.020248	0.055598	-0.159920	-0.110939	-0.024874
3.71	151.0388	21	24	8	0.000209	0.091945	-0.168725	-0.109482	-0.005386
3.72	311.2216	18	32	4	-0.115378	-0.044506	-0.245890	-0.200121	-0.119700
3.76	165.0544	10	10	3	0.002753	0.043171	-0.071677	-0.045575	0.000288
3.76	177.0542	10	10	2	-0.008447	0.028340	-0.076192	-0.052435	-0.010691

3.77	205.0490	7	8	3	0.004332	0.036120	-0.054208	-0.033679	0.002393
3.83	219.0652	12	12	4	0.007275	0.057229	-0.084715	-0.052455	0.004229
3.84	159.0652	7	12	4	-0.014624	0.021710	-0.081535	-0.058070	-0.016841
3.84	163.0384	9	8	2	0.002103	0.035709	-0.059783	-0.038080	0.000053
3.86	177.0542	19	22	3	-0.047071	0.020606	-0.171701	-0.127995	-0.051199
3.88	289.1806	18	26	3	-0.082442	-0.016573	-0.203741	-0.161203	-0.086460
3.89	137.0236	19	22	7	-0.002880	0.079320	-0.154253	-0.101168	-0.007893
3.91	121.0283	22	26	9	-0.001093	0.097455	-0.182573	-0.118930	-0.007104
3.93	149.0233	8	8	2	-0.002425	0.028457	-0.059295	-0.039351	-0.004309
3.93	309.2058	18	30	4	-0.100277	-0.029862	-0.229946	-0.184473	-0.104572
3.95	151.0386	19	20	3	-0.034228	0.032993	-0.158016	-0.114605	-0.038328
3.95	197.0804	10	14	4	-0.015890	0.029073	-0.098691	-0.069654	-0.018632
3.96	307.1889	18	28	4	-0.084138	-0.014182	-0.212965	-0.167787	-0.088405
4.00	135.0439	10	10	4	0.014007	0.058055	-0.067109	-0.038663	0.011320
4.02	149.0593	22	24	8	0.002144	0.096604	-0.171808	-0.110805	-0.003618
4.04	177.0544	11	10	4	0.017818	0.064590	-0.068314	-0.038109	0.014965
4.06	225.0764	11	14	5	-0.001695	0.049624	-0.096198	-0.063057	-0.004825
4.09	165.0544	9	12	3	-0.016372	0.021779	-0.086629	-0.061991	-0.018699
4.09	205.0491	10	8	4	0.028605	0.072196	-0.051669	-0.023518	0.025946
4.09	233.0816	13	14	4	-0.003908	0.049227	-0.101758	-0.067443	-0.007149
4.11	191.0705	10	8	4	0.028071	0.071662	-0.052203	-0.024052	0.025412
4.11	299.2592	18	36	3	-0.157352	-0.089196	-0.282864	-0.238849	-0.161509
4.14	139.0390	8	8	3	0.008814	0.043326	-0.054742	-0.032454	0.006709
4.14	175.0761	19	22	4	-0.039265	0.032044	-0.170581	-0.124530	-0.043614
4.16	153.0544	8	6	3	0.023564	0.057619	-0.039149	-0.017157	0.021487
4.16	327.2910	20	40	3	-0.178895	-0.104376	-0.316125	-0.268000	-0.183440
4.18	191.0336	19	20	3	-0.031757	0.035463	-0.155544	-0.112133	-0.035857
4.19	161.0597	22	24	8	0.001961	0.096421	-0.171991	-0.110988	-0.003801
4.19	185.0811	9	14	4	-0.021020	0.021219	-0.098806	-0.071528	-0.023597
4.19	293.1782	17	26	4	-0.078599	-0.011823	-0.201569	-0.158445	-0.082672
4.19	309.2067	18	30	4	-0.101177	-0.030762	-0.230847	-0.185373	-0.105471
4.23	165.0546	11	12	3	-0.008148	0.035451	-0.088437	-0.060281	-0.010807
4.23	189.0543	11	10	3	0.007251	0.050392	-0.072196	-0.044335	0.004619
4.26	203.0705	12	12	3	-0.003803	0.042520	-0.089108	-0.059193	-0.006628
4.28	167.0703	10	10	3	0.002906	0.043323	-0.071525	-0.045423	0.000441
4.31	205.0497	22	44	2	-0.209373	-0.132122	-0.351633	-0.301745	-0.214085
4.31	233.0811	13	14	4	-0.003465	0.049670	-0.101315	-0.067000	-0.006706
4.33	177.0549	9	12	3	-0.016799	0.021352	-0.087056	-0.062418	-0.019127
4.33	181.0857	10	14	3	-0.027060	0.014273	-0.103175	-0.076483	-0.029581
4.33	207.0652	17	18	6	0.003017	0.075224	-0.129955	-0.083324	-0.001388
4.35	191.0705	17	20	5	-0.019647	0.049387	-0.146774	-0.102192	-0.023858
4.35	219.0655	12	12	4	0.007024	0.056978	-0.084967	-0.052707	0.003977
4.36	125.0596	7	10	2	-0.021474	0.007141	-0.074171	-0.055691	-0.023220
4.38	155.0701	8	12	3	-0.021038	0.014390	-0.086278	-0.063399	-0.023198
4.42	137.0236	7	6	3	0.018914	0.050245	-0.038783	-0.018550	0.017003

4.42	151.0390	6	6	4	0.026372	0.058610	-0.032995	-0.012176	0.024406
4.42	153.0547	10	10	2	-0.008440	0.028348	-0.076184	-0.052427	-0.010683
4.42	183.0652	8	10	2	-0.017732	0.013608	-0.075445	-0.055205	-0.019643
4.45	141.0545	7	10	3	-0.010510	0.021736	-0.069893	-0.049068	-0.012477
4.45	161.0597	9	6	4	0.038545	0.078955	-0.035870	-0.009774	0.036080
4.45	165.0547	8	10	3	-0.006341	0.028629	-0.070739	-0.048155	-0.008474
4.45	193.0495	8	8	3	0.009302	0.043814	-0.054254	-0.031966	0.007197
4.45	239.0917	12	16	5	-0.011871	0.042628	-0.112233	-0.077037	-0.015195
4.47	147.0441	10	10	3	0.002372	0.042790	-0.072059	-0.045957	-0.000093
4.47	163.0388	10	8	4	0.027888	0.071479	-0.052386	-0.024235	0.025229
4.47	309.2066	18	30	4	-0.101131	-0.030716	-0.230801	-0.185327	-0.105426
4.50	217.0860	13	14	3	-0.014246	0.035258	-0.105410	-0.073440	-0.017266
4.52	123.0443	19	18	3	-0.016534	0.050229	-0.139478	-0.096363	-0.020606
4.52	173.0807	8	14	4	-0.025037	0.014478	-0.097807	-0.072287	-0.027448
4.52	187.0966	9	16	4	-0.035809	0.006888	-0.114437	-0.086863	-0.038413
4.52	209.0807	11	10	4	0.018306	0.065078	-0.067826	-0.037621	0.015453
4.54	125.0598	7	10	2	-0.021722	0.006894	-0.074419	-0.055939	-0.023468
4.54	137.0602	11	10	4	0.018405	0.065178	-0.067727	-0.037521	0.015553
4.54	167.0699	9	8	3	0.012594	0.049831	-0.055978	-0.031931	0.010323
4.54	191.0342	19	18	5	0.001459	0.075484	-0.134859	-0.087054	-0.003056
4.54	201.1116	10	18	4	-0.045695	0.000183	-0.130182	-0.100554	-0.048494
4.54	355.3212	12	14	3	-0.018073	0.028708	-0.104220	-0.074009	-0.020926
4.57	127.0389	6	8	3	-0.000021	0.029044	-0.053545	-0.034775	-0.001794
4.57	189.0546	11	10	3	0.006991	0.050133	-0.072455	-0.044594	0.004360
4.57	219.0655	12	12	4	0.007062	0.057016	-0.084929	-0.052669	0.004015
4.59	203.0705	12	12	3	-0.003841	0.042482	-0.089146	-0.059231	-0.006667
4.59	207.0654	16	18	7	0.013663	0.086776	-0.120977	-0.073761	0.009203
4.60	153.0547	7	6	3	0.018899	0.050230	-0.038799	-0.018565	0.016988
4.64	149.0232	7	8	4	0.015341	0.050761	-0.049884	-0.027011	0.013181
4.64	177.0548	9	8	2	0.002103	0.035709	-0.059783	-0.038080	0.000053
4.66	193.0495	17	20	6	-0.007692	0.064972	-0.141504	-0.094578	-0.012124
4.67	147.0441	21	26	7	-0.026588	0.061975	-0.189680	-0.132486	-0.031990
4.67	303.1229	8	4	1	-0.004469	0.021872	-0.052977	-0.035966	-0.006076
4.69	95.0495	8	8	3	0.008600	0.043113	-0.054956	-0.032668	0.006495
4.69	139.0389	9	10	3	-0.002461	0.035233	-0.071876	-0.047533	-0.004760
4.69	203.0335	11	8	4	0.033117	0.079432	-0.052172	-0.022262	0.030293
4.74	191.0337	11	12	3	-0.008194	0.035406	-0.088483	-0.060326	-0.010853
4.74	195.0656	19	18	4	-0.007263	0.063131	-0.136894	-0.091434	-0.011556
4.78	219.0656	12	12	4	0.006909	0.056863	-0.085082	-0.052822	0.003863
4.79	127.0391	6	8	3	-0.000242	0.028823	-0.053766	-0.034996	-0.002015
4.79	163.0394	10	10	4	0.013473	0.057522	-0.067643	-0.039197	0.010787
4.79	175.0393	10	8	3	0.017153	0.057113	-0.056435	-0.030629	0.014715
4.81	161.0602	11	14	4	-0.011812	0.035875	-0.099629	-0.068833	-0.014721
4.83	203.0701	12	12	3	-0.003475	0.042848	-0.088780	-0.058865	-0.006300
4.83	205.0496	11	12	2	-0.019646	0.020323	-0.093249	-0.067437	-0.022084

4.83	217.0500	12	10	4	0.021744	0.071240	-0.069405	-0.037440	0.018725
4.84	95.0497	11	12	3	-0.008186	0.035413	-0.088475	-0.060319	-0.010845
4.84	315.2544	18	36	4	-0.146648	-0.074860	-0.278846	-0.232485	-0.151026
4.86	139.0394	9	10	3	-0.001820	0.035874	-0.071235	-0.046892	-0.004120
4.86	231.0659	13	12	4	0.010964	0.063642	-0.086043	-0.052024	0.007751
4.88	191.0344	10	8	4	0.028620	0.072211	-0.051654	-0.023503	0.025961
4.90	125.0236	19	18	6	0.013201	0.090856	-0.129802	-0.079653	0.008465
4.90	279.1241	15	20	5	-0.029557	0.034029	-0.146653	-0.105589	-0.033436
4.91	109.0288	7	8	2	-0.006907	0.021251	-0.058761	-0.040577	-0.008625
4.91	245.0814	6	6	2	0.003445	0.028422	-0.042551	-0.026421	0.001921
4.95	189.0548	11	10	3	0.006702	0.049843	-0.072745	-0.044884	0.004070
4.95	215.0706	13	12	3	0.000428	0.049474	-0.089894	-0.058219	-0.002564
4.98	163.0397	10	10	2	-0.008897	0.027890	-0.076642	-0.052885	-0.011141
4.98	175.0391	10	8	3	0.017290	0.057250	-0.056298	-0.030492	0.014853
4.98	201.0549	12	10	3	0.011001	0.056866	-0.073462	-0.043842	0.008203
4.98	219.0656	12	12	4	0.006925	0.056878	-0.085066	-0.052806	0.003878
5.01	315.2543	18	36	4	-0.146556	-0.074769	-0.278754	-0.232394	-0.150935
5.04	265.1082	14	18	5	-0.018786	0.041619	-0.130023	-0.091013	-0.022470
5.09	163.0390	6	6	3	0.014527	0.043135	-0.038154	-0.019680	0.012782
5.09	177.0186	11	12	4	0.002961	0.050191	-0.084014	-0.053513	0.000080
5.11	219.0656	12	12	4	0.006970	0.056924	-0.085021	-0.052761	0.003924
5.11	245.0816	9	10	2	-0.012388	0.021675	-0.075116	-0.053118	-0.014466
5.13	389.1613	8	8	3	0.008997	0.043509	-0.054559	-0.032271	0.006892
5.14	333.1345	8	10	3	-0.006364	0.028607	-0.070762	-0.048178	-0.008497
5.18	217.0500	12	10	4	0.021820	0.071316	-0.069329	-0.037364	0.018801
5.18	309.1340	16	22	6	-0.028526	0.041872	-0.158165	-0.112702	-0.032819
5.19	161.0234	9	6	3	0.027863	0.064642	-0.039866	-0.016115	0.025620
5.21	175.0394	10	8	3	0.016985	0.056945	-0.056603	-0.030797	0.014547
5.21	189.0183	10	6	4	0.043180	0.086313	-0.036252	-0.008396	0.040549
5.30	243.1231	12	20	5	-0.041783	0.013631	-0.143831	-0.108044	-0.045163
5.35	231.0660	13	12	4	0.010903	0.063581	-0.086104	-0.052085	0.007690
5.37	191.0705	9	10	3	-0.002690	0.035004	-0.072105	-0.047762	-0.004989
5.37	203.0704	12	12	3	-0.003719	0.042604	-0.089024	-0.059109	-0.006544
5.38	295.1182	15	20	6	-0.017876	0.049340	-0.141657	-0.098249	-0.021976
5.40	217.0499	12	10	4	0.021919	0.071415	-0.069230	-0.037265	0.018900
5.45	251.0922	13	16	5	-0.007969	0.049255	-0.113347	-0.076392	-0.011459
5.49	205.0497	24	48	2	-0.230153	-0.146540	-0.384131	-0.330133	-0.235253
5.50	151.0393	10	12	4	0.000874	0.045379	-0.081084	-0.052343	-0.001841
5.52	165.0552	9	10	3	-0.001927	0.035767	-0.071342	-0.046999	-0.004226
5.61	167.0339	9	10	4	0.009433	0.050758	-0.066667	-0.039979	0.006913
5.66	279.1225	15	20	5	-0.028047	0.035539	-0.145142	-0.104078	-0.031925
5.79	343.1555	18	20	6	-0.003896	0.071492	-0.142725	-0.094039	-0.008494
5.83	143.0340	6	8	4	0.010661	0.043356	-0.049549	-0.028434	0.008667
5.83	233.0816	13	14	4	-0.003938	0.049197	-0.101788	-0.067473	-0.007179
5.91	281.1016	14	18	6	-0.006357	0.057678	-0.124280	-0.082926	-0.010263

5.97	153.0547	22	24	8	0.005774	0.100234	-0.168176	-0.107174	0.000013
6.01	277.1067	15	18	5	-0.012977	0.050152	-0.129229	-0.088461	-0.016827
6.13	233.0803	13	14	4	-0.002688	0.050447	-0.100537	-0.066222	-0.005928
6.16	191.0328	9	8	3	0.012335	0.049572	-0.056237	-0.032190	0.010064
6.18	289.1066	14	14	4	0.000666	0.056525	-0.102200	-0.066126	-0.002741
6.20	309.1328	16	22	6	-0.027305	0.043092	-0.156945	-0.111482	-0.031599
6.28	141.0176	7	6	3	0.017900	0.049231	-0.039798	-0.019564	0.015989
6.28	277.1065	15	18	5	-0.012717	0.050411	-0.128969	-0.088201	-0.016568
6.33	263.0899	14	16	5	-0.001244	0.058703	-0.111637	-0.072924	-0.004901
6.52	319.1168	6	8	1	-0.022384	-0.000580	-0.062536	-0.048455	-0.023714
6.57	153.0534	11	12	3	-0.008262	0.035337	-0.088551	-0.060395	-0.010922
6.59	307.1168	16	20	6	-0.012082	0.057858	-0.140879	-0.095712	-0.016348
6.75	293.1013	15	18	6	-0.001738	0.065021	-0.124676	-0.081563	-0.005810
6.80	271.0949	20	24	5	-0.037623	0.040498	-0.181484	-0.131034	-0.042388
6.88	307.1172	16	20	6	-0.012479	0.057461	-0.141276	-0.096108	-0.016745
6.92	309.1127	11	10	4	0.017711	0.064484	-0.068421	-0.038216	0.014858
6.95	227.0338	13	8	4	0.041594	0.093356	-0.053728	-0.020300	0.038436
7.04	293.1161	10	10	4	0.013473	0.057522	-0.067643	-0.039197	0.010787
7.06	249.0750	13	14	5	0.008467	0.065233	-0.096068	-0.059409	0.005005
7.08	349.1261	25	18	2	-0.005974	0.073502	-0.152330	-0.101005	-0.010821
7.13	319.1163	9	12	4	-0.005866	0.035916	-0.082809	-0.055826	-0.008414
7.15	331.1174	6	8	1	-0.022521	-0.000717	-0.062673	-0.048592	-0.023851
7.20	323.1119	16	20	7	-0.001301	0.072270	-0.136784	-0.089272	-0.005789
7.28	275.0912	9	8	3	0.013006	0.050243	-0.055566	-0.031519	0.010735
7.30	295.1321	14	14	4	0.000445	0.056304	-0.102421	-0.066347	-0.002962
7.34	321.0962	10	10	3	0.002235	0.042653	-0.072196	-0.046094	-0.000231
7.35	297.1482	10	10	3	0.003089	0.043506	-0.071341	-0.045240	0.000624
7.35	341.1040	10	8	4	0.029482	0.073072	-0.050791	-0.022641	0.026823
7.52	181.0492	11	10	4	0.017666	0.064438	-0.068467	-0.038261	0.014813
7.56	189.0909	12	14	2	-0.029311	0.013839	-0.108773	-0.080907	-0.031943
7.56	295.1317	20	22	8	0.009150	0.097705	-0.153926	-0.096738	0.003749
7.58	361.1274	9	12	4	-0.005218	0.036564	-0.082160	-0.055178	-0.007766
7.70	415.1385	16	18	5	-0.008433	0.057419	-0.129702	-0.087174	-0.012450
7.76	261.1114	15	18	4	-0.023483	0.036015	-0.133049	-0.094626	-0.027112
7.76	305.1013	10	12	4	-0.001902	0.042604	-0.083861	-0.055119	-0.004617
7.95	403.1397	12	14	4	-0.006987	0.043424	-0.099820	-0.067264	-0.010061
7.99	295.1346	8	10	3	-0.005067	0.029903	-0.069465	-0.046882	-0.007200
7.99	389.1256	8	10	3	-0.006333	0.028637	-0.070732	-0.048148	-0.008466
8.18	177.0772	7	14	5	-0.020066	0.020357	-0.094505	-0.068400	-0.022531
8.22	325.1099	9	10	3	-0.001607	0.036087	-0.071021	-0.046679	-0.003906
8.30	415.1423	7	6	3	0.017595	0.048926	-0.040103	-0.019869	0.015684
8.44	299.1673	19	24	3	-0.065497	0.002638	-0.190971	-0.146969	-0.069653
8.54	361.1684	20	26	6	-0.043880	0.038329	-0.195270	-0.142180	-0.048894
8.65	415.1432	15	28	13	0.001015	0.095476	-0.172937	-0.111934	-0.004747
8.68	403.1433	14	28	13	-0.003406	0.088331	-0.172342	-0.113098	-0.009002

8.85	317.1054	23	46	2	-0.220831	-0.140398	-0.368950	-0.317007	-0.225737
8.85	419.1746	15	32	13	-0.028821	0.066555	-0.204458	-0.142864	-0.034638
8.89	415.1421	8	8	4	0.019595	0.057738	-0.050647	-0.026014	0.017268
9.00	165.0555	7	8	3	0.004255	0.036044	-0.054285	-0.033755	0.002316
9.06	433.1519	17	20	6	-0.007203	0.065460	-0.141016	-0.094090	-0.011636
9.17	137.0249	8	8	3	0.008310	0.042823	-0.055246	-0.032957	0.006205
9.51	313.1462	8	10	3	-0.006013	0.028957	-0.070411	-0.047827	-0.008146
9.75	137.0246	16	16	4	-0.003351	0.058413	-0.117090	-0.077203	-0.007118

Table S6: Variables used for KMD-PCA-QDA classification of the detected peaks in the monomer fraction of the birch oil

#C: number of carbon atoms; #H: number of hydrogen atoms; #O: number of oxygen atoms. Kendrick mass defects are calculated for phenol (C_6H_5O), methoxy/primary alcohol (CH_3O), carboxylic acid (CHO_2), aldehyde (CHO) and secondary alcohol (CH_2O) groups

					Kendrick mass defect				
**Retention time in SFC/QToF-MS /min**	**Exact mass**	**#C**	**#H**	**#O**	**Phenol**	**Methoxy/primary alcohol**	**Carboxylic acid**	**Aldehyde**	**Secondary alcohol**
2.50	151.0394	6	8	3	-0.000212	0.028853	-0.053736	-0.034965	-0.001984
3.00	135.0455	9	10	4	0.008198	0.049523	-0.067903	-0.041215	0.005677
3.00	155.0707	7	10	3	-0.011265	0.020981	-0.070648	-0.049823	-0.013232
3.02	141.0552	7	10	3	-0.011151	0.021096	-0.070533	-0.049709	-0.013118
3.26	121.0290	7	6	2	0.007698	0.035399	-0.043313	-0.025424	0.006009
3.31	135.0447	8	6	3	0.023320	0.057375	-0.039394	-0.017401	0.021243
3.31	141.0557	9	6	3	0.027596	0.064375	-0.040133	-0.016382	0.025353
3.43	181.0504	7	6	2	0.007858	0.035559	-0.043153	-0.025264	0.006169
3.47	151.0026	9	8	3	0.012579	0.049816	-0.055993	-0.031946	0.010308
3.50	207.0664	10	8	3	0.017046	0.057006	-0.056542	-0.030736	0.014609
3.65	151.0394	8	8	2	-0.002852	0.028030	-0.059722	-0.039779	-0.004736
3.81	159.0658	10	10	2	-0.008714	0.028073	-0.076459	-0.052702	-0.010958
3.88	163.0391	9	8	2	0.001935	0.035541	-0.059951	-0.038248	-0.000115
3.88	177.0548	8	8	2	-0.002944	0.027939	-0.059814	-0.039870	-0.004827
3.88	225.1604	11	10	3	0.005840	0.048982	-0.073607	-0.045746	0.003208
3.89	121.0288	7	10	2	-0.022084	0.006531	-0.074781	-0.056301	-0.023830
3.93	149.0239	10	10	3	0.002387	0.042805	-0.072043	-0.045942	-0.000078
3.96	197.0818	10	8	4	0.028376	0.071967	-0.051898	-0.023747	0.025717
4.00	135.0446	11	8	4	0.032202	0.078517	-0.053088	-0.023178	0.029377
4.02	177.0550	10	10	3	0.002052	0.042470	-0.072379	-0.046277	-0.000414
4.07	205.0501	13	8	4	0.041014	0.092777	-0.054308	-0.020880	0.037857
4.09	165.0553	7	8	3	0.003729	0.035518	-0.054811	-0.034282	0.001790
4.19	225.1603	9	8	3	0.012945	0.050182	-0.055627	-0.031580	0.010674
4.28	167.0703	7	10	2	-0.022222	0.006394	-0.074918	-0.056438	-0.023967
4.31	177.0551	8	8	3	0.008204	0.042716	-0.055352	-0.033064	0.006098
4.36	125.0602	7	10	3	-0.011723	0.020524	-0.071106	-0.050281	-0.013690
4.36	155.0703	9	6	3	0.027588	0.064367	-0.040141	-0.016389	0.025345
4.43	141.0551	9	12	3	-0.017326	0.020826	-0.087583	-0.062945	-0.019653
4.43	161.0600	8	12	3	-0.021144	0.014283	-0.086385	-0.063506	-0.023305
4.45	137.0239	13	22	3	-0.085618	-0.034281	-0.180156	-0.147003	-0.088749
4.45	147.0442	8	8	3	0.008234	0.042747	-0.055322	-0.033034	0.006129
4.45	163.0394	10	8	4	0.027735	0.071326	-0.052539	-0.024388	0.025077
4.54	125.0603	13	22	3	-0.085587	-0.034250	-0.180126	-0.146972	-0.088718
4.55	167.0708	6	8	2	-0.011687	0.013748	-0.058525	-0.042099	-0.013238
4.55	189.0557	8	6	3	0.022946	0.057001	-0.039767	-0.017775	0.020869
4.57	127.0391	11	12	4	0.002008	0.049238	-0.084968	-0.054466	-0.000873
4.57	207.0661	11	12	4	0.001741	0.048971	-0.085235	-0.054733	-0.001140

4.62	149.0236	10	14	4	-0.017278	0.027686	-0.100080	-0.071042	-0.020021
4.64	163.0392	9	6	4	0.038774	0.079183	-0.035642	-0.009545	0.036309
4.64	177.0546	12	10	4	0.021599	0.071095	-0.069550	-0.037585	0.018580
4.67	111.0447	9	10	3	-0.002545	0.035149	-0.071960	-0.047617	-0.004844
4.69	203.0345	9	12	3	-0.016754	0.021398	-0.087011	-0.062372	-0.019081
4.74	191.0339	7	6	3	0.018563	0.049895	-0.039134	-0.018901	0.016652
4.78	163.0398	11	10	3	0.006663	0.049805	-0.072783	-0.044922	0.004032
4.79	175.0398	13	22	3	-0.085313	-0.033976	-0.179851	-0.146698	-0.088444
4.84	139.0395	9	8	3	0.012228	0.049465	-0.056344	-0.032297	0.009957
4.88	191.0345	8	12	3	-0.021556	0.013872	-0.086797	-0.063918	-0.023717
4.91	175.0400	10	10	3	0.001808	0.042226	-0.072623	-0.046521	-0.000658
4.93	189.0549	10	8	3	0.016413	0.056373	-0.057175	-0.031369	0.013975
4.93	215.0715	10	10	3	0.002509	0.042927	-0.071921	-0.045819	0.000044
4.95	161.0237	9	8	3	0.012777	0.050014	-0.055795	-0.031747	0.010506
5.09	177.0184	10	10	3	0.002128	0.042546	-0.072303	-0.046201	-0.000337
5.19	161.0237	8	8	2	-0.003706	0.027176	-0.060577	-0.040633	-0.005590
5.19	177.0553	10	8	3	0.016649	0.056610	-0.056939	-0.031132	0.014212
5.21	175.0394	13	12	3	-0.000442	0.048605	-0.090763	-0.059089	-0.003433
5.38	217.0502	7	4	4	0.044971	0.079476	-0.018569	0.003714	0.042867
6.81	225.1601	11	10	4	0.017254	0.064026	-0.068879	-0.038673	0.014401
6.93	227.0344	7	12	4	-0.015227	0.021108	-0.082138	-0.058673	-0.017443

Table S7: Variables used for KMD-PCA-QDA classification of the detected peaks in the dimer fraction of the birch oil

#C: number of carbon atoms; #H: number of hydrogen atoms; #O: number of oxygen atoms. Kendrick mass defects are calculated for phenol (C_6H_5O), methoxy/primary alcohol (CH_3O), carboxylic acid (CHO_2), aldehyde (CHO) and secondary alcohol (CH_2O) groups. Green shading denotes the masses which were classified as dimers and an asterisk (*) marks dimers which were detected by the CAD.

					Kendrick mass defect				
**Retention time in SFC/QToF-MS /min**	**Exact mass**	**#C**	**#H**	**#O**	**Phenol**	**Methoxy/primary alcohol**	**Carboxylic acid**	**Aldehyde**	**Secondary alcohol**
2.84	171.0999	9	16	4	-0.035610	0.007087	-0.114238	-0.086665	-0.038215
2.84	223.0587	14	18	6	-0.006418	0.057617	-0.124341	-0.082987	-0.010324
3.17	155.0328	16	18	6	0.003537	0.073020	-0.124416	-0.079545	-0.000701
3.31	193.0484	11	12	4	0.004349	0.051579	-0.082625	-0.052124	0.001468
3.52	207.0638	10	10	4	0.014602	0.058650	-0.066514	-0.038068	0.011915
3.53	193.0487	10	10	4	0.014297	0.058345	-0.066819	-0.038373	0.011610
3.53	289.1797	16	22	6	-0.027809	0.042589	-0.157448	-0.111985	-0.032103
4.07	205.0495	16	20	6	-0.012540	0.057400	-0.141337	-0.096169	-0.016806
4.09	191.0714	15	20	6	-0.017358	0.049859	-0.141139	-0.097730	-0.021457
4.19	263.0921	16	22	6	-0.027427	0.042970	-0.157067	-0.111604	-0.031721
4.23	165.0542	18	22	6	-0.019058	0.056788	-0.158729	-0.109748	-0.023684
4.33	219.0658	15	16	5	0.002750	0.065421	-0.112660	-0.072187	-0.001073
4.50	217.0853	13	16	5	-0.007374	0.049850	-0.112752	-0.075797	-0.010864
4.52	125.0962	11	10	4	0.018093	0.064865	-0.068040	-0.037834	0.015240
4.52	187.0964	13	14	3	-0.013468	0.036036	-0.104632	-0.072662	-0.016488
4.52	209.0800	11	12	5	0.015283	0.066143	-0.078378	-0.045532	0.012180
4.57	203.0703	19	20	3	-0.031528	0.035692	-0.155315	-0.111904	-0.035628
4.59	207.0649	17	20	5	-0.019769	0.049265	-0.146896	-0.102314	-0.023980
4.67	303.1230	16	20	6	-0.013043	0.056897	-0.141840	-0.096673	-0.017309
4.81	205.0493	15	18	5	-0.013632	0.049496	-0.129885	-0.089117	-0.017483
4.91	245.0814	18	26	3	-0.081511	-0.015643	-0.202810	-0.160272	-0.085529
*5.14	333.1333	16	20	7	-0.000752	0.072819	-0.136234	-0.088723	-0.005240
5.16	217.0491	19	22	7	-0.002407	0.079793	-0.153780	-0.100695	-0.007420
5.18	309.1333	11	12	4	0.003205	0.050435	-0.083770	-0.053269	0.000324
5.23	231.0656	19	22	4	-0.035207	0.036101	-0.166522	-0.120472	-0.039557
5.38	295.1177	15	20	5	-0.028566	0.035020	-0.145661	-0.104597	-0.032444
5.40	217.0496	12	10	4	0.022163	0.071659	-0.068985	-0.037021	0.019144
5.47	251.0916	7	8	4	0.016333	0.051752	-0.048893	-0.026019	0.014172
5.66	279.1231	12	12	4	0.006681	0.056634	-0.085311	-0.053050	0.003634
5.91	275.0918	12	12	3	-0.003643	0.042680	-0.088948	-0.059032	-0.006468
5.91	281.1016	19	18	5	0.006188	0.080211	-0.130128	-0.082324	0.001673
6.01	277.1074	8	14	1	-0.058021	-0.029397	-0.110733	-0.092247	-0.059767
6.20	309.1329	12	10	4	0.022666	0.072162	-0.068482	-0.036517	0.019647
6.59	307.1173	16	18	7	0.012869	0.085983	-0.121771	-0.074554	0.008410
6.88	307.1178	22	24	8	0.006659	0.101118	-0.167291	-0.106289	0.000897
6.92	309.1122	13	12	4	0.011331	0.064008	-0.085677	-0.051657	0.008118

7.25	323.1113	19	18	4	-0.006713	0.063680	-0.136344	-0.090884	-0.011007
7.30	275.0903	21	24	8	0.002116	0.093851	-0.166818	-0.107575	-0.003480
7.37	321.0970	14	14	4	0.000597	0.056456	-0.102268	-0.066195	-0.002810
7.54	295.1319	9	10	3	-0.001409	0.036285	-0.070823	-0.046480	-0.003708
7.58	361.1269	11	12	3	-0.009147	0.034453	-0.089436	-0.061280	-0.011806
*7.78	305.1005	15	16	5	0.001255	0.063926	-0.114155	-0.073682	-0.002567
7.95	403.1378	11	10	4	0.017864	0.064636	-0.068268	-0.038063	0.015011
*8.25	325.1051	11	14	4	-0.011148	0.036539	-0.098966	-0.068169	-0.014057
8.89	415.1376	9	16	3	-0.044942	-0.005876	-0.116884	-0.091655	-0.047325
9.54	313.1422	14	16	5	-0.003502	0.056446	-0.113896	-0.075182	-0.007158

Figure S7: Score plots of the KMD-PCA-QDA classification model of dimers. Overlaid green stars in figures A, B and C denote the detected peaks in the unfractionated birch oil, the as well as the monomer and the dimer fractions of the birch oil, respectively

A

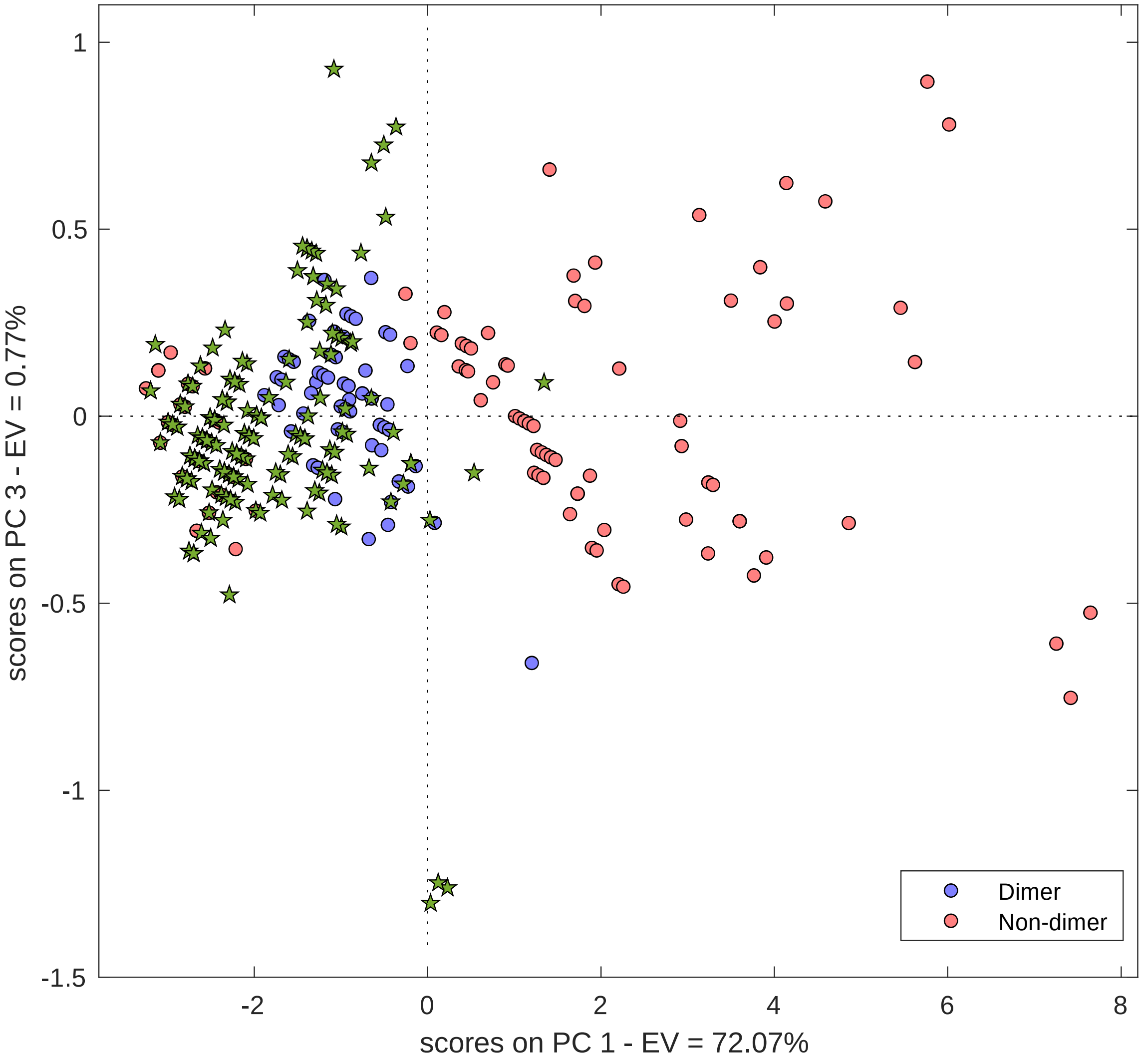

B

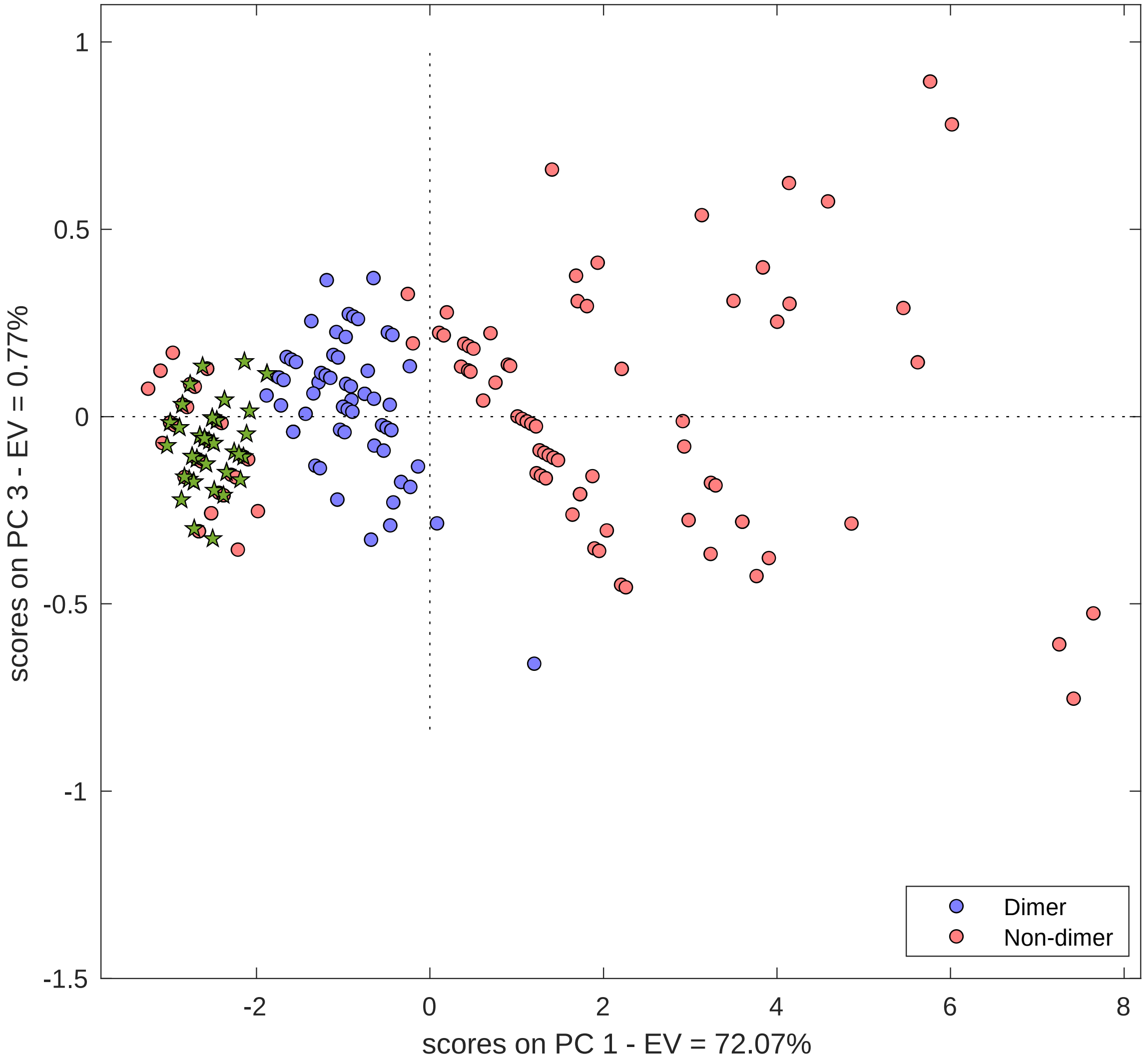

1
0.5
0
-0.5
-1
-1.5
-2
0
2
4
6
8
scores on PC 3 - EV = 0.77%
scores on PC 1 - EV = 72.07%
Dimer
Non-dimer

C

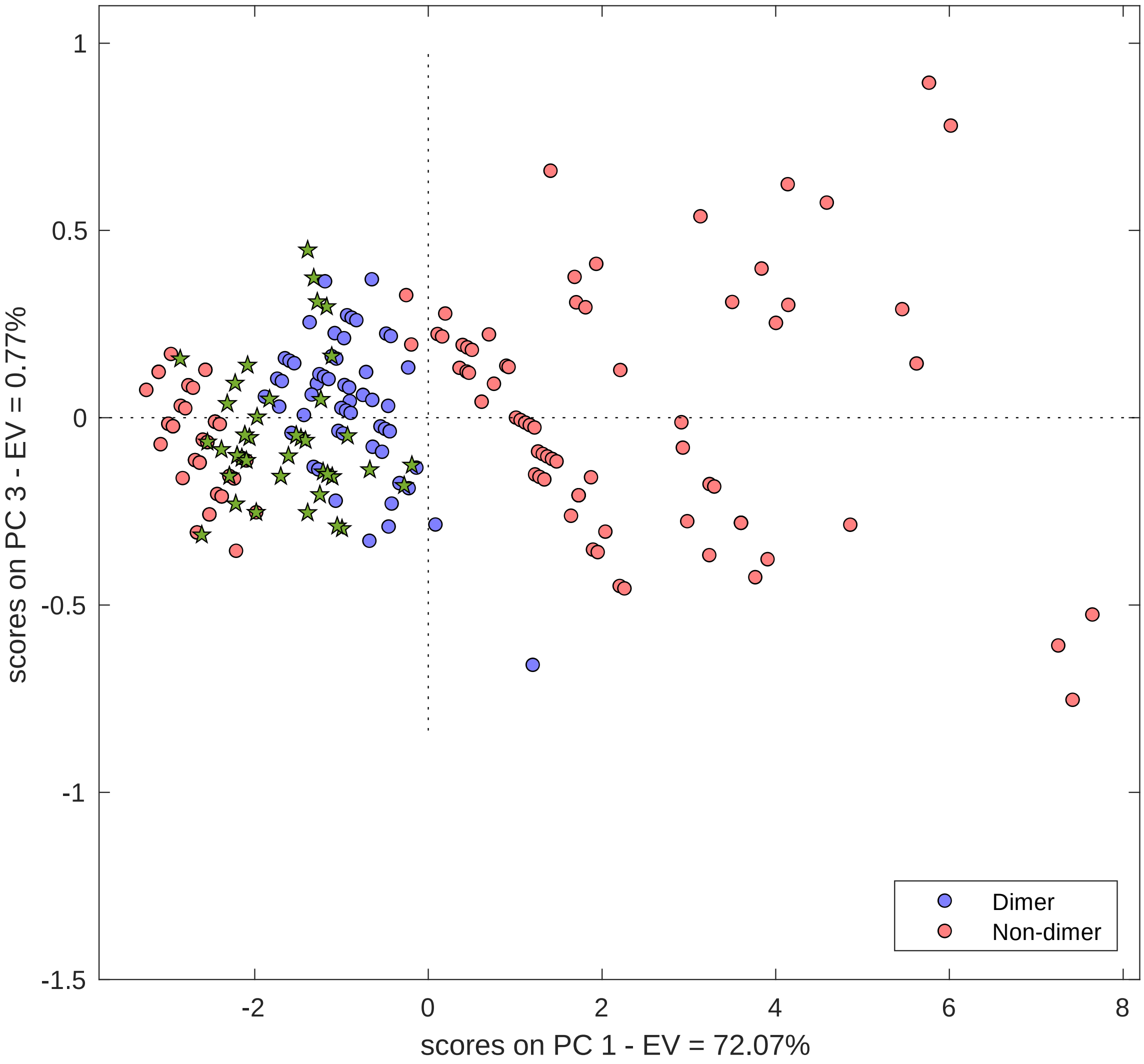

1
0.5
0
-0.5
-1
-1.5
-2
0
2
4
6
8
scores on PC 3 - EV = 0.77%
scores on PC 1 - EV = 72.07%
Dimer
Non-dimer

Figure S8: ESI-QToF mass spectra of the three dimers quantified by the CAD. Mass spectra in A, B and C belong to dimers detected at 5.08 min, 7.78 min and 8.22 min by the CAD, respectively

A

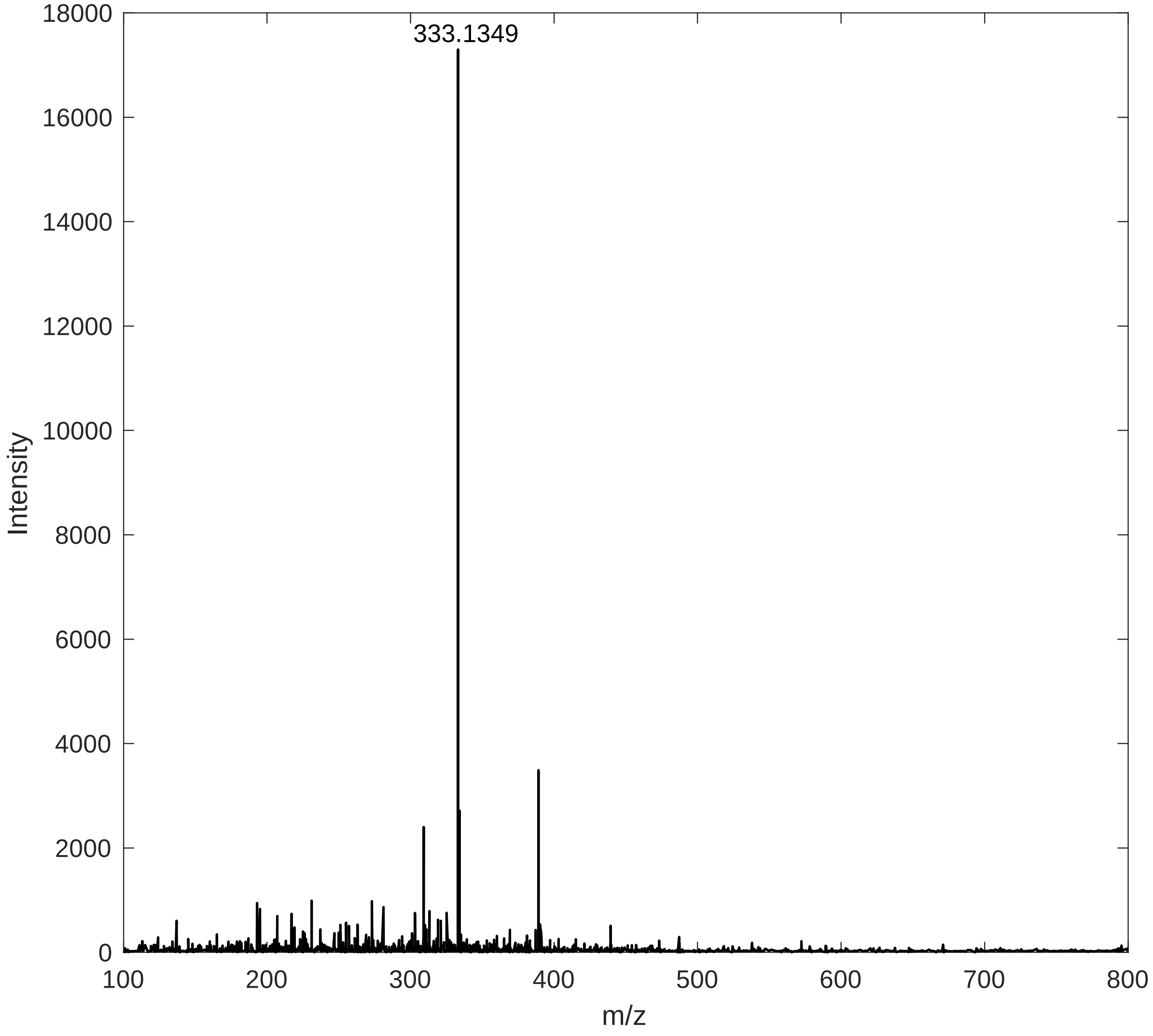

B

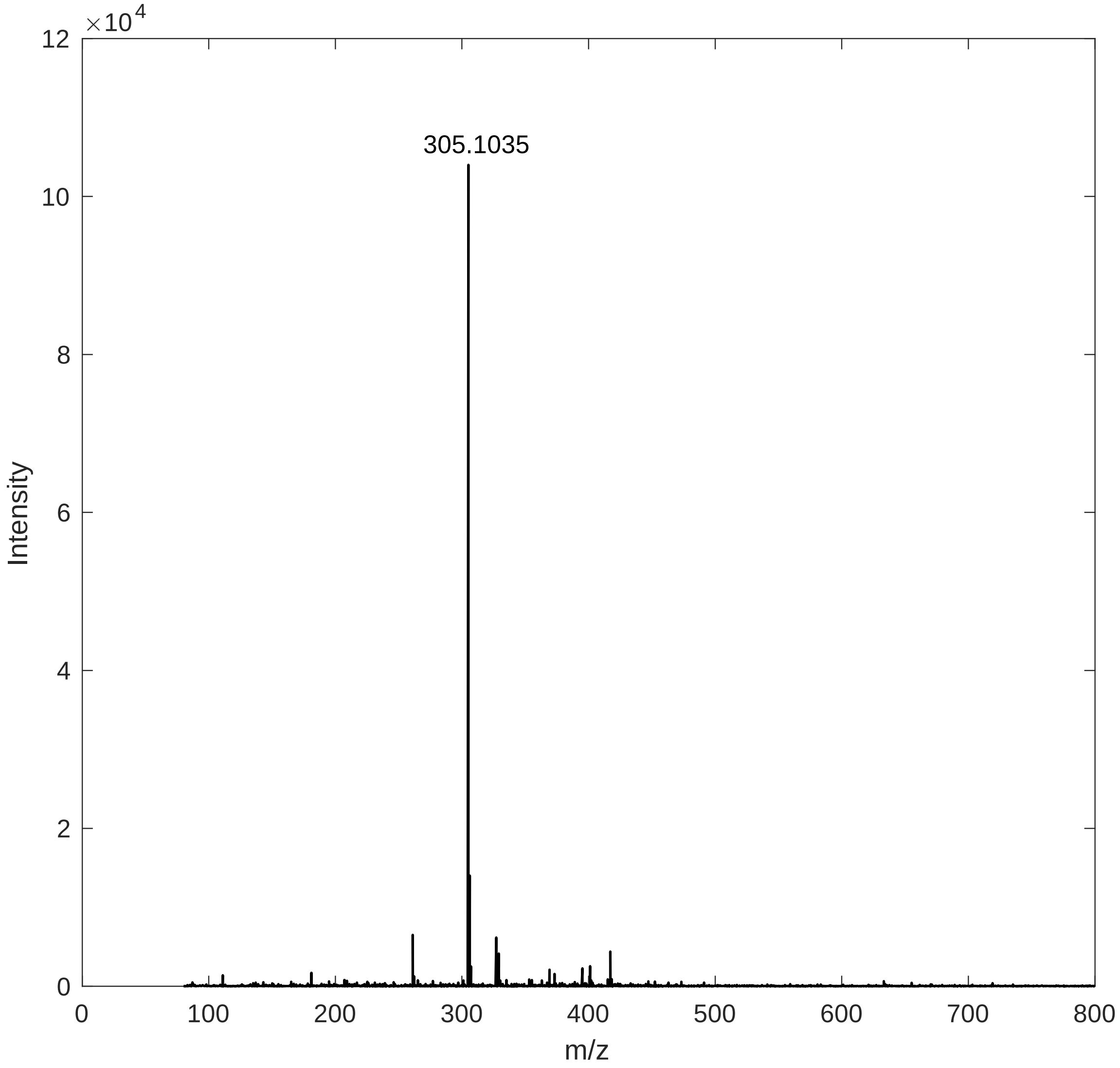
×10^4
12
10
8
6
4
2
0
Intensity
305.1035
0
100
200
300
400
500
600
700
800
m/z

C

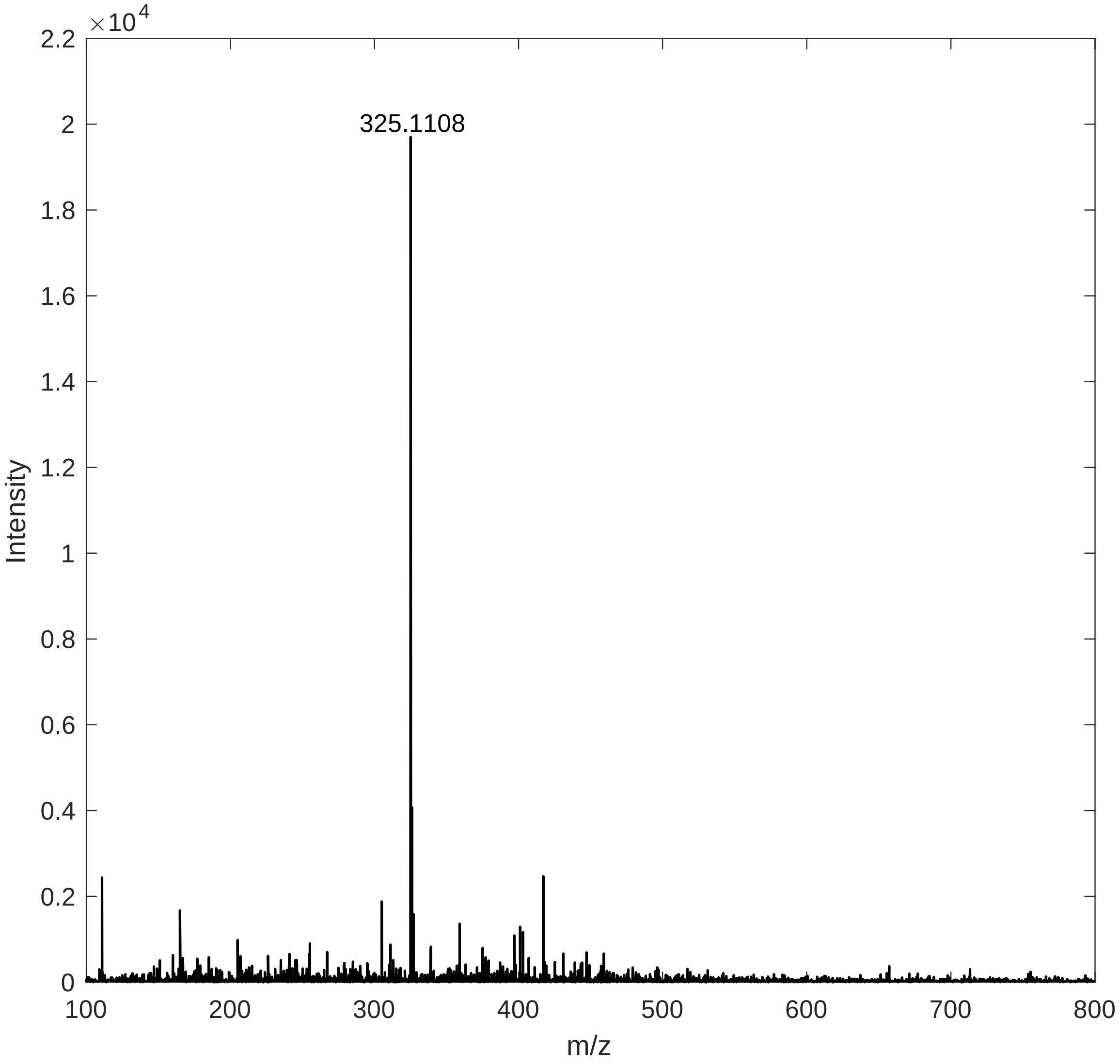
×10^4
2.2
2
1.8
1.6
1.4
1.2
1
0.8
0.6
0.4
0.2
0
Intensity
325.1108
100
200
300
400
500
600
700
800
m/z

Figure S9: SFC-DAD (A), SFC/CAD (B) chromatograms and score plot of the KMD-PCA-QDA dimer classification (C) of the dimer fraction of the spruce oil

The SFC-CAD chromatogram was recorded at 210 nm and is presented without blank correction, while the CAD signal is presented after the peak sharpening routine. In the score plot, the detected peaks in the sample are overlaid as green stars.

A

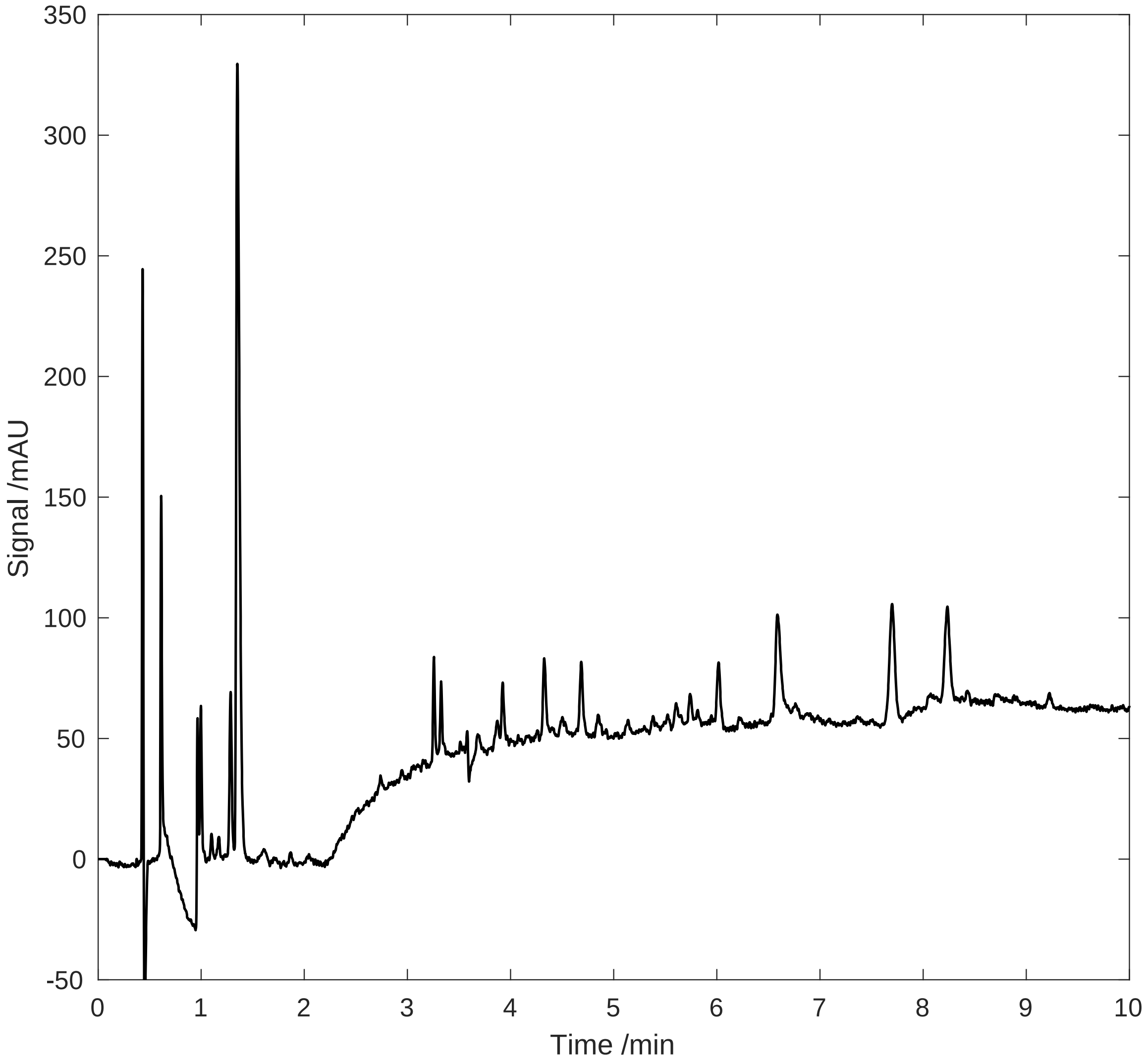

B

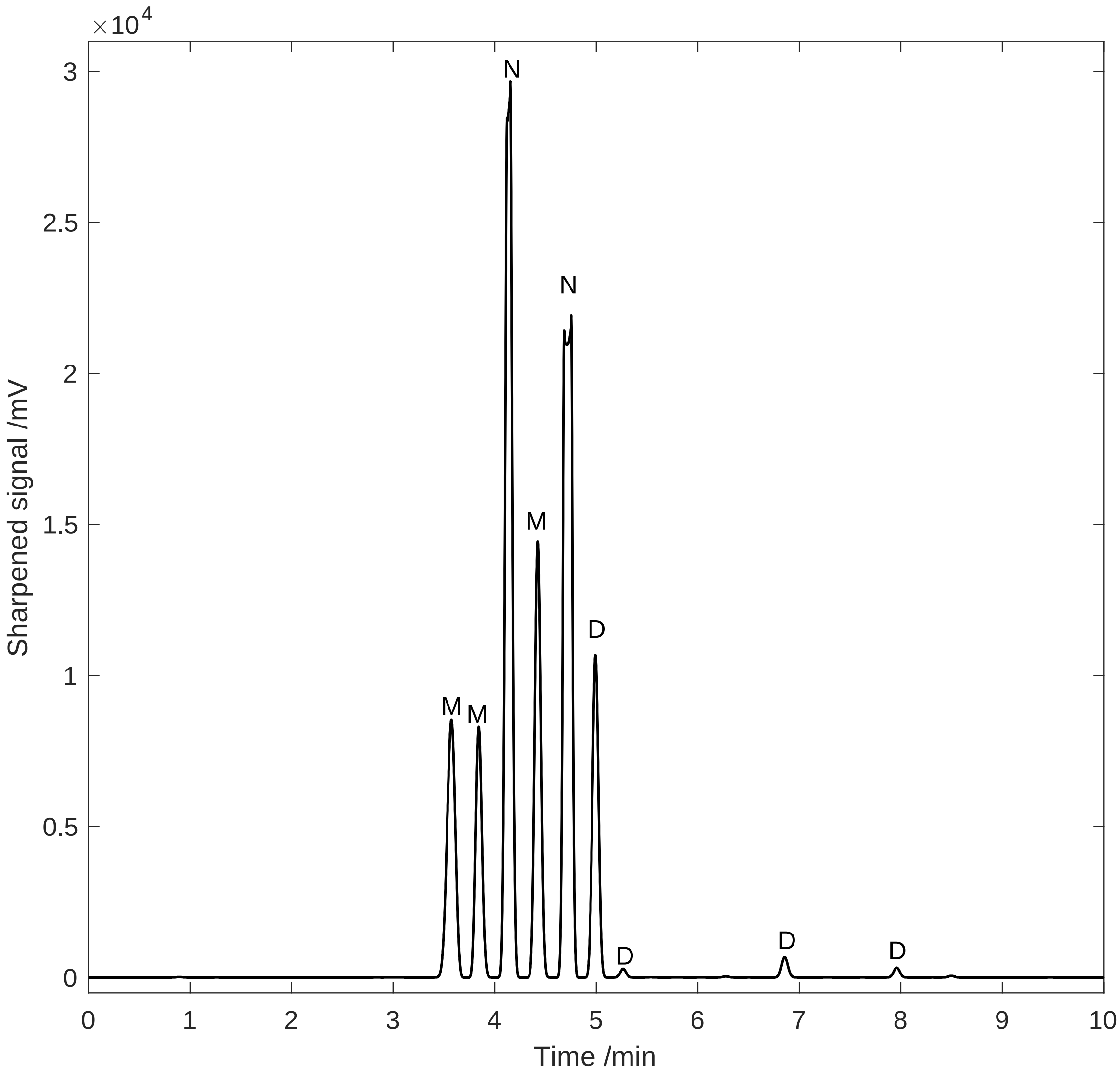

×10^4
3
2.5
2
1.5
1
0.5
0
Sharpened signal /mV
N
N
M
D
M
M
D
D
D
0
1
2
3
4
5
6
7
8
9
10
Time /min

C

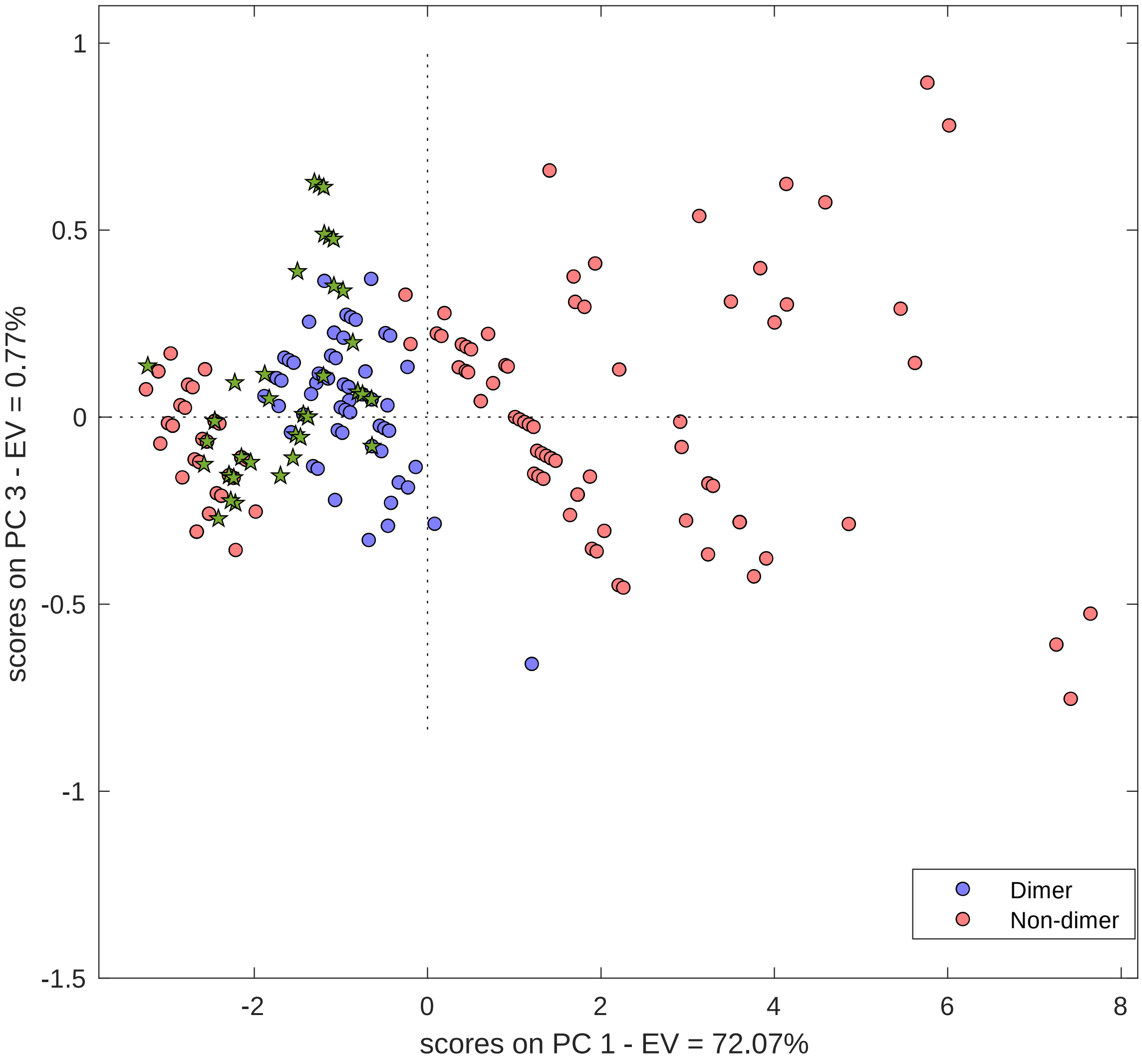

1
0.5
0
-0.5
-1
-1.5
-2
0
2
4
6
8
scores on PC 3 - EV = 0.77%
scores on PC 1 - EV = 72.07%
Dimer
Non-dimer

Table S8: Variables used for KMD-PCA-QDA classification of the detected peaks in the dimer fraction of the spruce oil

#C: number of carbon atoms; #H: number of hydrogen atoms; #O: number of oxygen atoms. Kendrick mass defects are calculated for phenol (C_6H_5O), methoxy/primary alcohol (CH_3O), carboxylic acid (CHO_2), aldehyde (CHO) and secondary alcohol (CH_2O) groups. Green shading denotes the masses which were classified as dimers and and an asterisk (*) marks dimers which were detected by the CAD.

					Kendrick mass defect				
Retention time in SFC/QToF-MS /min	Exact mass	#C	#H	#O	Phenol	Methoxy/primary alcohol	Carboxylic acid	Aldehyde	Secondary alcohol
1.39	255.2328	16	32	2	-0.147048	-0.088885	-0.254157	-0.216595	-0.150596
1.45	225.1606	13	22	3	-0.085877	-0.034540	-0.180416	-0.147262	-0.089008
2.21	193.0496	10	10	4	0.013405	0.057453	-0.067712	-0.039265	0.010718
3.31	193.0485	10	10	4	0.014541	0.058589	-0.066575	-0.038129	0.011854
3.31	301.2149	20	30	2	-0.112310	-0.043709	-0.238640	-0.194337	-0.116494
3.43	299.1990	20	28	2	-0.097133	-0.028990	-0.222620	-0.178613	-0.101289
3.50	207.0641	11	12	4	0.003998	0.051228	-0.082976	-0.052475	0.001118
3.52	193.0484	10	10	4	0.014640	0.058688	-0.066476	-0.038029	0.011953
3.52	297.1832	20	26	2	-0.082062	-0.014377	-0.206706	-0.162995	-0.086190
3.59	195.0647	10	12	4	-0.000972	0.043534	-0.082931	-0.054189	-0.003687
3.88	177.0538	10	10	3	0.003333	0.043750	-0.071097	-0.044996	0.000868
3.96	211.0953	11	16	4	-0.025670	0.022475	-0.114329	-0.083238	-0.028606
4.04	177.0532	10	10	3	0.003920	0.044338	-0.070510	-0.044408	0.001455
4.21	185.0793	9	14	4	-0.019182	0.023057	-0.096967	-0.069689	-0.021759
4.23	313.1771	20	26	3	-0.070137	0.001179	-0.201467	-0.155411	-0.074487
4.26	315.1920	20	28	3	-0.084323	-0.012550	-0.216495	-0.170144	-0.088701
4.33	177.0529	10	10	3	0.004218	0.044635	-0.070212	-0.044111	0.001752
4.38	171.0634	8	12	4	-0.008472	0.030586	-0.080398	-0.055175	-0.010854
4.38	155.0686	8	12	3	-0.019505	0.015923	-0.084745	-0.061866	-0.021666
4.42	103.0378	7	4	1	-0.007754	0.015863	-0.051245	-0.035993	-0.009194
4.43	177.0528	10	10	3	0.004309	0.044726	-0.070121	-0.044019	0.001844
4.47	165.0531	9	10	3	-0.000349	0.037345	-0.069762	-0.045420	-0.002648
4.54	187.0943	9	16	4	-0.033490	0.009206	-0.112117	-0.084544	-0.036095
4.57	311.1615	20	24	3	-0.055280	0.015578	-0.185767	-0.140007	-0.059602
4.57	203.0685	12	12	3	-0.001843	0.044480	-0.087147	-0.057232	-0.004668
*4.91	245.0783	14	14	4	0.003732	0.059590	-0.099133	-0.063059	0.000325
5.04	265.1034	14	18	5	-0.013996	0.046407	-0.125231	-0.086222	-0.017680
*5.16	325.1408	20	22	4	-0.029505	0.044527	-0.165835	-0.118026	-0.034020
5.49	251.0899	13	16	5	-0.005650	0.051573	-0.111027	-0.074073	-0.009140
5.80	343.1506	20	24	5	-0.032681	0.045438	-0.176540	-0.126090	-0.037446
6.03	277.1053	15	18	5	-0.011558	0.051570	-0.127810	-0.087042	-0.015408
6.18	289.1057	16	18	5	-0.007533	0.058319	-0.128801	-0.086274	-0.011550
6.44	329.1741	20	26	4	-0.061324	0.013623	-0.199340	-0.150940	-0.065895
6.49	287.0909	16	16	5	0.006561	0.071956	-0.113865	-0.071633	0.002572
6.71	289.1066	16	18	5	-0.008433	0.057419	-0.129702	-0.087174	-0.012450
*6.81	357.1315	20	22	6	-0.008507	0.072786	-0.158210	-0.105711	-0.013466

*6.81	313.1059	18	18	5	0.001065	0.072364	-0.130235	-0.084190	-0.003284
7.16	371.1129	20	20	7	0.015255	0.099721	-0.140292	-0.085744	0.010103
7.28	275.0917	15	16	5	0.001286	0.063957	-0.114124	-0.073652	-0.002537
*7.94	355.1181	20	20	6	0.004245	0.085081	-0.144616	-0.092413	-0.000686
8.42	355.1186	20	20	6	0.003711	0.084547	-0.145150	-0.092947	-0.001220
8.54	361.1647	20	26	6	-0.040158	0.042050	-0.191547	-0.138457	-0.045173

References

(1) Curti, C.; Zanardi, F.; Battistini, L.; Sartori, A.; Rassu, G.; Pinna, L.; Casiraghi, G.; Farmaceutico, D.; Uni, V.; Usberti, V. G. P.; et al. Streamlined , Asymmetric Synthesis of 8 , 4 ′ -Oxyneolignans. *J. Org. Chem.* **2006**, *71*, 8552–8558.

(2) Lancefield, C. S.; Ojo, O. S.; Tran, F.; Westwood, N. J. Isolation of Functionalized Phenolic Monomers through Selective Oxidation and C-O Bond Cleavage of the Β-O-4 Linkages in Lignin. *Angew. Chemie* **2015**, *127* (1), 260–264. https://doi.org/10.1002/ange.201409408.

(3) Klein, I.; Marcum, C.; Kenttämaa, H.; Abu-Omar, M. M. Mechanistic Investigation of the Zn/Pd/C Catalyzed Cleavage and Hydrodeoxygenation of Lignin. *Green Chem.* **2016**, *18* (8), 2399–2405. https://doi.org/10.1039/c5gc01325a.

(4) Subbotina, E.; Galkin, M. V.; Samec, J. S. M. Pd/C-Catalyzed Hydrogenolysis of Dibenzodioxocin Lignin Model Compounds Using Silanes and Water as Hydrogen Source. *ACS Sustain. Chem. Eng.* **2017**, *5* (5), 3726–3731. https://doi.org/10.1021/acssuschemeng.7b00428.

(5) Ferré-Filmon, K.; Delaude, L.; Demonceau, A.; Noels, A. F. Stereoselective Synthesis of (E)-Hydroxystilbenoids by Ruthenium-Catalyzed Cross-Metathesis. *European J. Org. Chem.* **2005**, No. 15, 3319–3325. https://doi.org/10.1002/ejoc.200500068.

(6) Matsushita, Y.; Yagami, S.; Kato, A.; Mitsuda, H.; Aoki, D.; Fukushima, K. Combinations of the Aromatic Rings in β-1 Structure Formation of Lignin Based on Quantitative Analysis by Thioacidolysis. *J. Agric. Food Chem.* **2020**, *68* (34), 9245–9251. https://doi.org/10.1021/acs.jafc.0c03206.

(7) Lancefield, C. S.; Westwood, N. J. The Synthesis and Analysis of Advanced Lignin Model Polymers. *Green Chem.* **2015**, *17* (11), 4980–4990. https://doi.org/10.1039/c5gc01334h.

(8) Reale, S.; Attanasio, F.; Spreti, N.; De Angelis, F. Lignin Chemistry: Biosynthetic Study and Structural Characterisation of Coniferyl Alcohol Oligomers Formed in Vitro in a Micellar Environment. *Chem. - A Eur. J.* **2010**, *16* (20), 6077–6087. https://doi.org/10.1002/chem.200903302.

(9) Dao Thi, H.; Van Aelst, K.; Van den Bosch, S.; Katahira, R.; Beckham, G. T.; Sels, B. F.; Van Geem, K. M. Identification and Quantification of Lignin Monomers and Oligomers from Reductive Catalytic Fractionation of Pine Wood with GC × GC – FID/MS. *Green Chem.* **2022**, *24* (1), 191–206. https://doi.org/10.1039/d1gc03822b.
